# A pan-viral map of host dependency factors from multi-omics integration and machine learning across influenza A, SARS-CoV-2, Zika, and dengue viruses

**DOI:** 10.1186/s12967-026-08197-9

**Published:** 2026-05-02

**Authors:** Mohadeseh Naseri, Alicia Hiemisch, André Dietz, Marcus Oswald, Rainer Koenig

**Affiliations:** Institute for Infectious Diseases and Infection Control (IIMK), University Hospital, Jena, Germany

**Keywords:** SARS-CoV-2, Zika, Dengue, Influenza A, Host dependency factors, Deep learning

## Abstract

**Background:**

Host dependency factors (HDF) are essential for viral replication and are promising targets for broad-spectrum antivirals. However, most work has focused on individual viruses or individual data types, limiting our understanding of shared host mechanisms across viruses.

**Methods:**

We developed a pan–viral framework that integrates multi–omics data–including genome-wide perturbation screens, single–cell transcriptomes and viral interactomes–and combines graph–based learning with classical machine–learning models to prioritize HDF for four RNA viruses (SARS-CoV–2, influenza A virus, dengue virus and Zika virus).

**Results:**

Across viruses, the framework achieved high discrimination, with area under the receiver operating characteristic curve (ROC–AUC) greater than 0.90 on benchmark datasets, and identified a conserved signature of 118 genes shared by all four viruses and 427 genes shared by at least three. These genes converge on recurrent host programmes such as clathrin–mediated entry and endomembrane trafficking, nuclear transport, RNA processing and stress granules, and proteostasis and ubiquitin–proteasome signalling. The pan–viral signature generalizes beyond the training set, as genes shared by three or more viruses are strongly enriched among top–ranked Ebola virus candidates. We further provide a prioritized shortlist and an experimental validation roadmap to guide follow–up perturbation studies.

**Conclusions:**

Our integrative multi-omics and machine-learning approach outlines a prediction-based, data-driven map of pan-viral host liabilities and highlights tractable opportunities for host-directed therapy against diverse RNA viruses.

**Supplementary Information:**

The online version contains supplementary material available at 10.1186/s12967-026-08197-9.

## Introduction

In recent years, outbreaks of viral diseases—primarily caused by RNA viruses such as SARS-CoV-2, Zika virus (ZIKV), dengue virus (DENV), influenza A virus (IAV), and Ebola virus (EBOV)—have posed serious threats to the global public health [1]. For instance, the COVID-19 pandemic has resulted in over six million fatalities and disrupted societies worldwide [2]. Seasonal IAV epidemics contribute substantially to mortality, with the 2009 H1N1 pandemic infecting approximately 60 million Americans and causing over 120,000 deaths, while novel avian strains continue to pose a risk of human pandemics [3]. DENV infects an estimated 100–400 million individuals annually [4], leading to hemorrhagic fever and shock, whereas ZIKV is associated with congenital malformations and the Guillain–Barré syndrome [5]. Although EBOV outbreaks are less frequent, they are catastrophic, with case fatality rates around 50% [6]. These examples highlight the significant impact of RNA viruses on public health, often rendering conventional countermeasures insufficient to combat them. No approved antivirals exist for DENV, ZIKV and EBOV, no vaccines against ZIKV, and EBOV vaccines are limited to a single species [6], underscoring the necessity for broad-spectrum therapies against these RNA viruses. Additionally, the deployment of vaccines is often characterized by delays [7].

Considering preparedness for the next pandemic outbreak, a central medical gap is that antiviral development is still largely virus-specific and reactive. However, preparedness requires targetable host mechanisms that can be leveraged early before pathogen-tailored drugs and vaccines become available at scale. The majority of antiviral agents are designed to target viral proteins. However, viruses can readily evade these treatments through mutations, emphasizing the need for alternative strategies [7]. One promising approach is a host-directed therapy, which emphasizes the identification of host dependency factors (HDF) [8]. These comprise host genes and proteins that viruses exploit to enter, replicate, assemble, and exit the host cells. Host-directed therapy aims to interfere with HDF required for pathogen replication or persistence, and has been proposed as a strategy to broaden antiviral activity while potentially increasing the barrier to resistance compared with direct-acting antivirals [9–11]. Accordingly, the key challenge is not whether host dependencies exist, but how to robustly prioritize those that generalize across experimental contexts and—critically—across viruses.

In this study, we focus on the four major RNA viruses SARS-CoV-2, IAV, DENV, and ZIKV as a practical “pan-viral” model. They represent distinct transmission routes and disease contexts (respiratory and arboviral infections), while also offering a rich data source comprising publicly available multi-omics experimental observational data. Here, we refer to “pan-viral” as shared host liabilities among these four viruses, rather than an exhaustive list of HDF for all viruses. Most studies have focused on single viruses or individual data modalities, which fail to reveal shared host mechanisms across different viral infections. The limited overlap between CRISPR/Cas9 and RNAi knockout/down screens, even when focusing on a single virus, underscores the issue of data diversity in this field [3, 12]. Indeed, there are “consistency” issues in the experimental setups. Differences in perturbation technology, cell systems, viral isolates, multiplicity of infection (MOI), knockout library design, and analysis pipelines have led to rather incompatible hit lists. As a result, it becomes difficult to decide which candidates are reliable enough to prioritize for follow-up validation and, ultimately, therapeutic development. For IAV, Li et al. noted largely divergent host-factor results with few overlapping hits and introduced the linear regression based analysis model Meta-Analysis by Information Content (MAIC) to integrate data from heterogeneous methods [3]. For SARS-CoV-2, Rebendenne et al. showed strong cell-type specificity of hits across CRISPR based knockout screens, motivating to perform secondary screens with additional cell models to refine the hits from genome scale screens [13]. Even when evidence is systematically integrated across studies, the resulting rankings can remain context-dependent and are not designed to exploit gene characteristics, also on the cellular network level that may enable transferring evidences of HDF across virus entities [12, 14–16]. In summary, the field has accumulated substantial experimental data from gene perturbation screens, interactomes, and single-cell transcriptomes. However, the documented divergence across studies and cell types shows that simple overlap or rank aggregation is insufficient to identify robust, shared host dependencies. Comprehensive, but heterogenous datasets across the four viruses are available. Hence, computational efforts to concert their information, and a data-driven, integrative computationally derived map is needed to guide experimental directions. A key translational gap is the absence of an actionable cross-virus host-target map that is robust across heterogeneous experimental settings and directly paves the way to efficient experimental validation.

In response, we developed an integrative pipeline that leverages the strengths of multiple high throughput datasets. For each virus, we aggregated CRISPR/Cas9 screens, RNAi screens, viral protein/RNA–host protein interactomes and single-cell transcriptomics into a unified set of evidence-derived, virus-specific benchmark labels using the MAIC algorithm [3]. MAIC has been successfully used to identify HDF of single virus entities, such as IAV [3] and betacoronaviruses including SARS-CoV-2 [14], and has also been applied in meta-analyses of multiple pooled CRISPR screens for identifying HDF of IAV [15]. We build on MAIC as an evidence-harmonization layer, but go beyond aggregation by training machine learning models to learn transferable patterns of HDF from multi-scale gene and network features—thereby aiming to improve prioritization across viruses and experimental contexts. We computed a comprehensive set of features for each host gene, including sequence descriptors, Gene Ontology based gene set and pathway enrichment scores, protein domains, evolutionary conservation metrics, protein interaction topology, node embeddings, and subcellular localization probabilities. These features were utilized to train heterogeneous graph attention networks alongside classical machine learning models. To bridge computational prioritization with translational utility, we report (i) a conserved set of 118 HDF shared across all four viruses and (ii) a broader core of 427 genes shared by at least three viruses, and we map prioritized genes to host processes and viral life-cycle stages to support testable hypotheses for follow-up perturbation experiments. Finally, we demonstrate that the pan-viral signature generalizes beyond the four viruses to EBOV as a representative filovirus.

## Methods

### Overall description of the workflow

**Fig. 1:**
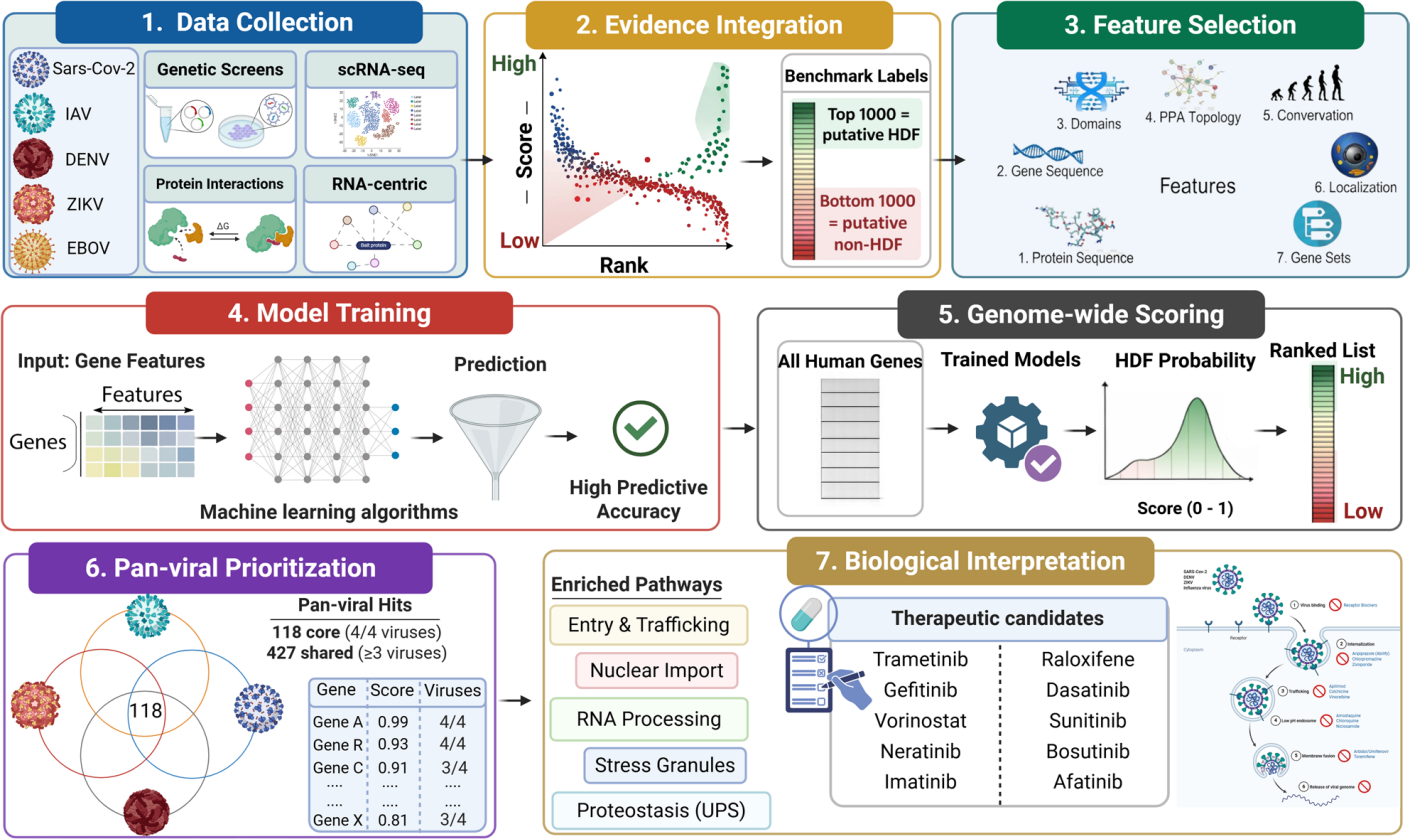
Simplified overview of the pan-viral evidence-to-prioritization workflow. The figure highlights the “journey” of the data from public multi-omics evidence to prioritized biological targets. First, heterogeneous host-factor evidence was assembled for each virus from perturbation screens, virus–host interaction datasets, single-cell transcriptomes, and RNA-centric studies. Second, for each virus, these datasets were integrated using MAIC to obtain an evidence-weighted ranking and to define benchmarks. Third, informative gene- and network-level features (variables) were computed and used to train machine-learning models. Fourth, the trained models were applied to all human genes to generate HDF probabilities and ranked candidate lists. Finally, cross-virus comparison identified shared pan-viral candidates, which were interpreted through enriched pathways of the host cells, viral life-cycle stages, and therapeutic opportunities. The biological signals informing prioritization include, for example, network centrality and trafficking- or organelle-related features for SARS-CoV-2, and closeness, endosomal acidification, spliceosome-associated RNA processing, and COPI-linked trafficking for IAV. For flaviviruses, we observed endocytosis, ER protein processing/glycosylation, stress-granule biology, and nuclear transport/RNA-processing programs as key host processes consistent with the model outputs (see results)

Figure 1 provides a schematic overview of the study workflow, emphasizing the “data journey” from public resources to prioritized cellular targets. Briefly, we performed a comprehensive systematic review to assemble virus-specific host-factor evidence from complementary modalities, including CRISPR/Cas9 and RNAi knockout/down screens, single-cell transcriptomic datasets, and viral protein/RNA–host protein interaction studies. To reconcile limited concordance across individual studies and to leverage orthogonal sources, we combined all gene lists using MAIC, for each virus separately. MAIC computed an overall ranking for all genes. We used the extremes of this ranking to define putative benchmark labels for model training and evaluation (top 1000 genes as HDF and bottom 1000 genes as non-HDF per virus). Genes outside these extremes were not used for model fitting or benchmarking, but were retained for genome-wide scoring in downstream analyses. Subsequently, we trained two complementary machine learning model families in parallel: (i) classical tree-based models, including Random Forest (RF) [17] and Extreme Gradient Boosting (XGBoost) [18], and (ii) a Heterogeneous Graph Attention Network (HAN). Where indicated (HAN + RF), we combined predictions from RF and HAN in an ensemble to improve robustness. XGBoost was not pursued in the ensemble because it was not superior to RF. For a more detailed, method-centric workflow (including modeling components), see Figure S2 in the Supplementary Material.

### Construction of the benchmark labels

We performed a PRISMA-guided literature review and curated virus-specific datasets, which we organized into four evidence categories: Genetic (CRISPR-Cas9 knock-out and/or RNAi knock-down), PPI (experimentally determined virus–host protein–protein interactions), transcriptome (single-cell), and RNA-centric (vRNA–host protein or vRNA–host RNA interactomes) (Fig. S1). The full search strings (databases, exact keywords, operators) are provided in Supplementary Text S3. We selected the following numbers of datasets, for SARS-CoV-2: 27 genetic, 11 PPI, two transcriptome, two RNA-centric (in total 42); for IAV: 18 genetic, 16 PPI, one transcriptome, zero RNA-centric (in total 35); for DENV: 14 genetic, 16 PPI, one transcriptome, four RNA-centric (in total 35); for ZIKV: eight genetic, nine PPI, one transcriptome, three RNA-centric (in total 21); for EBOV: seven genetic, four PPI, zero transcriptome, two RNA-centric (in total 13). The numbers are listed in Table S1, more details about the original publications are given in Tables S2–S6, in which each study is listed as ranked (the original publication supplied an explicit rank/score for each gene) or unranked (unranked lists of genes). The full breakdown appears alongside each entry in Tables S2–S6.

### Preprocessing of the data

**CRISPR/Cas9 and RNAi screens**: Gene lists from both methodologies were extracted from the according publication. Screens were considered as ranked (based on statistical metrics such as *p*-value or adjusted *p*-value when available) if it was documented that the gene lists had been analyzed using statistical tools such as MAGeCK (Model-based Analysis of Genome-wide CRISPR/Cas9 Knockout) [19], RIGER (RNAi Gene Enrichment Ranking) [20], RSA (Redundant siRNA Activity) [21]; otherwise, they were classified as unranked.

**scRNA-seq data**: All single-cell datasets were downloaded from Gene Expression Omnibus (http://www.ncbi.nlm.nih.gov/geo/) and processed separately for each study using a common Seurat workflow [22]. After sample-level quality control, cells with extremely low library size or gene complexity and cells with high mitochondrial read fractions were removed (typically, cells with <200 detected genes, >6000 detected genes, or >20% mitochondrial UMIs were filtered out). Gene-level counts were then normalised, log-transformed, and scaled. Depending on sequencing depth and heterogeneity, we used either Seurat’s standard “LogNormalize” pipeline or SCTransform. For datasets with moderate depth and limited batch structure we applied LogNormalize; for larger or more heterogeneous datasets we used SCTransform to better stabilise the mean–variance relationship. After normalisation, we performed principal component analysis (PCA) [23] on the host-gene expression matrix, and used these PC both for clustering and for visualisation by Uniform Manifold Approximation and Projection (UMAP) [24]. Seurat’s FindNeighbors/FindClusters functions with the Louvain algorithm (resolution ~0.4–0.8) were used to define major cell clusters. UMAP embeddings were inspected to confirm cluster structure and to detect potential batch effects. When a batch structure within a dataset was present, we applied integration using Seurat/Harmony before downstream analysis. Datasets from different studies were not merged, each study was processed and analysed independently, contributing its own ranked gene list to MAIC.

Viral transcripts were quantified per cell by summing raw counts across all viral genes for that dataset. We then defined a per-cell viral-load score

1 $$vloa{d_c}{\rm{}} = {\rm{lo}}{{\rm{g}}_2}\left( {viralcount{s_c} + 1} \right),$$

where *viral counts*_*c*_ denotes the total number of reads mapped to the viral genome in cell c. The value *vload*_*c*_ was used only as a trait in correlation analyses. Viral genes were excluded from PCA, clustering, and network construction. Genes expressed in fewer than 5% of cells in a dataset were removed prior to network analysis.

To relate host-gene expression to viral load we applied high-dimensional weighted gene co-expression network analysis (hdWGCNA) [25] on each scRNA-seq dataset separately. Within each major Seurat cluster, we first constructed metacells by aggregating groups of k = 25 nearest-neighbour cells (max_shared = 10 cells shared between any two metacells), which reduces sparsity while preserving local structure. A signed co-expression network was then built using Pearson correlations between metacell-averaged expression profiles, transformed into an adjacency matrix with a soft-threshold power β chosen by the scale-free topology criterion, as implemented in hdWGCNA. Topological overlap was used as a distance measure, and genes were hierarchically clustered. Modules of tightly co-expressed genes were identified by dynamic tree cutting and merged using default hdWGCNA parameters (merge height ≈0.25). For each module, hdWGCNA computed the first principal component of the module’s expression matrix denoted as the module’s eigengene (ME), serving as a low-dimensional summary of the module. Module–trait associations were assessed by first correlating the eigengene values with the viral load of each metacell leading to a correlation coefficient r_m_ for each module. Only modules with significant correlation were used, i.e., having a false discovery rate ≤ 0.05, r_m_ was set to zero otherwise. Similarly, the correlation of gene g in module m with the module eigengene EM_m_ denoted as kME_m_(g) was computed as the Pearson correlation between the expression vector of gene g across metacells, and the EM_m_ across those same metacells. To obtain gene-level scores usable for ranking, each gene *g* in module m received score

2 $$s_g^{\rm{m}} = {r_m} \times z\left( {{\rm{kM}}{{\rm{E}}_m}\left( g \right)} \right),$$

where z(.) denotes within-module z-score normalization of kME_m_. If a gene belonged to multiple significant modules, the value with the largest absolute magnitude was used. Scores were min–max rescaled to [0,1] per dataset and used as the scRNA-seq evidence for MAIC. Genes from non-significant modules received the value zero. For viruses represented by multiple scRNA-seq datasets, each dataset contributed an independent ranked list to MAIC. Viruses without single-cell data (e.g., EBOV) simply lacked this evidence channel.

**PPI and RNA-protein interaction data**: We collected and extracted data from studies that experimentally screened human protein-viral protein/RNA interactions. For the PPI category, the reported proteins were categorized as ranked if they were accompanied by metrics such as spectral count reproducibility, signal-to-noise ratio, or similar quantitative measures, as generated by methods like MiST (Mass Spectrometry Interaction Statistics) [26] or SAINT (Significance Analysis of INTeractome) [27]. For RNA-protein interactions, the gene lists were classified as ranked if experimental techniques such as CLIP-Seq, iCLIP, and PAR-CLIP reported their interactions with statistical measures like binding scores or false discovery rates (FDR). Otherwise, the data, such as those derived from RIP-Seq, were categorized as unranked.

All data sets from five particular experimental data types were integrated using the MAIC framework, a method developed for combining ranked and unranked gene lists from heterogeneous sources with unknown quality, which are expected to contain common gene lists or similar rankings of genes. The MAIC algorithm assigns a score to each gene based on its overlap with other lists and, when applicable, its rank within those lists. Common genes reported across different categories are assigned higher scores compared to those appearing repeatedly in gene lists within the same category. In addition, a weighting score is calculated for each gene list by summing the scores of all the genes it contains. This approach ensures that gene lists with a higher proportion of genes supported by evidence from multiple studies receive higher weighting scores. Gene scores are then iteratively recalculated using the updated weighting scores, while the weighting scores are simultaneously recalculated by summing the revised gene scores. This iterative process continues until convergence is achieved. Finally, the algorithm produces a consolidated and ranked list of genes, highlighting those with the strongest evidence across multiple datasets. To obtain sufficiently powered and class-balanced training labels while minimizing class-imbalance bias, we designated the top 1000 ranked genes as putative HDF and the bottom 1000 as non-HDF per virus. The rest of the genes in the list were not used during training and testing the machine learning models, but were used to apply the machines predicting HDF.

### Feature extraction

For each host gene, we compiled a comprehensive feature set informed by prior work on essential-gene prediction [28, 29] and recent studies of host-factor discovery [16, 30]. In total, 60,516 features were computed per gene/protein, spanning (i) intrinsic sequence-derived descriptors (obtained directly from DNA and protein sequences) and (ii) extrinsic biological context (capturing network topology, homology, subcellular localization, and functional domains). We organized these features into six categories and then applied a stringent, multi-stage dimensionality-reduction pipeline to mitigate redundancy and overfitting.

1. Gene and protein sequence descriptors: For each gene, we used seqinR [31] to calculate amino acid composition, physicochemical class frequencies and theoretical isoelectric points. Autocorrelation, Conjoint Triad descriptors, quasi sequence order and pseudo amino acid compositions were derived using protr [32]. CodonW (https://codonw.sourceforge.net/) and rDNAse (https://github.com/wind22zhu/rDNAse) provided gene length, GC content, optimal codon frequencies, auto covariance, pseudo nucleotide composition and k-mer frequencies (2–7 mers) and also calculated sequence attribute distribution features. The sequence attribute distribution encodes seven physico‑chemical attributes—hydrophobicity, normalized van der Waals volume, polarity, polarizability, charge, secondary structure and solvent accessibility—and groups amino acids into polar, neutral or hydrophobic classes. Each feature name encodes the attribute (first digit), class (second digit) and sequence location (last three digits), e.g., seq.attribute.distribution 42100 denotes polarizable, neutral residues located at the end of the sequence.
2. Gene Ontology and pathway enrichment: Gene set features were derived by performing gene set enrichment based on the gene set definitions from Gene Ontology (GO), for each gene’s neighborhood in the human protein–protein interaction (PPI) network. The PPI was assembled from STRING v12 [33]. For every GO term, we calculated the negative log₁₀ of *p*-value obtained from a Fisher’s exact test to quantify enrichment. To reduce redundancy among GO terms, overlapping gene sets were removed by solving a stable set problem (details, see reference [34]). Gene sets from KEGG and Reactome were also included.
3. Protein domains and structural motifs. Using BioMart [35], we enumerated Pfam domains as well as the number of coiled coils, predicted membrane helices, post translational modifications, β turns, cofactor binding sites, acetylation and glycosylation sites, signal peptides, and transmembrane helices. We also included features describing the number and lengths of untranslated regions (UTRs).
4. Evolutionary conservation and homology: Conservation features were generated by aligning each protein against the RefSeq database [36] using PSI BLAST [37] and counting the number of homologs at E-value thresholds ranging from 1e-5 to 1e-100. We also recorded the number of orthologs per gene and calculated K_a_/K_s_ ratios from variome data to capture evolutionary constraints.
5. Topology in protein–protein interaction networks: We employed the Python library NetworkX to compute various network metrics for each gene within the human protein-protein interaction (PPI) network derived from STRING v12. Specifically, we calculated the degree, degree distribution, centrality measures (including closeness and betweenness), clustering coefficient, and PageRank.
6. Node embeddings and subcellular localization: Previous research has demonstrated that the inclusion of virus–host and virus–virus interaction data, such as through interactome maps, network proximity, or diffusion, enhances the prioritization of pertinent host factors and targets [38, 39]. To capture higher-order connectivity patterns, we computed node2vec embeddings [40] separately for the host and viral protein interaction networks. Specifically, we applied node2vec to the human PPI network (STRING v12) to learn a 64-dimensional embedding for each host protein, and similarly applied node2vec to each virus’s own protein–protein interaction (V–V) network to embed its viral proteins. This biased random-walk approach (skip-gram training) produces embeddings that preserve neighborhood relations in each network. We integrated viral protein node embeddings into the HAN model to incorporate virus-specific interaction context.

The inclusion of compartment probabilities aids the model in distinguishing proteins that operate in specific cellular localizations. Hence, subcellular localization features were included, which were derived from DeepLoc [41], which assigns probability scores for 11 compartments (cytoplasm, nucleus, mitochondria, endoplasmic reticulum, Golgi apparatus, lysosome, vacuole and peroxisome, plasma membrane, extracellular and chloroplast). In total 60,516 features were calculated for each gene.

### The cross-validation procedure

For each virus, we trained and evaluated the models using a nested cross-validation (CV) scheme (10-fold outer CV and 5-fold inner CV), as schematized in Fig. S6. In the outer loop, the data were split into 10 folds, in each outer split, one fold (10% of the samples) was held out as an independent test set and the remaining 90% were used exclusively for model training, feature selection, and hyperparameter tuning. Within this 90% training portion, we ran a 5-fold inner CV. All preprocessing steps, including feature selection and correlation-based pruning, were performed within each inner training fold and applied to the corresponding inner validation fold to avoid information leakage.

### Feature selection and modeling strategy

All features were standardized via z-score normalization prior to feature selection. To address the high dimensionality relative to the number of samples, we applied a multi-step feature selection pipeline for each virus-specific dataset. First, we performed recursive feature elimination (RFE) using a Random Forest classifier (scikit-learn implementation), iteratively removing the least important features in each training fold until a smaller subset remained; the target number of features (on the order of a few hundred) was empirically chosen based on inner-fold performance. Next, within each training fold we removed any remaining highly collinear features (Pearson |r| >0.70) to eliminate redundancy, leaving roughly 300–500 features per virus for the classical models (Random Forest and XGBoost). For the graph-based model (heterogeneous graph attention network, HAN), we took a more conservative approach: all sequence-derived descriptors (~37,660 nucleotide/protein sequence features) were excluded upfront, since preliminary RF/XGBoost experiments showed no considerable performance benefit from including them. The same Pearson correlation filter was applied to the remaining features, yielding approximately 1,450 input features per human protein node. All feature selection steps were confined to the inner cross-validation folds to avoid overfitting, substantially reducing the feature-to-sample ratio before modeling.

We evaluated two complementary machine learning approaches on the selected feature sets: (i) classical ensemble classifiers and (ii) a graph-based neural network. The classical models included Random Forest and XGBoost, which served as baseline classifiers using the reduced feature subsets. The graph-based approach utilized HAN to integrate host gene features with virus–host interaction network context. In the HAN architecture, each host protein’s feature vector (~1,450 dimensions after selection) was first projected into a 256-dimensional embedding via a trainable linear layer. Our method then applied graph attention mechanisms over predefined meta-paths connecting human and viral nodes to aggregate neighborhood information. We incorporated regularization in HAN through dropout and employed early stopping based on validation loss to mitigate overfitting. To exploit the complementary strengths of RF and HAN, we implemented an ensemble through stacked generalization. In each outer 10-fold cross-validation split (with 10% of samples held out as test), RF and HAN were trained on the training partition and generated out-of-fold probability predictions for each sample in that partition; we concatenated the two predicted class probabilities (one from each model) as features for a meta-learner (a logistic regression classifier). This meta-classifier was trained on the combined out-of-fold predictions of the training data and then applied to the held-out test fold to produce the final ensemble prediction. Given XGBoost’s consistently lower performance than RF and its lack of incremental benefit in stacking, our final ensemble comprised RF and HAN only.

### Heterogeneous graph construction

For each virus, we built an independent heterogeneous graph with two node types—human proteins (H) and viral proteins (V)—and three edge types: H–H (human–human PPI), H–V (virus–host interactions for the virus under study), and V–V (virus–virus interactions). As our prediction target is defined on human proteins, all meta-paths started and ended at H. In the network, the following meta-paths were established: Φ₁: H→H, Φ₂: H→V→H, Φ₃: H→H→V→H, Φ₄: H→V→V→H.

To control computational cost and prevent dense H–H edges from dominating message passing, we used budgeted fan-out sampling along meta-paths. Fan-out budgets were selected via inner-loop hyperparameter tuning within predefined ranges K_VH ∈ {10,15,25,40}, K_VV ∈ {5,10,20}, and H→H hop budgets for Φ₂/Φ₃ ∈ {(10/5), (20/10), (5/3)}. The final values are listed in Table S14. Budgets were kept comparable across meta-paths so that the semantic-attention module is not biased by raw path frequencies.

### Message passing

#### Node-level attention

Since nodes were heterogeneous, different types of nodes belonged to different feature spaces. Hence, we projected the features of different nodes into a common feature space using a feature transformation matrix *M*_Φ_ specific to each meta-path type Φ. For a node *i*, this projection is given by

3 $$h_i^\prime = {M_{\rm{\Phi }}} \cdot {h_i},$$

in which *h*_*i*_ and *h’*_*i*_^′^ are the original and projected features of node *i*, respectively. Next, node-level attention weights were learned for different types of nodes on the same meta-path. For node pairs (*i*,*j*) that were connected via a meta-path, the asymmetric importance $e_{ij}^{\rm{\Phi }}$ of node *j* to node *i* under meta-path type Φ was calculated as

4 $$e_{ij}^{\rm{\Phi }} = {\rm{at}}{{\rm{t}}_{{\rm{node}}}}\left( {h_i^\prime,h_j^\prime;{\rm{\Phi }}} \right)$$

Here, att_node_ (⋅) denotes a single-layer neural network that produces unnormalized node attention coefficients $e_{ij}^{\rm{\Phi }}$ for a fixed target node *i*, given the projected embeddings *h’*_*i*_^′^ and *h’*_*j*_ on meta-path type Φ. As implied by the formulation, the weight for a node pair *(i,j)* depended on the projected embedding vectors *h*′_*i*_ and *h*′_*j*_, and the weight coefficients for all neighbors of node *i* were then obtained by a SoftMax normalization:

5 $$\begin{aligned} \alpha _{ij}^\Phi = {\mathrm{softmax}}\left( {e_{ij}^\Phi } \right) = & \frac{{\exp \left( {e_{ij}^\Phi } \right)}}{{\sum\nolimits_{l \in N_i^\Phi } {\exp } \left( {e_{il}^\Phi } \right)}} \\ = & \frac{{\exp \left( {\sigma \left( {a_\Phi ^T \cdot \left[ {{{h'}_i}||{{h'}_j}} \right]} \right)} \right)}}{{\sum\nolimits_{l \in N_i^\Phi } {{\mathrm{exp}}} \left( {\sigma \left( {a_\Phi ^T \cdot \left[ {{{h'}_i}||{{h'}_l}} \right]} \right)} \right)}} \\ \end{aligned} $$

For a given meta-path ${\rm{\Phi }}$, let $N_i^{\rm{\Phi }}$ be the set of ${\rm{\Phi }}$-neighbors of node *i.* In this formula, $\sigma$ is the activation function, ${a_{\rm{\Phi }}}$ represents the node-level attention vector for meta-path ${\rm{\Phi }}$, || represents the concatenation operation. The node-level embedding $Z_i^{\rm{\Phi }}$ that aggregated information from the projected features of *i* and all its ${\rm{\Phi }}$-neighbors was calculated by

6 $$Z_i^{\rm{\Phi }} = \sigma \left( {\mathop \sum \limits_{j \in N_i^{\rm{\Phi }}} \alpha _{ij}^{\rm{\Phi }} \cdot h_j^\prime} \right).$$

Given a set of meta-path types {Φ_1_, Φ_2_, …., Φ_p_} (with *p* = 4 in our setting, corresponding to the four meta-path types Φ_1_–Φ_4_ defined above), applying the above procedure to each meta-path type produced *P* groups of semantic-specific embeddings {$Z_0^{\rm{\Phi }}$, $Z_1^{\rm{\Phi }}$, …, $Z_p^{\rm{\Phi }}$}, which could later be fused by the semantic-level attention module.

#### Semantic-level attention

After computing embedding feature $Z_i^{\rm{\Phi }}$ for node *i*, we let the model learn how much each meta-path should influence the final embedding of *i*. Concretely, each $Z_i^{\rm{\Phi }}$ was scored by a small feed-forward function and mapped to a non-negative importance weight with a softmax across meta-path types,

7 $${\omega _{{{\rm{\Phi }}_i}}} = {1 \over {\left| V \right|}}\left( {\mathop \sum \limits_{i \in V} {q^T} \cdot {\rm{tanh}}\left( {W \cdot z_i^{\rm{\Phi }} + b} \right)} \right),$$

8 $${\beta _{{{\rm{\Phi }}_i}}} = {{\exp \left( {{\omega _{{{\rm{\Phi }}_i}}}} \right)} \over {\mathop \sum \nolimits_{i = 1}^p {\rm{exp}}\left( {{\omega _{{{\rm{\Phi }}_i}}}} \right)}},$$

where *V* is the node set, *W* is the learnable weight matrix, *b* represents the bias vector, and q was a trainable context vector that lets the model express which semantics are globally informative.

The fused node feature was computed by aggregating semantic features from different types of meta-paths, as follows:

9 $${Z_i} = \mathop \sum \limits_{j = 1}^P {\beta _{{{\rm{\Phi }}_j}}} \cdot Z_i^{{{\rm{\Phi }}_j}},$$

where *P* is the number of meta-path types and ${\beta _{{{\rm{\Phi }}_j}}}$ were semantic-level attention weights normalized by softmax.

#### Feature fusion and prediction

The multihead attention mechanism combined the outputs of multiple attentions in different subspaces, allowing the model to emphasize informative components and capture cross-feature interactions. Let $L \in {^{n \times d}}$ denote the stacked input features for multihead attention. With attention head $a \in \left\{ {1, \ldots,h} \right\}$, we formed queries, keys, and values via learned linear projections:

10 $${{\rm{Q}}_a} = LW_a^{\rm{Q}},$$

11 $${K_a} = LW_a^K,$$

12 $${V_a} = LW_a^V,$$

where the weight matrices for head $a$ were represented by$W_a^Q,W_a^K \in {R^{d \times {d_k}}}$ and $W_a^V \in {R^{d \times {d_v}}}$, with ${d_k}$ = ${d_v}$= ${d \over h}$. The output of the attention mechanism for head *a* was then:

13 $${H_a} = {\rm{Attention}}\left( {{Q_a},{K_a},{V_a}} \right) = {\rm{softmax}}\left( {{{{Q_a}K_a^T} \over {\sqrt {{d_k}} }}} \right){V_a}.$$

Outputs from all heads were concatenated to obtain the final output of the multihead attention:

14 $$R = {\rm{Concat}}\left( {{H_1}, \ldots,{H_h}} \right).$$

Here, *R* denotes the final node representation matrix with one row per node. LayerNorm is applied along the feature dimension, and the Linear layer maps each *d*-dimensional node representation to C logits. In our case, *c* = 2, corresponding to the two classes HDF and non-HDF. The Softmax is applied row-wise over the class dimension, yielding a normalized probability distribution over the two classes for each node.

15 $$y\prime = {\rm{Softmax}}\left( {{\rm{Linear}}\left( {{\rm{LayerNorm}}\left( R \right)} \right)} \right).$$

Here $y\prime$ contained the predicted class probabilities and the predicted label was:

16 $$y = {\rm{argmax}}\left( {y\prime} \right).$$

y was used to minimize the cross-entropy (BCE) loss.

#### Hyperparameter tuning

Hyperparameter tuning was conducted using an inner-loop cross-validation on training and validation folds within a 10-fold cross-validation. Performance was evaluated by the area under the ROC curve (AUROC) and the area under the precision–recall curve (AUPRC) (Table S14). The tuning process systematically explored model capacity parameters (hidden dimensions of 128, 256, or 512; attention heads of 2, 4, or 8; dropout rates of 0.2, 0.3, or 0.5) and optimization settings (Adam optimizer with learning rates of 5e-4, 1e-3, or 2e-3 and weight decay of 1e-5, 1e-4, or 5e-4). Meta-path neighbor budgets were varied by adjusting K_VH (virus–host neighbors: 10, 15, 25, or 40), K_VV (virus–virus neighbors: 5, 10, or 20), and the host–host hop limits for meta-paths φ₂ and φ₃ (5 and 3, 10 and 5, or 20 and 10 hops, respectively). Additionally, multi-head attention (MHA) fusion was toggled on or off, and a fixed DropEdge probability of 0.20 was applied to φ edges. Within each virus and outer cross-validation fold, we used an inner-loop random search over predefined discrete hyperparameter grids rather than an exhaustive grid search.

To establish strong non-neural network baselines, we optimized Random Forest and XGBoost parameters. For the RF tuning process, the parameters n_estimators, max_depth, min_samples_leaf, and min_samples_split were optimized. In the case of XGBoost tuning, the parameters n_estimators, max_depth, colsample_bytree, learning_rate were optimized within a tuning grid, while the gamma and subsample parameters were held constant at 0 and 1, respectively.

## Statistical analysis and implementation

All statistical analyses were performed in R (version 4.4.0) and Python (version 3.10.13) on Linux and Windows workstations. Gene set enrichment analyses were carried out using the gprofiler2 R package [42], Reactome and KEGG as annotation sources, applying a hypergeometric test with Benjamini–Hochberg false discovery rate correction. GO term redundancy was removed as described above (Feature Extraction), and related GO terms were grouped into 18 curated meta-categories using Slimformer [43].

## Results

### Systematic review of the literature

**Fig. 2:**
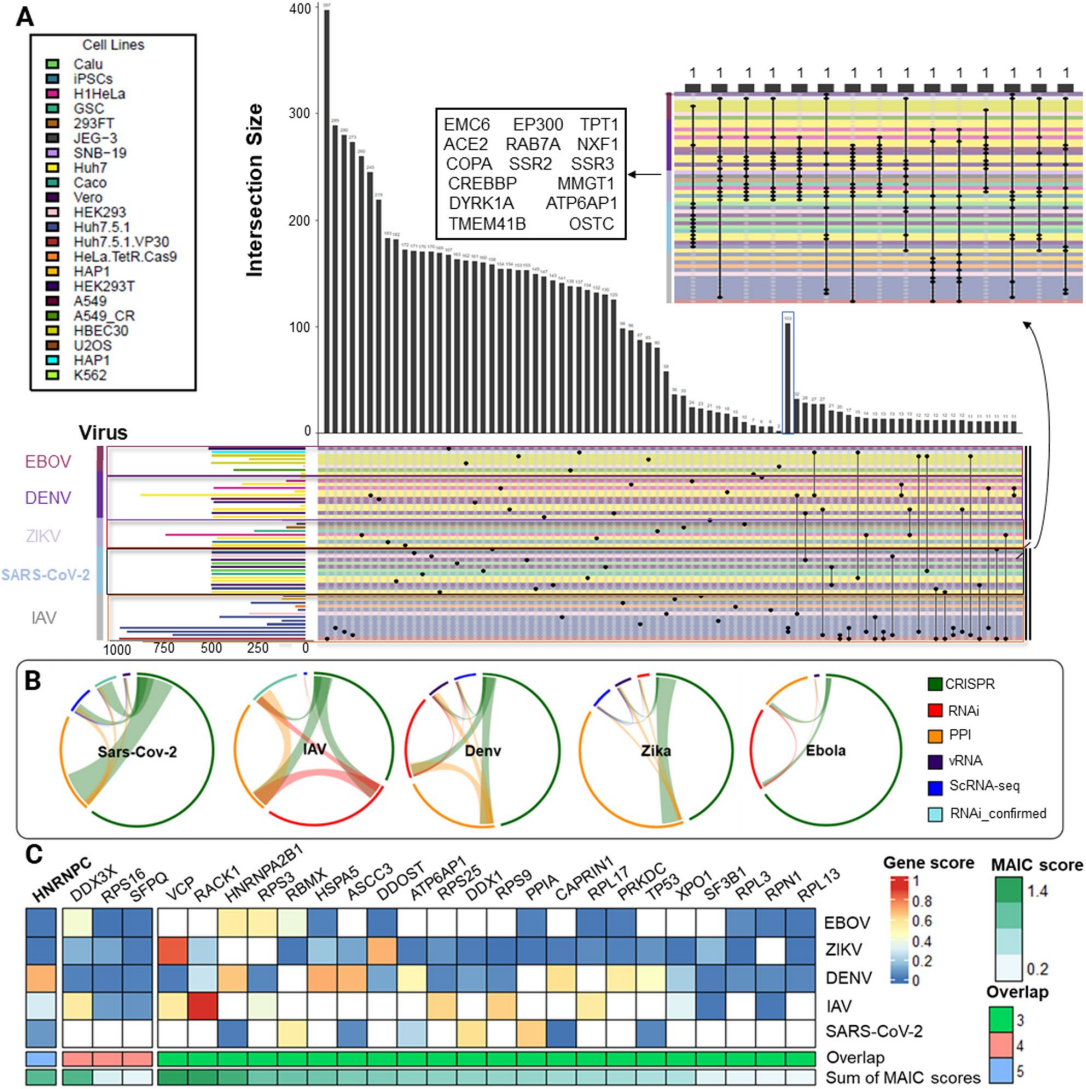
Overview of the overlaps of the different datasets explored in this study. **A**. The UpSet plot shows the overlaps of top ranked hits from genetic perturbation screens (CRISPR knock-out; RNAi knock-down) across the five investigated viruses. Vertical bars show intersection sizes; horizontal bars show per-virus sizes of the hit lists for different data sources. For reading the specific data sources, we refer to additional file *1*. Rows are denoted according to the first author of the publication. Row colors correspond to the used cell line, listed in the upper left box. The single large pairwise peak (marked by a blue box) arises from one IAV study profiling two strains showing an unusual high overlap suggesting dependency of those lists. **B**. Modality chords for each virus summarize overlaps among evidence categories (CRISPR based gene knockout, RNAi based gene knockdown, PPI: virus - host protein-protein interaction, vRNA: viral RNA - host protein interactions, ScRNA-seq: single cell transcriptomics, RNAi_confirmed: subset of RNAi-derived hits that were validated in follow-up assays in the original studies). The link width is proportional to the number of shared genes. The modality chords visualize the pronounced heterogeneity in data availability across viruses. **C**. Heat map for 26 genes which highly ranked for at least 3 out of 5 viruses of the virus specific benchmarking datasets; color code: red (blue) if high (low) ranking in the top 150 ranking genes, white: not among the top 150 ranking genes for the according virus. The overlap across the benchmarks remains low after integration: hnRNPC appears in the top 150 genes of all five viruses, RPS16, SFPQ, and DDX3X occur in four. Genes are ordered by the overlaps followed by the sum of MAIC scores across viruses

We conducted a systematic literature review in accordance with the Preferred Reporting Items for Systematic Reviews and Meta-Analyses (PRISMA) guidelines to aggregate published screening-based and other experimental evidence on HDF for the viruses under investigation (Fig. S1). The search yielded 884 unique database records (deduplicated citations screened at the title/abstract level), of which 115 full-text reports met the eligibility criteria. From these reports we extracted 146 distinct HDF gene lists, 42 for SARS-CoV-2, 35 for IAV, 35 for DENV, 21 for ZIKV, and 13 for EBOV. For each study, we documented whether the list was ranked or unranked, and annotated its experimental modality: CRISPR-based knockout, RNAi knockdown, virus–host protein–protein/RNA–protein interactomes, or single-cell RNA sequencing. The number of datasets ultimately used per virus and modality is given in Table S1, Tables S2–S6 list for each used data source their modalities and references. Next, we analyzed the overlaps of listed HDF among the different datasets. Across studies, the overlap was sparse and uneven. Intersections among screens were typically small even within the same virus. The largest pairwise overlap (103 genes) arose from two HDF lists reported by the same study (two IAV strains), indicating non-independence between those lists (Fig. 2A). Aside from this, we observed striking heterogeneity in the data. A modality-level view (Fig. 2B) reveals additional imbalance: SARS-CoV-2 and ZIKV were dominated by CRISPR-based knockout and PPI datasets, whereas IAV, DENV, and EBOV comprised proportionally more knockdown datasets. To leverage consistency in the data in an automated way, we sought applying machine learning. For the machines, a benchmarking dataset needed to be assembled based on these different data entities. However, the heterogeneity in the data sources cautions against naïve vote-counting methods for data integration, and rather motivate considering the sources in the statistical model when combing the data. For this, Li and coworkers developed Meta-Analysis by Information Content (MAIC), a data-driven rank-aggregation framework that integrates heterogeneous gene lists by assigning a higher weight to datasets whose hits are corroborated by other studies, and that accommodates both ranked and unranked lists [3]. Accordingly, we employed MAIC to integrate datasets based on diverse experimental sources, encompassing both ranked and unranked lists. Despite the limited gene-level concordance observed, the application of MAIC facilitated the identification of a conserved host signal. When applied separately to the various viruses under analysis, an overlap of 26 genes was identified within the top 150 ranked genes for at least three viruses. Notably, Heterogeneous Nuclear Ribonucleoprotein C (hnRNPC) was the sole gene consistently ranked among the top 150 genes across all five viruses (Fig. 2C). To assess whether this overlap arose by chance, we performed an empirical permutation test based on the MAIC gene universe, repeatedly sampling random top-150 gene sets per virus. Across 10,000 permutations, the probability of observing a 5-virus overlap ≥ 1 and a ≥ 3-virus overlap ≥ 26 was *p* ≤ 1.0 × 10^− 4^ for both, indicating that the observed cross-virus consistency is highly unlikely to occur by chance. HnRNPC encodes an RNA-binding protein that plays a role in pre-mRNA processing and RNA metabolism. Recent studies suggest that hnRNPC may enhance viral replication by suppressing type I interferon production through the degradation of the adaptor MITA/STING, thereby potentially attenuating antiviral responses [44]. Beyond hnRNPC, several high-ranking shared candidates have substantial prior support. DDX3X, a DEAD-box RNA helicase, is a well-known host factor. It coordinates innate antiviral defenses and is targeted by viral antagonists across multiple RNA viruses, including IAV. Functional studies and reviews consistently implicate DDX3X in IFN signaling and viral replication control [45, 46]. SFPQ (PSF), a member of the DBHS-family RNA-binding proteins and a component of paraspeckles, plays a crucial role in IAV transcription by facilitating the polyadenylation of viral mRNA. Additionally, during SARS-CoV-2 infection, it binds to the viral RNA genome to enhance viral RNA amplification, aligning with its consistent prioritization across various viruses in this study [47, 48].

The integration of the data led to better consistency, but still asks for improvement. Hence, we now applied two different machine learning strategies trained and validated with these benchmarking datasets, i.e., classical Random Forests (RF) and heterogeneous graph attention networks (HAN). The latter of which was used due to their capability to embed molecular network structures in the search space which may support identifying pathway structures employed by the virus.

### Machine learning models show high prediction performance

**Fig. 3:**
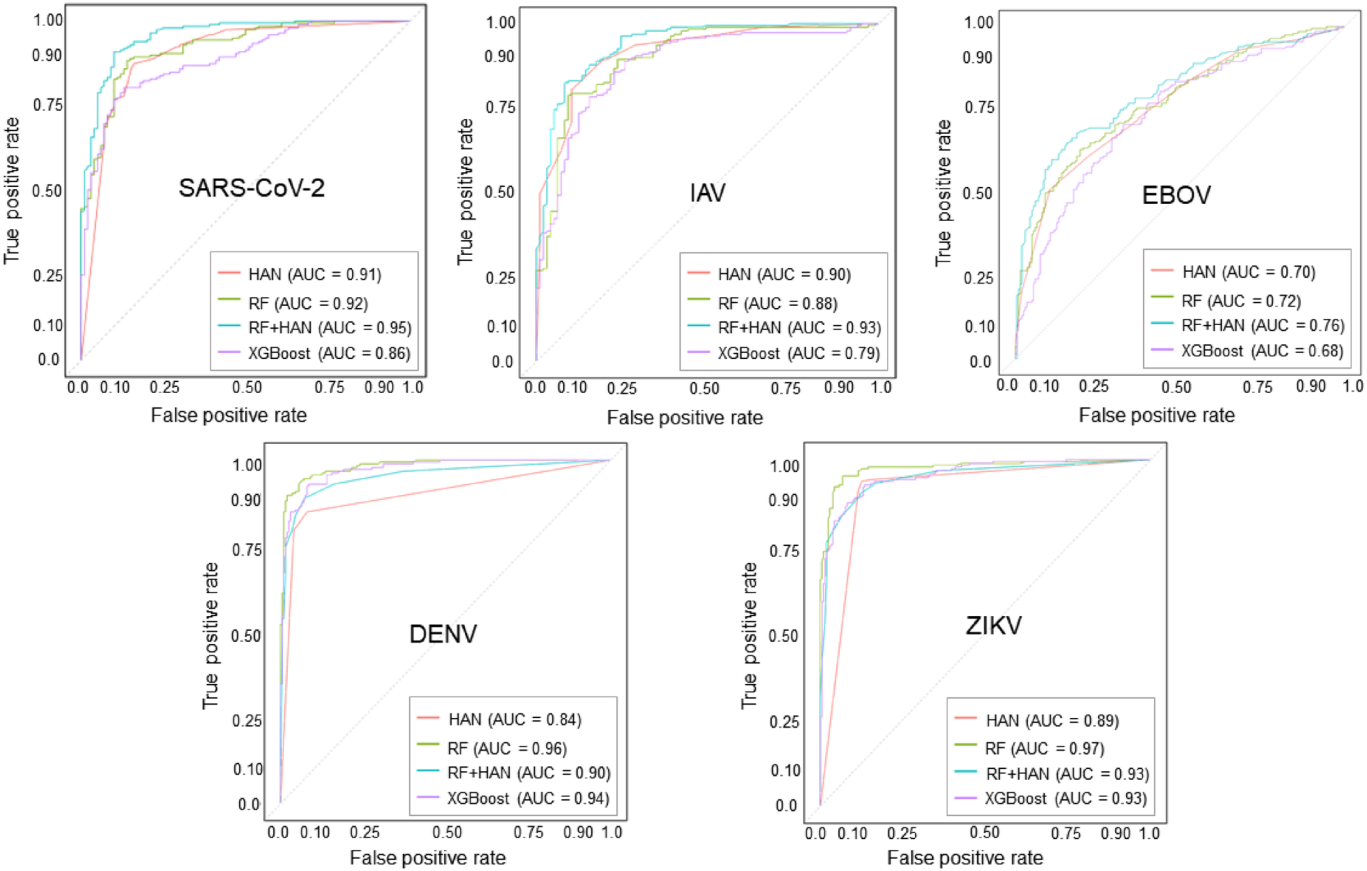
Model performances across viruses. Receiver operating characteristic (ROC) curves for four classifiers—HAN, RF, RF+ HAN (ensemble), and XGBoost

First, we evaluated the model performances on the held-out test sets. We used a leakage-safe nested cross-validation, an outer 10-fold split (10% test per fold) and an inner five-fold cross-validation on the remaining 90% for feature selection, and hyperparameter tuning. Four different machine learning strategies were compared: Random Forests (RF), Heterogenous Graph Attention Networks (HAN), an ensemble of RF and HAN (RF+HAN), and Extreme Gradient Boosting (XGBoost). Figure 3 shows the results. Across the viruses SARS-CoV-2, IAV, ZIKV, and DENV, RF+HAN achieved consistently high discrimination performance (area under the receiver operating characteristic curve, AUROC > 0.90). For SARS-CoV-2 and IAV, RF+HAN showed the best results, RF yielded the best performances for ZIKV and DENV, marginally exceeding HAN and RF+HAN. In contrast, the performances for EBOV were distinctively lower. The best performance for EBOV was achieved by RF+HAN with an AUROC of 0.76. This lower performance may reflect (i) the absence of single-cell transcriptomic data, (ii) fewer datasets in key categories (e.g., protein–protein interaction and CRISPR screens), and (iii) limited gene overlap across datasets, which reduced the cross-study power. Table S7 lists further performance measures. To dissect which evidence sources and feature groups drove these performance gains, we performed systematic ablation studies of the MAIC benchmarks and model features, confirming that the different information channels provide largely non-redundant signals (Supplementary Text S1, Fig. S3 and Table S8).

To obtain these excellent performances, we employed parameter optimization within inner cross-validation loops between training and validation data. The details are given in Methods, here we summarize the most relevant observations from this optimization. For SARS-CoV-2 and IAV, larger hidden dimensions of the HAN embedding space (d = 256) and higher virus–host neighbor budgets (K_VH = 25, defined as the maximum number of virus–host neighbors sampled per viral node in V→H steps) yielded the best performance, consistent with the larger number of available virus–host interactions for these viruses. In contrast, for ZIKV and DENV, smaller hidden dimensions (d = 128) and lower virus–host neighbor budgets (K_VH = 15) were sufficient, suggesting that very large neighbor budgets introduce noise when the virus–host interaction network is sparser. EBOV models favored shallower architectures (d = 128) and had multi-head attention fusion disabled, in line with the limited amount of interaction and label data. These virus-specific selections highlight that model capacity and meta-path neighbor budgets need to be matched to data density and heterogeneity.

### Comparing HDF across viruses shows leveraged consistency in the machine learning based predictions

**Fig. 4:**
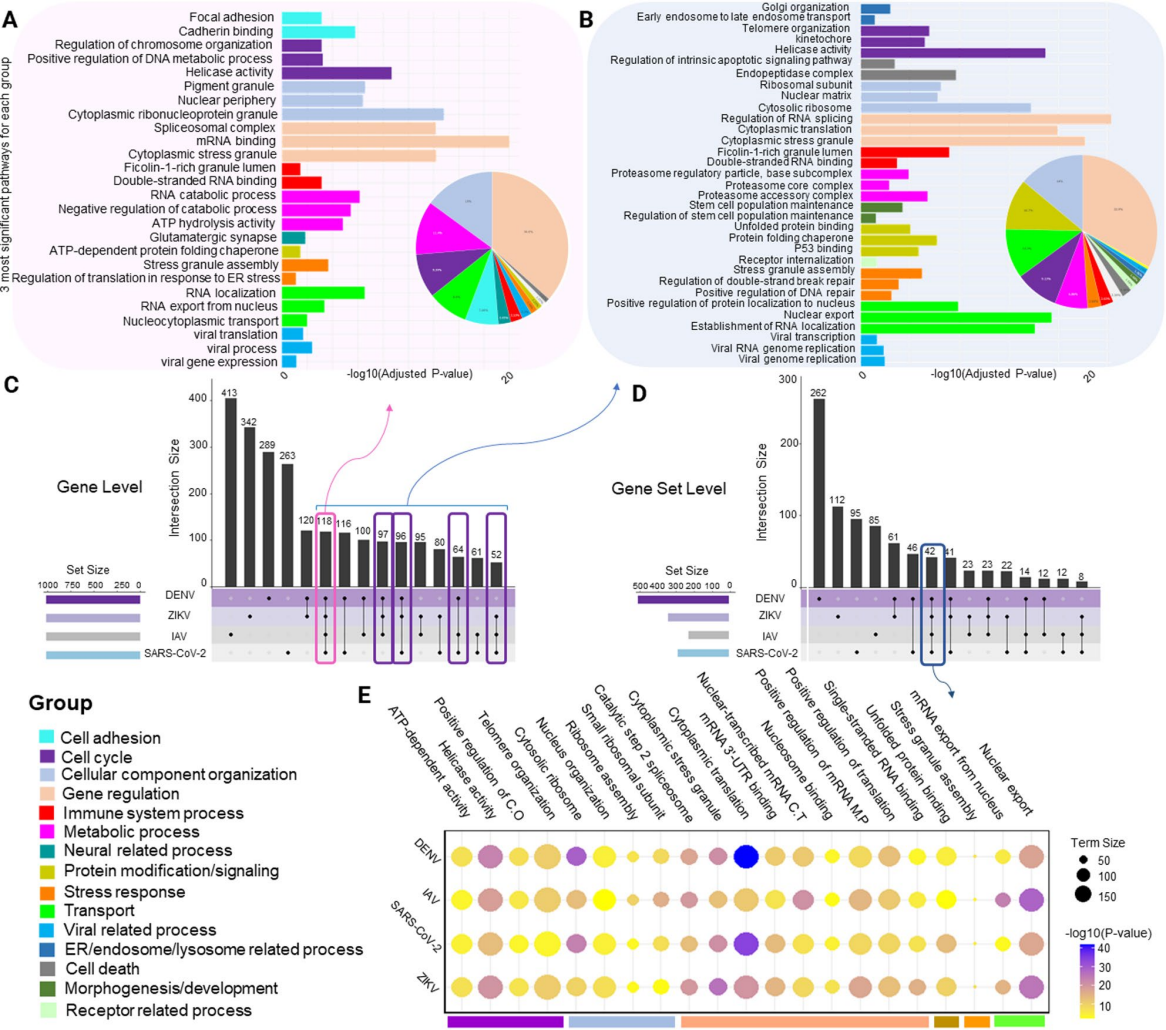
Cross-virus overlap of the top 1000 predicted HDF and their enriched gene sets. **A**, **B**. Functional enrichment of the 118-consensus genes and the 427-core genes. To provide an overview, each gene set was assigned to one of 18 major gene-set categories (color code shown at the bottom left). For each category, the top three significantly enriched gene sets are shown, bar length indicates − log_10_ adjusted *p*-value. **C**, **D**. UpSet plots at the gene (**C**) and gene-set (**D**) level. In total, 118 genes (“118-consensus genes”, pink box) and 42 gene sets were common among all four viruses, and 427 genes were identified in at least three out of four viruses (pink + purple boxes). **E**. bubble plot of the top 20 gene sets among the 42 pathways that were significantly enriched for all four viruses; colors encode significance (−log_10_ adjusted *p*-value), while bubble size reflects the term size

Given that the EBOV model demonstrated inferior performance compared to the others, we focused the pan-viral analyses on SARS-CoV-2, IAV, ZIKV, and DENV. For each virus, we applied the corresponding best classifier (HAN+RF for IAV, and SARS-CoV-2; RF for DENV, and ZIKV) to all human protein-coding genes, ranked them by predicted HDF probability, and retained the top 1000 genes for further analysis. The intersections among these four top-1000 prediction sets yielded 118 genes shared by all four viruses (hereafter referred to as the 118-consensus-genes) and 427 genes present in at least three of the four viruses (the 427-core-genes) (Fig. 4C). To quantify how our integration pipeline improves cross-virus consistency, we compared overlaps at three levels: (i) raw experimental hit lists, (ii) MAIC top-1000 genes per virus, and (iii) top-1000 predictions from machine learning, using a common background of 19,610 protein-coding genes. On average, the experimental hit lists shared only about 13.5% of their top-1000 genes between any two viruses. After MAIC integration, the mean pairwise overlap nearly doubled to 25.6% (significance of the difference: mean −log₁₀*P* = 148.54 across pairs), and the machine learning models further increased the concordance to 40% of hits in common between viruses (mean −log₁₀*P* = 278.06) (Table S9). To go beyond pairwise comparisons, we next quantified multi-virus overlaps by counting, for each level, how many genes were selected in the top-1000 lists of all four viruses and how many occurred in at least three of the four lists. The MAIC top-1000 sets contained 74 genes shared by all four viruses and 337 genes present in at least three viruses, both highly unlikely under a permutation null model (*p* < 10^− 4^). The machine learning based sets expanded this pan-viral core to 118 four-virus genes and 427 ≥ 3-virus genes (the 118-consensus-genes and 427-core-genes) (*p* < 10^− 4^). Together, these results indicate that MAIC already concentrates shared host signals across viruses, and that machine learning further sharpened and enlarged this pan-viral consensus pattern. To facilitate exploration of these pan-viral HDF, we provide an interactive Shiny web application that allows users to browse gene-wise ML scores, MAIC evidence, virus-specific predictions, and functional enrichment results across all four viruses. An interactive Shiny web application for exploring the pan-viral HDF is provided at https://1uzj53-mohadese-naseri.shinyapps.io/panviral_hdf_shiny_app/. Among the 118 consensus genes predicted as HDF in all four viruses, we compiled a manually curated shortlist of the twenty most useful candidates for initial laboratory follow-up (Table 1). The selection was guided by biological interpretability, representation of major viral life-cycle processes, and tractability for perturbation-based validation. The full list of all 118 consensus genes is provided in Table S11.

**Table 1:** Manually curated shortlist from the 118 pan-viral consensus HDFs for initial laboratory validation

Gene	Primary cellular function	Rationale for prioritization	Suggested first-pass validation	Ref.
AP2B1	AP‑2 adaptor complex β subunit; initiates clathrin-mediated endocytosis (CME)	**Virus-specific evidence**: AP2B1/AP2 recruitment is implicated in IAV internalization/entry processes. **Cross-virus relevance:** CME is a common uptake route for multiple enveloped RNA viruses, making AP2B1 a mechanistically interpretable entry candidate.	Models: A549 (IAV), Calu‑3 or A549‑ACE2 (SARS‑CoV‑2), Huh7 (DENV/ZIKV). Perturbation: CRISPRi or siRNA (partial KD). Readouts: early entry (2–6 h antigen/RT‑qPCR), 24–48 h FFU/plaque; viability	[49], [50]
CLTC	Clathrin heavy chain; scaffold for clathrin-coated pits/vesicles	DENV: CLTC knockdown inhibits infectious entry across DENV serotypes in human cells. Cross-virus relevance: IAV entry commonly employs the endocytic machinery and was reported to recruit clathrin components, supporting a shared entry/uptake dependency	Models: Huh7 or HepG2 (DENV), A549/MDCK (IAV), Calu‑3 (SARS‑CoV‑2). Perturb: siRNA/CRISPRi. Readouts: entry/internalization assays (early antigen), RT‑qPCR, FFU/plaque; optional CME inhibitor control	[51], [49]
RAB5	Small GTPase controlling early endosome biogenesis and endocytic trafficking	DENV: Rab5 is required for DENV infectious entry/trafficking. SARS‑CoV‑2: RAB5+ membranes support SARS‑CoV‑2 replication organelle generation (with COPB1), linking entry/early endosomes to replication membrane supply	Models: Huh7 (DENV), Calu‑3 (SARS‑CoV‑2), A549 (IAV). Perturb: CRISPRi/siRNA; optional dominant‑negative Rab5A. Readouts: early endosome markers (EEA1), dsRNA/RO markers, RT‑qPCR, FFU/plaque.	[52, 53]
ATP6AP1	V‑ATPase accessory/assembly factor (Ac45); supports endosomal/lysosomal acidification	V‑ATPase activity is required for influenza virus entry, and V‑ATPase inhibition impairs ZIKV infection; ATP6AP1 is an essential V‑ATPase accessory factor, supporting inference that this module is a reusable pan-viral dependency at the entry/uncoating step	Models: A549/MDCK (IAV), Huh7 (ZIKV/DENV), Calu‑3 (SARS‑CoV‑2; endosomal entry contexts). Perturb: CRISPRi/siRNA (partial KD). Readouts: endosomal pH (pHrodo), early infection (2–6 h), RT‑qPCR/FFU; include bafilomycin A1 as pathway control	[54], [55]
ARF1	Small GTPase regulating ERGIC/Golgi trafficking and COPI recruitment	SARS‑CoV‑2: ARF1 interacts with viral M, promotes accumulation of M at ERGIC, pharmacologic ARF1 inhibition blocks virion production in cells and in vivo, providing a strong assembly/egress rationale and tractability	Models: Calu‑3 or A549‑ACE2 (SARS‑CoV‑2); optional A549 (IAV). Perturb: siRNA/CRISPRi and/or reported ARF1 inhibitors (as used in study). Readouts: M/ERGIC colocalization, extracellular vs intracellular viral RNA, PFU/FFU, EM/assembly markers if available	[56]
COPB1	COPI coatomer β subunit; retrograde Golgi↔ER and endomembrane trafficking	SARS‑CoV‑2: COPB1 supports SARS‑CoV‑2 replication organelle generation together with RAB5+ membranes. Cross-virus relevance: COPI-dependent trafficking contributes broadly to viral membrane logistics (entry/replication/assembly), making COPB1 a high-value trafficking node	Models: Calu‑3 (SARS‑CoV‑2), A549 (IAV), Huh7 (DENV/ZIKV). Perturb: CRISPRi/siRNA. Readouts: dsRNA/RO formation, viral protein localization (ERGIC/Golgi markers), RT‑qPCR, PFU/FFU	[53], [57]
XPO1	Exportin‑1/CRM1; nuclear export receptor for NES-bearing proteins/RNPs	IAV: CRM1 inhibition (leptomycin B) blocks nuclear export of influenza vRNPs. SARS‑CoV‑2: XPO1 inhibitor selinexor reduced SARS‑CoV‑2 infection in vitro and in vivo (ferret model), providing a tractable translational angle	Models: A549/MDCK (IAV), Calu‑3 (SARS‑CoV‑2). Perturb: selinexor or leptomycin B and/or siRNA/CRISPRi. Readouts: IAV NP/vRNP nuclear export (IF/fractionation), minigenome reporter; SARS‑CoV‑2 RT‑qPCR/FFU; viability	[58], [59]
IPO7	Importin‑7; importin‑β family nuclear import receptor	flaviviruses: Importin‑7 is required for nuclear translocation of flaviviral core protein and for infectious virus production, supporting a conserved nuclear-transport dependency relevant to DENV/ZIKV biology (and potentially host factor relocalization)	Models: Huh7 (DENV/ZIKV), relevant neural models for ZIKV. Perturb: CRISPRi/KO or siRNA. Readouts: core nuclear localization (IF), (FFU/PFU), RT‑qPCR; optionally nuclear import reporter control	[60]
EIF4A1	eIF4A helicase; RNA unwinding during translation initiation (eIF4F)	IAV: eIF4A inhibition (silvestrol/pateamine A) blocks IAV protein synthesis and replication. SARS‑CoV‑2: rocaglate CR‑31‑B inhibits SARS‑CoV‑2 replication at low nanomolar concentrations, supporting a cross-virus translation bottleneck	Models: A549 (IAV), Calu‑3 (SARS‑CoV‑2), Huh7 (flaviviruses). Perturb: eIF4A inhibitors (PatA/silvestrol/rocaglates) and/or CRISPRi. Readouts: nascent viral protein (puromycin labeling), RT‑qPCR, PFU/FFU; host translation controls	[61], [62]
PABPC1	Cytoplasmic poly(A)-binding protein; promotes translation initiation and mRNA stability	DENV: PABP binds at DENV 3′UTR and modulates translation efficiency, providing direct mechanistic support. SARS‑CoV‑2: direct SARS‑CoV‑2 RNA–protein interactome studies identify host RBPs involved in RNA utilization/translation, supporting cross-virus relevance of translation hubs	Models: Huh7 (DENV), Huh7 or Calu‑3 (SARS‑CoV‑2), A549 (IAV). Perturb: siRNA/CRISPRi. Readouts: luciferase replicon/translation reporters, polysome profiling (optional), RT‑qPCR/FFU; rescue with siRNA‑resistant PABPC1 if feasible	[63], [64]
DDX3X	DEAD-box RNA helicase; RNA metabolism/stress granules/innate-signaling interface	Selective DDX3X inhibitors suppress DENV replication; DDX3 targeting with RK‑33 reduces SARS‑CoV‑2 viral load in Calu‑3 cells	Models: Calu‑3 (SARS‑CoV‑2), Huh7 (DENV/ZIKV). Perturb: RK‑33 or related compounds ± siRNA/CRISPRi. Readouts: RT‑qPCR/FFU, IFNB1/ISGs (qPCR), stress granule markers (G3BP1) to interpret mechanisms; viability	[65], [66]
DDX46	Nuclear spliceosome helicase (U2 snRNP); suppresses innate immunity by nuclear retention of antiviral transcripts	DDX46 inhibits type I IFN induction after viral infection by entrapping antiviral transcripts in the nucleus, supporting it as a plausible proviral immune-evasion node across IFN-sensitive RNA viruses. (Direct linkage to the four specific viruses is primarily inferential from this mechanism.)	Models: Calu‑3 (SARS‑CoV‑2), A549 (IAV), Huh7 (DENV/ZIKV). Perturb: siRNA/CRISPRi. Readouts: IFNB1/ISGs (qPCR), RT‑qPCR/FFU; optional RNA localization assays for antiviral transcripts	[67]
HNRNPC	hnRNP C1/C2; RNA binding and pre‑mRNA processing; can modulate antiviral signaling	DENV: hnRNP C1/C2 supports DENV replication at the viral RNA synthesis stage. Virus-context evidence (coronaviruses/host response): hnRNP C can modulate coronavirus replication/host RNA programs, supporting cross-virus relevance at the RNA-processing interface	Models: Huh7 (DENV), Calu‑3 (SARS‑CoV‑2), A549 (IAV). Perturb: siRNA/CRISPRi. Readouts: viral RNA synthesis time course, intracellular vs released virus, IFN pathway markers; rescue for specificity	[68], [69]
SFPQ	DBHS-family nuclear RNP/splicing factor; regulates transcription/RNA processing	IAV: SFPQ/PSF is essential for influenza virus transcription and increases viral mRNA polyadenylation efficiency. SARS‑CoV‑2: SFPQ binds at the SARS‑CoV‑2 genome and promotes viral RNA amplification, supporting cross-virus RNA-processing dependence	Models: A549 (IAV), A549‑ACE2/Calu‑3 (SARS‑CoV‑2). Perturb: siRNA/CRISPRi. Readouts: IAV polymerase/minigenome + viral mRNA poly(A) assays (optional), SARS‑CoV‑2 RNA amplification (RT‑qPCR), FFU/PFU; viability	[47], [48]
SEC23B	COPII coat subunit; ER-to-Golgi vesicle budding/secretory trafficking	IAV screen/validation context: SEC23B identified/validated as a hit affecting IAV replication in a functional genomic screening. Cross-virus relevance: secretory trafficking is central for assembly/egress of enveloped RNA viruses including flaviviruses	Models: A549 (IAV), Huh7 (DENV/ZIKV). Perturb: CRISPRi/siRNA (partial KD). Readouts: intracellular vs extracellular virus, secretory reporter control (VSV‑G), RT‑qPCR/FFU, viral protein localization (ERGIC/Golgi markers)	[70], [71]
REEP5	ER membrane-shaping protein; supports ER architecture and remodeling	SARS‑CoV‑2: REEP5/TRAM1 binds SARS‑CoV‑2 NSP3 and promotes virus replication; REEP5 KO reduces ER rearrangements, providing direct mechanistic support related to replication-organelle biogenesis	Models: Calu‑3 or A549‑ACE2 (SARS‑CoV‑2); optional Huh7 (flaviviruses, ER replication). Perturb: CRISPR KO or CRISPRi. Readouts: dsRNA/replication organelle markers, ER morphology markers, RT‑qPCR/FFU; include viability controls	[72]
CTPS1	CTP synthetase; de novo CTP biosynthesis and immune-linked enzymatic functions	SARS‑CoV‑2 exploits CTPS1 to promote CTP synthesis and suppress IFN induction; CTPS inhibition impedes replication and supports the host-directed antiviral rationale	Models: Calu‑3 (SARS‑CoV‑2) ± A549 (IAV) for cross-virus check. Perturb: CTPS inhibitors ± CRISPRi. Readouts: viral RNA/protein, PFU/FFU, CTP levels (optional LC‑MS), IFN markers; cytidine rescue for specificity	[73]
SAE1	SUMO E1 activating enzyme subunit; initiates SUMOylation cascade	IAV: influenza NS1 is SUMOylated and influenza proteins interact with the host SUMOylation machinery; SAE1 is essential for SUMO activation, supporting a plausible proviral PTM dependency to test.	Models: A549 (IAV), Calu‑3 (SARS‑CoV‑2) exploratory. Perturb: CRISPRi/siRNA. Readouts: global SUMOylation (SUMO1/2/3 blots), viral replication (RT‑qPCR/FFU), and (if feasible) viral protein SUMOylation assays; viability	[74], [75]
HMGB1	Chromatin-associated DNA-binding protein; regulates transcription and inflammation; can bind viral proteins	IAV: HMGB1 associates with influenza NP in nuclei, promotes viral growth, and enhances polymerase activity; glycyrrhizin inhibits HMGB1-dependent influenza polymerase activity—supporting a mechanistically testable host factor in IAV	Models: A549 (IAV) ± Calu‑3 (SARS‑CoV‑2) exploratory. Perturb: siRNA/CRISPRi; optional glycyrrhizin as tool compound. Readouts: IAV minigenome/polymerase assay, NP localization, PFU/FFU; cytokine readouts to separate replication vs inflammation effects	[76]
RABGEF1	Rabex‑5; VPS9-domain GEF that activates RAB5 during early endocytosis	ZIKV/DENV: functional genomics identified endocytosis-related dependency factors including RABGEF-associated signals; given RABGEF1’s role as a Rab5 activator, it is a coherent upstream entry/trafficking candidate for flaviviruses and other endosome-using viruses	Models: Huh7 (DENV/ZIKV), A549 (IAV), Calu‑3 (SARS‑CoV‑2 endosomal entry contexts). Perturb: siRNA/CRISPRi. Readouts: Rab5 activation (optional GTP‑Rab5 pulldown), early antigen/entry, RT‑qPCR/FFU; compare phenotype to RAB5A perturbation	[77], [78]

### Gene set enrichment analysis reflects a broad spectrum of hijacked cellular processes

Next, we performed gene set enrichment analysis of the 118-consensus-genes. Most significantly enriched gene sets were related to gene regulation, followed by cellular component organization, metabolism, cell cycle and intracellular transport (Fig. 4A). For gene regulation and cell cycle, helicase activity and DNA/chromosome-organization gene sets were enriched, concordant with the known widespread reliance of viruses on host RNA helicases (e.g., DDX family [79]) and on host cell-cycle and DNA-damage control pathways during replication [80, 81]. For cellular component organization, we observed prominent enrichment of adhesion and adherens junction processes, including focal adhesion and cadherin-related modules that help maintaining endothelial and epithelial barrier integrity. This finding aligns with existing reports that flaviviral and coronaviral infections disrupt tight and adherens junctions, as well as cadherin-anchored complexes, to facilitate vascular leakage and viral dissemination [82, 83]. For metabolism, we mainly found enriched gene sets related to RNA metabolism, specifically processes involved in spliceosome/mRNA-binding and cytoplasmic RNP/stress-granule components, in line with evidence that RNA viruses can modulate host alternative splicing and actively remodel or antagonize stress granules during infection [84–86]. For transport, we observed enriched trafficking modules, including ER/Golgi and endosomal transport, nucleocytoplasmic transport, and the ubiquitin–proteasome–linked protein modifications required for these processes. This is consistent with extensive literature showing that viruses hijack the ubiquitin–proteasome system, exploit endomembrane routes for entry and egress, and make use of CRM1/Exportin-1–dependent nuclear export and nuclear pore complex pathways to support replication and immune evasion [87, 88].

To obtain a short list of consensus gene sets, we selected those that were significant for all four viruses. We observed 42 enriched pathways shared by all four viruses. Figure 4D shows the most significant gene sets, a complete list is provided in Table S10. The top, repeatedly enriched gene sets include nuclear transport, stress responses, protein quality control, gene-expression regulation, cellular-component organization, and cell-cycle/DNA activities, reflecting the central host processes utilized by these viruses.

### Accumulated view on the HDF and suggestions for host-directed inhibitors

**Fig. 5:**
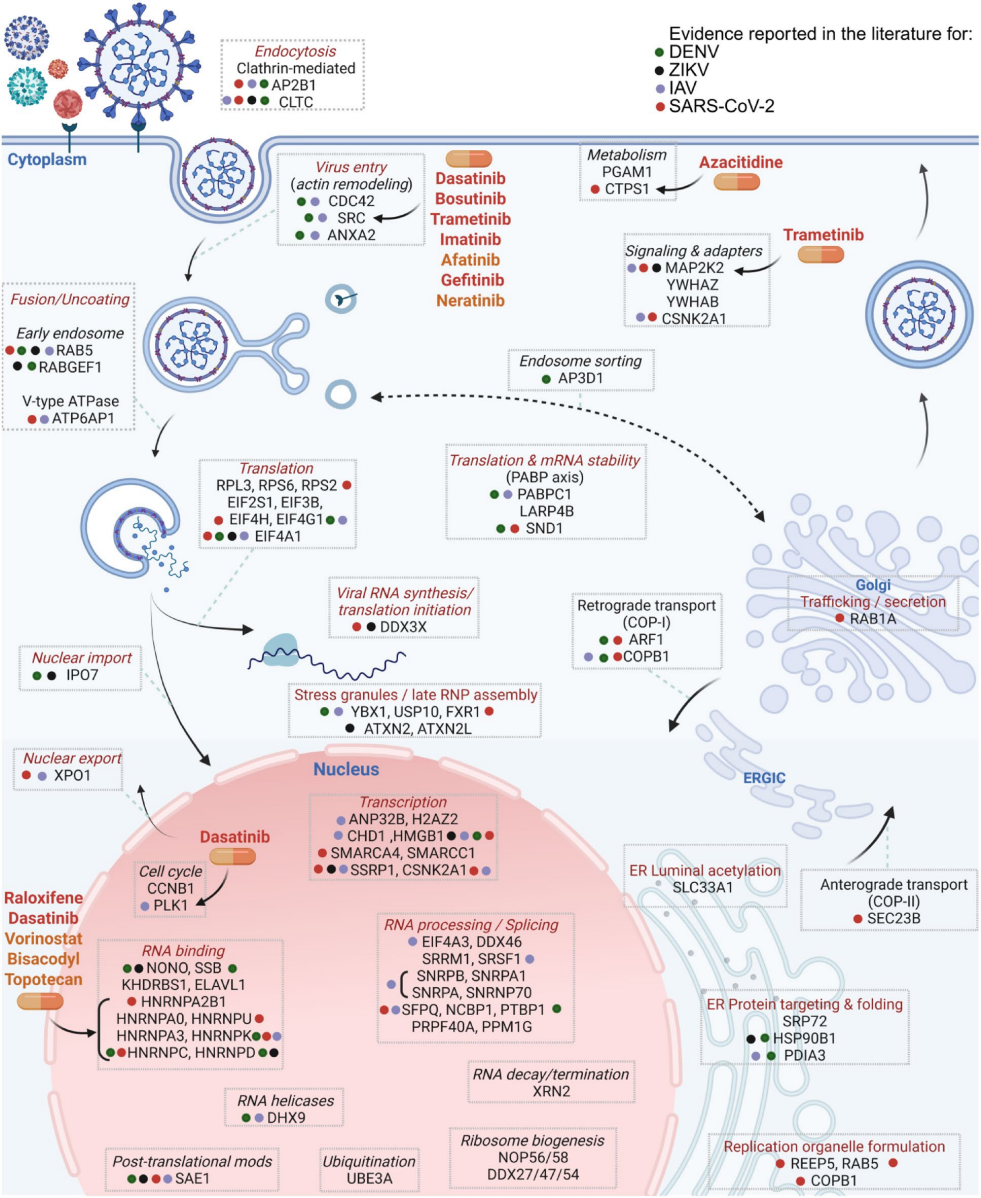
Simplified pan-viral life-cycle map of representative host-dependency genes. Selected HDF are positioned according to their predominant host-cell localization and/or functional role during infection. For IAV, nucleus-associated modules reflect *bona fide* nuclear steps of the viral life cycle, whereas for SARS-CoV-2, DENV, and ZIKV they denote nucleus-associated or nucleo-cytoplasmic host factors rather than proteins for nuclear viral replication. Pale blue guide lines connect each infection stage to the corresponding host-factor module, and red module titles indicate the viral process predicted to be impaired upon perturbation of the genes listed in that box. Colored dots next to individual genes denote prior experimental literature support identifying that gene as an HDF for the indicated virus (green, DENV; black, ZIKV; purple, IAV; red, SARS-CoV-2). Orange drug labels indicate approved drugs predicted to target proteins in the adjacent module, whereas red drug labels highlight compounds with reported antiviral activity against related RNA viruses. For clarity, only representative genes of a family are shown

Figure 5 provides an overview of the 118-consensus-genes mapped to a schematic representing the viral life cycle across ZIKV, DENV, and SARS-CoV-2 (without nucleus), and IAV (including the nucleus). Colored dots adjacent to each gene denote prior experimental validation in the literature for each virus, with green representing DENV, black ZIKV, purple IAV, and red SARS-CoV-2. Comprehensive gene-level evidence for all viruses is compiled in Table S11. The corresponding findings from the literature are summarized in Discussion.

To gain more granular insight into the cellular processes represented in this pan-viral backbone, we regarded the larger list of 427-core-genes and applied a gene prioritization process selecting genes which are, well-expressed in infected cells, centrally positioned in the host interaction network, and at least partially documented in the literature. This led to a list of 210 prioritized genes, whose network organization and gene-family composition we examined. Our analysis revealed again dense modules of translation factors, DEAD-box RNA helicases, nuclear transport proteins, endocytic regulators, and components of the ubiquitin–proteasome system. These findings indicate that conserved pan-viral host dependencies cluster around protein synthesis, RNA metabolism, intracellular trafficking, and proteostasis (see Supplementary Text S2) (Fig. S4–S5).

To identify and prioritize the most promising drug candidates, we employed the Drug–Target Interaction and Affinity Mechanism (DTIAM) framework, a recently developed model that predicts drug–target interaction (DTI), binding affinity (DTA), and mechanism of action (MoA; activation vs. inhibition) and shows strong performance even in cold-start settings [89]. We retained only those predicted drug – target pairs that met stringent criteria: a predicted DTI score greater than 0.70, a predicted potency of at least 5 on the pIC50 scale, and a MoA classified as inhibition of the target. The complete list of candidates is provided in Table S12. Several of the top-scoring drugs already have experimental support, lending external validation to our prioritization. The MEK inhibitor trametinib, which targets the MAPK kinases MEK1 and MEK2 (MAP2K1 and MAP2K2), consistently suppressed IAV, ZIKV, DENV and SARS-CoV-2 replication in vitro and improved lung pathology in mouse models of severe influenza and SARS-CoV-2 infection [90, 91]. Src/Abl TKIs (dasatinib [92, 93], bosutinib [91, 94], imatinib [95, 96]) inhibit coronavirus and influenza virus entry and/or egress in several cell-based systems in vitro. However, so far, their antiviral activity in vivo appears limited and model-dependent. For example, in a golden Syrian hamster model of SARS-CoV-2 lung infection, orally administered imatinib did not reduce lung viral RNA loads [97]. EGFR/ErbB-pathway inhibitors (afatinib, gefitinib, neratinib) also emerged from our screen, mainly via predicted interactions with MAP2K1/2, and have been reported to curtail DENV and ZIKV replication [98] and to reduce SARS-CoV-2 entry in selected cell lines, consistent with a role of EGFR–MEK signaling in viral infection [99, 100]. Epigenetic drugs were likewise prioritized. The DNA-methyltransferase inhibitor azacitidine, which depletes DNMT1, reduced SARS-CoV-2 replication and improved survival in infected mice but carries a risk of myelosuppression [101]. Raloxifene, a selective oestrogen-receptor modulator that primarily targets ESR1—identified by our framework as an HDF for SARS-CoV-2 and DENV—exhibited broad-spectrum antiviral activity in vitro against flaviviruses and SARS-CoV-2 and accelerated viral clearance in a phase II trial in patients with mild COVID-19 [102–104] whereas vorinostat targeting class I HDACs including HDAC1 from our HDF set produced mixed, occasionally pro-viral effects, depending on the context [105]. Finally, bisacodyl, predicted to inhibit several lower-ranked HDF, inhibited Chikungunya virus (an alphavirus) in vitro [106], but we did not find data for IAV, SARS-CoV-2, DENV or ZIKV and therefore treated it as a lower-priority candidate in the current study.

### Comparison of the identified HDF signatures with the prioritized HDF of EBOV

To assess out-of-family generalization, we asked whether the identified HDF compared to the HDF screening data from EBOV. For EBOV, we used the list of prioritized genes generated by MAIC. We overlapped this list with the consensus and core genes. Table S13 shows the results. Indeed, the 427-core-genes were strongly over-represented among MAIC-prioritized EBOV candidates. We found 44 in the top 200 EBOV genes, which was 9.3 times more compared to a random selection (*p* = 5E-30). We observed similar enrichments using other cutoffs for the top genes of EBOV (23/100, 84/500, 128/1000 with *p*-values 1.1e-16, 1.9e-47, and 1.6e-59, respectively). The stricter gene set of 118-consensus-genes was also highly enriched (41/1000; ~6.3 times more represented compared to a random selection, *p* = 2E-22). In summary, we found considerably good overlaps between the identified core and consensus genes when compared to top ranking genes derived from experimental screening data of EBOV.

## Discussion

The key aim of this study was to obtain a pan-viral overview of HDF from molecular screening data. We integrated a large range of experimentally complementary data sources, using linear regression followed by AI-based models to overcome the limitations of individual approaches. Previous studies have noted limited concordance between genome-wide knockout and knockdown screens, even when studying the same virus [3, 13]. MAIC iteratively up-weights genes and gene lists that show consistent evidence of being HDF across diverse multi-omics datasets, including CRISPR/Cas9 and RNAi screens, viral protein–protein and RNA–protein interactomes, and single-cell RNA-seq of infected cells [3]. MAIC was employed to the screening data for benchmarking. Machine learning was employed on this benchmarking dataset using gene descriptors based on a molecular network of the host cell and a wide range of intrinsic and extrinsic gene characteristics. We observed an excellent performance across four viruses (AUROC > 0.90 for SARS-CoV-2, IAV, ZIKV, and DENV) demonstrating the power of this integrative approach. Particularly for SARS-CoV-2 and IAV the attention network models led to superior prediction performance likely due to the rich data source which is available for these two virus species. For EBOV the performance was considerably weaker, which we attribute to the limited availability and quality of benchmarking data. Consistently, using single data sources or unweighted labels markedly reduced performance for the other four viruses as well. Exemplarily, using only the CRISPR knockout data led to a balanced accuracy between 0.61 and 0.64 across the studied other four viruses. In the following, we discuss the results from the other four viruses (IAV, SARS-CoV-2, DENV, ZIKV) based on the full benchmarking data. In particular, the heterogeneous graph attention network (HAN) approach was effective in capturing the complex relationships between host and viral proteins. By walking over meta-paths in the host-virus interaction network, the model could leverage higher-order connectivity patterns.

We identified a consensus set of 118 pan-viral HDF shared among all four viruses and 427 genes common to at least three of them. To quantify how integration sharpens cross-virus agreement, we compared pairwise overlaps between virus-specific top-ranked genes at three levels: raw experimental lists, MAIC-prioritized rankings and final machine-learning scores. The mean cross-virus overlap, increased from 13.45% for the original experimental lists to 25.63% after MAIC integration up to 40% for the machine-learning predictions, indicating that integration and model-based prioritisation substantially increased the consistency of inferred HDF across viruses.

An important limitation of the present work is that it remains a prediction-based study and therefore represents a starting point rather than an endpoint for translational validation. Although the AI models achieved strong discrimination and improved cross-virus consistency, the resulting rankings should be interpreted as prioritized hypotheses requiring direct experimental testing. To increase the practical value of the study, we provide a manually curated shortlist from the 118 genes predicted across all four viruses (Table 1), selected as the, in our eyes, most useful candidates for initial laboratory follow-up based on mechanistic interpretability, literature support, life-cycle coverage, and tractability for perturbation assays. The full 118-gene consensus set is provided in Table S11 as a broader resource for subsequent validation and expansion. In the following, without claiming to be exhaustive, we discuss key consensus genes and their literature-derived context as a pan-viral model of HDF and their functional roles. In the following text, genes belonging to the 118-gene consensus set are highlighted in bold. The identified HDF align closely with critical stages of the viral life cycle. Entry and early organelle remodeling are gated by clathrin-mediated endocytosis (**AP2B1, CLTC**), **RAB5/RABGEF1** early endosomes and **ATP6AP1** V-ATPase, followed by **AP3D1** endosome sorting [107]. For viral entry, clathrin-mediated endocytosis serves as a prevalent mechanism. IAV recruits the **AP2B1–CLTC** clathrin adaptor complex to facilitate uptake [49], while flaviviruses similarly utilize clathrin/**RAB5**-driven endosomes [108]. Furthermore, **AP2B1/CLTC**-driven endocytosis, with **RAB5** connects to junctional barrier disruption that flaviviruses and coronaviruses induce to enter tissues [109]. Subsequently, vesicular trafficking between the ER and the Golgi apparatus is another essential component of the viral life cycle. **ARF1/COPB1** (COPI) and **SEC23B** (COPII) facilitate the bidirectional transport of viral proteins, while **SRP72** directs nascent membrane proteins into the ER. Besides this, **RAB5**, along with its guanine nucleotide exchange factor (GEF) **RABGEF1**, and the V-ATPase subunit **ATP6AP1**, are essential for acidified endosomal fusion of DENV and ZIKV [110]. Notably, nuclear-cytoplasmic transport factors were identified as HDF: **XPO1/CRM1** is responsible for exporting influenza vRNPs and flavivirus NS5 [111], and **IPO7** transports flavivirus core proteins into the nucleus, which is essential for the release of infectious particles [60]. These mechanistic roles are consistent with our pathway analysis, in which we identified predicted HDF enrichment in viral entry exploiting endocytic and adherens junction pathways, and viral egress involving the ER/Golgi and endosomal systems. Following viral entry, the expression of viral genes commandeers the host’s RNA machinery. We identified critical translation factors **EIF4A1** and **PABPC1**, known to be responsible for unwinding 5′-UTRs and bridging mRNA ends. We listed nine heterogeneous nuclear ribonucleoproteins (hnRNPs), such as **hnRNPK/C**. HnRNPs emerged as regulators of splicing for IAV M/NS transcripts and are likely to bind flaviviral UTR to enhance translation [112]. These findings reflect established viral strategies, wherein RNA viruses globally manipulate the spliceosome and stress-granule proteins needed for viral mRNA processing [112, 113]. Additionally, we identified multiple DEAD-box RNA helicases, including **DDX3X**, which facilitates viral RNA synthesis and innate signaling, and **DDX46**, which suppresses antiviral transcripts by sequestering them in the nucleus [67]. By attenuating type-I interferon production, **DDX46** exemplifies how viruses exploit host helicases to evade immune responses. Collectively, the predicted HDF underscore that influenza, SARS-CoV-2, and flaviviruses exploit host translation initiation, RNA chaperones, and splicing factors to drive the expression and maturation of their genes, consistent with the strong enrichment of “RNA metabolism” and “helicase” gene sets in our consensus set.

Further identified HDF reflect hijacking of metabolism, proteostasis, and immune regulation. Metabolic enzymes CTP synthase 1 (**CTPS1**) and phosphoglycerate mutase 1 (**PGAM1**) suggest that the viruses employ nucleotide and glycolysis to fuel replication. Indeed, SARS-CoV-2 has been shown to exploit **CTPS1** to increase CTP synthesis and to simultaneously suppress interferon [73]. Phosphoglycerate mutases are essential in glycolysis converting 3-phosphoglycerate to 2-phosphoglycerate. The ubiquitin–proteasome system is also part of our pan viral model. E3 ligases **WWP1** and **UBE2N** likely regulate degradation of host restriction factors or viral protein processing, and SUMOylation (via **SAE1**) modifies viral proteins (IAV NP, flavivirus NS5, SARS-CoV-2 N) to enhance their function [114–116]. We identified factors typically associated with gene regulation via host chromatin binding, such as **SSRP1** (of the FACT complex) and **HMGB1**. FACT regulates chromatin accessibility and can suppress antiviral gene programs [117]. The **HMGB1** gene encodes the high-mobility group box 1 protein, which, in the nucleus, binds DNA and supports chromatin organization, facilitating gene transcription, DNA repair, and genome stability. When released from the nucleus, it acts as a potent proinflammatory alarmin that can amplify viral replication and inflammation [118]. These genes connect cell-cycle regulation with nucleotide sequence remodeling as the viruses rely on host DNA-remodeling and the helicase machinery. To note, certain nuclear splicing proteins we found in our lists (like **SFPQ** and hnRNPs) need to be relocalized into the cytoplasm during infection to assist viral RNA processes, in line with the known interesting observation for several viruses that nuclear host factors become pro-viral in the cytosol after infection. As many viruses, SARS-CoV-2 forms replication organelles by rearranging host cellular membranes. SARS-CoV-2 forms double-membrane vesicles via NSP3/4 [119], and **REEP5,** located in endoplasmic reticulum tubular network, was observed to be involved in this process [72]. Interestingly, it was observed that SARS-CoV-2 needs membranes containing **RAB5** to build replication organelles, supported by **COPB1** [53].

To assess out-of-family generalization, we asked whether the pan-viral signature transfers to filoviruses. Although EBOV data are sparser and were not used to define our consensus and core gene sets, both pan-viral sets derived from IAV, SARS-CoV-2, DENV, and ZIKV, i.e., the 427-core-genes and the 118-consensus-genes, were highly over-represented among MAIC-prioritized EBOV candidates. Indeed, when interrogating the literature, we recognized that several of the overlapping genes have prior experimental support in filovirus biology including ILF2, RPS6, RPL3, HSP90AB1, GSPT1, AHCY, and RAB7A [120–126], underscoring our approach to develop a pan-viral consensus list of HDF of RNA viruses.

As a preliminary exploratory test of framework transferability to oncogenic viruses, we examined Epstein–Barr virus (EBV), which provided the richest usable high-throughput data among the oncogenic viruses for which we evaluated data availability. Despite this comparatively favorable starting point, the final EBV benchmark remained sparse and biologically heterogeneous, with limited overlap across retained evidence layers and no transcriptome or RNA-centric component. Predictive performance was therefore modest (AUC–ROC ~0.63), indicating that extension to oncogenic viruses will likely require phase-specific benchmarking and better-balanced evidence layers. Further details are provided in Supplementary Text S3.

Collectively, our predicted HDF span the major host pathways, such as clathrin/endosome-mediated entry and adhesion/junction remodeling, ER–Golgi trafficking, translation initiation, RNA metabolism, chromatin/helicase functions, while simultaneously dampening host immune programs. To translate these insights into therapies, we used DTIAM, a foundation model based compound – target prediction tool, applied to the identified HDF. Noteworthy candidates included trametinib, Src/Abl TKIs (dasatinib, bosutinib, imatinib), EGFR/ErbB inhibitors (afatinib, gefitinib, neratinib), azacitidine, raloxifene, vorinostat, topotecan, and bisacodyl. Encouragingly, many of our predictions matched experimental observations. Trametinib emerged among our top host-directed candidates. It was shown that trametinib blocked multiple viruses. Trametinib is a clinically approved MEK1/2 inhibitor. Numerous RNA viruses co-opt the host Raf–MEK–ERK signaling cascade for essential steps of their life cycle. Pharmacologic MEK blockade can suppress viral replication while attenuating excessive pro-inflammatory cytokine production [127]. In vitro, trametinib effectively inhibited IAV replication and reduced virus-induced cytokine expression [128]. It also exhibited pan-antiviral efficacy against flaviviruses activity, achieving an about 1,000-fold reduction in ZIKV and Yellow Fever Virus titers and nearly 100-fold reduction in DENV (DENV-2/3) titers [91]. For coronaviruses, MEK inhibition has also shown efficacy. Trametinib strongly inhibited MERS-CoV infection in human cells, outperformed selumetinib (another MEK inhibitor) in head-to-head assays, and showed efficacy against SARS-CoV-2 in vitro, reducing viral infection and associated cytokine responses in cell cultures [129]. Beyond MEK inhibition, several of the other predicted compounds also have experimental support. Src/Abl tyrosine kinase inhibitors (dasatinib, bosutinib, imatinib) and EGFR/ErbB inhibitors (afatinib, gefitinib, neratinib) have been reported to impair coronavirus, influenza or flavivirus infection in cell-based and, in some cases, in vivo models, and agents such as azacitidine, raloxifene, vorinostat and topotecan likewise reduce viral replication or infection-induced pathology in preclinical studies. This convergence between literature data and our model-based rankings further supports the biological plausibility of the proposed drug–HDF pairs. It will be important and intriguing to test the newly predicted drugs experimentally against the studied viruses.

In conclusion, our integrative machine learning approach showed excellent performance and increased commonalities in predicted HDF among the studied viruses demonstrating the power of combining diverse data modalities powered by AI methods to overcome the limitations of individual experimental techniques. The study provides a comprehensive map of pan-viral HDF across four major RNA viruses, revealing conserved host mechanisms. The HDF signature we derived exhibits considerable overlap with potential HDF of EBOV, suggesting that these conserved mechanisms extend to other RNA viruses. The identified HDF and potential drug candidates offer promising avenues for future experimental validation and therapeutic development. As new viral threats continue to emerge, such pan-viral approaches will be crucial for rapidly identifying potential intervention strategies.

## Electronic supplementary material

Below is the link to the electronic supplementary material.

Supplementary Material 1

Supplementary Material 2

Supplementary Material 3

## Data Availability

All data generated or analyzed during this study are included in this published article and its supplementary information files. All codes and scripts used in this study are available at https://github.com/Naseri1374/panviral-hdf. An interactive Shiny web application for exploring the pan-viral HDF, their machine-learning scores, MAIC evidence and functional enrichment results is available at https://1uzj53-mohadese-naseri.shinyapps.io/panviral_hdf_shiny_app/.

## References

[1] Li S, Li H, Lian R, Xie J, Feng R. New perspective of small-molecule antiviral drugs development for RNA viruses. Virology [Internet]. 2024;594:110042. Available from: https://www.sciencedirect.com/science/article/pii/S0042682224000631. 2025 Oct 7. 38492519 10.1016/j.virol.2024.110042

[2] Hou J, Wei Y, Zou J, Jaffery R, Sun L, Liang SZheng N, et al. Integrated multi-omics analyses identify anti-viral host factors and pathways controlling SARS-CoV-2 infection. Nat Commun [Internet]. 2024;15(1):1–14. Available from: https://www.nature.com/articles/s41467-023-44175-1. 2025 Oct 12. 38168026 10.1038/s41467-023-44175-1 PMC10761986

[3] Li B, Clohisey SM, Chia BS, Wang B, Cui A, Eisenhaure T, et al. Genome-wide CRISPR screen identifies host dependency factors for influenza a virus infection. Nat Commun. 2020;11(1):11. 10.1038/s41467-019-13965-x. 31919360 10.1038/s41467-019-13965-x PMC6952391

[4] Dengue [Internet]. [https://www.who.int/news-room/fact-sheets/detail/dengue-and-severe-dengue. 2025 Oct 12].

[5] Zika virus [Internet]. [https://www.who.int/news-room/fact-sheets/detail/zika-virus. 2025 Oct 12].

[6] Ebola disease [Internet]. [https://www.who.int/news-room/fact-sheets/detail/ebola-disease. 2025 Oct 12].

[7] Badia R, Garcia-Vidal E, Ballana E. Viral-host dependency factors as therapeutic targets to overcome antiviral drug-resistance: a focus on innate immune modulation. Front Virol [Internet]. 2022;2:935933. Available from: https://www.frontiersin.org. 2025 Oct 12.

[8] Adderley J, Grau GE. Host-directed therapies for malaria: possible applications and lessons from other indications. Curr Opin Microbiol [Internet]. 2023;71:102228. Available from: https://www.sciencedirect.com/science/article/pii/S1369527422001126. 2025 Oct 7. 36395572 10.1016/j.mib.2022.102228

[9] Chitalia VC, Munawar AH. A painful lesson from the COVID-19 pandemic: the need for broad-spectrum, host-directed antivirals. J Transl Med. 2020;18], 18, 181 [390– [Internet]. Available from: 1). 10.1186/s12967-020-02476-9. 2026 Feb 25. 10.1186/s12967-020-02476-9 PMC7558548 33059719

[10] Kaufmann SHE, Dorhoi A, Hotchkiss RS, Bartenschlager R. Host-directed therapies for bacterial and viral infections. Nat Rev Drug Discov [Internet]. 2018;17(1):35–56. Available from: 10.1038/nrd.2017.162. 28935918 10.1038/nrd.2017.162 PMC7097079

[11] Kumar N, Sharma S, Kumar R, Tripathi BN, Barua S, Ly H, et al. Host-directed antiviral therapy. Clin Microbiol Rev [Internet]. 2020;33(3):e00168–19. Available from: https://pmc.ncbi.nlm.nih.gov/articles/PMC7227448/. 2026 Feb 25. 10.1128/CMR.00168-19 PMC7227448 32404434

[12] Sakai M, Masuda Y, Tarumoto Y, Aihara N, Tsunoda Y, Iwata M, et al. Genome-scale CRISPR-Cas9 screen identifies host factors as potential therapeutic targets for SARS-CoV-2 infection. iScience [Internet]. 2024;27(8):110475. Available from: https://www.sciencedirect.com/science/article/pii/S2589004224017000. 2025 Oct 7. 39100693 10.1016/j.isci.2024.110475 PMC11295705

[13] Rebendenne A, Roy P, Bonaventure B, Chaves Valadão AL, Desmarets L, Arnaud-Arnould M, et al. Bidirectional genome-wide CRISPR screens reveal host factors regulating SARS-CoV-2, MERS-CoV and seasonal HCoVs. Nat Genet. 2022;54(8):1090–102. 10.1038/s41588-022-01110-2. 35879413 10.1038/s41588-022-01110-2 PMC11627114

[14] Parkinson N, Rodgers N, Head Fourman M, Wang B, Zechner M, Swets MC, et al. Dynamic data-driven meta-analysis for prioritisation of host genes implicated in COVID-19. Sci Rep [Internet]. 2020;10(1):1–12. Available from: https://www.nature.com/articles/s41598-020-79033-3. 2025 Sep 11. 33339864 10.1038/s41598-020-79033-3 PMC7749145

[15] Maes A, Botzki A, Mathys J, Impens F, Saelens X. Systematic review and meta-analysis of genome-wide pooled CRISPR screens to identify host factors involved in influenza a virus infection. J Virol. 2024;98(5):98. 10.1128/jvi.01857-23. 10.1128/jvi.01857-23 PMC11257101 38567969

[16] Kelch MA, Vera-Guapi A, Beder T, Oswald M, Hiemisch A, Beil N, et al. Machine learning on large scale perturbation screens for SARS-CoV-2 host factors identifies β-catenin/CBP inhibitor PRI-724 as a potent antiviral. Front MicrobiolFront. Microbiol. 2023;14:14. 10.3389/fmicb.2023.1193320. 10.3389/fmicb.2023.1193320 PMC10277617 37342561

[17] Breiman L. Random forests. Mach Learn. 2001;45(1):5–32. Available from: 10.1023/A:1010933404324. 2025 Oct 13.

[18] Chen T, Guestrin C. Xgboost: a scalable tree boosting system. Proc ACM SIGKDD Int Conf Knowl Discov Data Min [Internet]. 2016 [cited 2025 Oct 13];13-17-2016:785–94. https://arxiv.org/pdf/1603.02754.

[19] Li W, Xu H, Xiao T, Cong L, Love MI, Zhang F, et al. MAGeCK enables robust identification of essential genes from genome-scale CRISPR/Cas9 knockout screens. Genome Biol. 2014;15(12):554. 10.1186/s13059-014-0554-4. 25476604 10.1186/s13059-014-0554-4 PMC4290824

[20] Luo B, Cheung HW, Subramanian A, Sharifnia T, Okamoto M, Yang X, et al. Highly parallel identification of essential genes in cancer cells. Proc Natl Acad Sci USA. 2008;105(51):20380–85. 10.1073/pnas.0810485105. 19091943 10.1073/pnas.0810485105 PMC2629277

[21] König R, Chiang CY, Tu BP, Yan SF, DeJesus PD, Romero A, et al. A probability-based approach for the analysis of large-scale RNAi screens. Nat Methods. 2007;4(10):847–49. 10.1038/nmeth1089. 17828270 10.1038/nmeth1089

[22] Hao Y, Stuart T, Kowalski MH, Choudhary S, Hoffman P, Hartman A, et al. Dictionary learning for integrative, multimodal and scalable single-cell analysis. Nat Biotechnol [Internet]. 2024;42(2):293–304. Available from: https://pubmed.ncbi.nlm.nih.gov/37231261/. 2025 Oct 13. 37231261 10.1038/s41587-023-01767-y PMC10928517

[23] Pearson FRSKP. LIII. On lines and planes of closest fit to systems of points in space. London, Edinburgh, Dublin Lond, Edinburgh Dublin Phil Mag J Sci [Internet]. 1901;2(11):559–72. Available from: https://scholar.google.com/scholar_url?url=https://www.tandfonline.com/doi/pdf/10.1080/14786440109462720%3Fcasa_token%3DcsKufH62bkkAAAAA:Ndb5zioz-awGF7ocKeQELVUt6uee2hh-dwEfvEPeE-EebBwjEMy9gpCgdVje9prgn14xsjAuUKuX%26hl=en%26sa=T%26oi=ucasa%26ct=ucasa%26ei=QPrsaJuKD6SHieoP6KSBoQ4%26scisig=AAZF9b88ySA5pIhWXIoZdXcvB17z. 2025 Oct 13.

[24] McInnes L, Healy J, Saul N, Großberger L. UMAP: uniform manifold Approximation and projection for dimension reduction. 2018, Available from: J Educ Chang Open Source Softw. 2018;3(29):861. 10.21105/joss.00861.

[25] Morabito S, Reese F, Rahimzadeh N, Miyoshi E, Swarup V. hdWGCNA identifies co-expression networks in high-dimensional transcriptomics data. Cell Rep Methods [Internet]. 2023;3(6):100498. Available from: 10.1016/j.crmeth.2023.100498. 37426759 10.1016/j.crmeth.2023.100498 PMC10326379

[26] Verschueren E, Von Dollen J, Cimermancic P, Gulbahce N, Sali A, Krogan NJ. Scoring large-scale affinity purification mass spectrometry datasets with MiST. CP Bioinf [Internet]. 2015;49(1):8.19.1. Available from: https://pmc.ncbi.nlm.nih.gov/articles/PMC4378866/. 2025 Oct 13. 10.1002/0471250953.bi0819s49 PMC4378866 25754993

[27] Choi H, Liu G, Mellacheruvu D, Tyers M, Gingras AC, Nesvizhskii AI. Analyzing protein-protein interactions from affinity purification-mass spectrometry data with SAINT. Curr protoc bioinformatics [Internet]. 2012. https://pmc.ncbi.nlm.nih.gov/articles/PMC3446209/. 2025 Oct 13. 10.1002/0471250953.bi0815s39 PMC3446209 22948729

[28] Beder T, Aromolaran O, Dönitz J, Tapanelli S, Adedeji EO, Adebiyi E, et al. Identifying essential genes across eukaryotes by machine learning. NAR Genomics Bioinf. 2021;3(4):1–13. 10.1093/nargab/lqab110. 10.1093/nargab/lqab110 PMC8634067 34859210

[29] Aromolaran O, Beder T, Oswald M, Oyelade J, Adebiyi E, Koenig R. Essential gene prediction in drosophila melanogaster using machine learning approaches based on sequence and functional features. Comput Struct Biotechnol J [Internet]. 2020;18:612–21. Available from: https://www.sciencedirect.com/science/article/pii/S2001037019305628?via%3Dihub. 2025 Sep 24. 32257045 10.1016/j.csbj.2020.02.022 PMC7096750

[30] Aromolaran O, Beder T, Adedeji E, Ajamma Y, Oyelade J, Adebiyi E, et al. Predicting host dependency factors of pathogens in drosophila melanogaster using machine learning. Comput Struct Biotechnol J [Internet]. 2021;19:4581–92. Available from: 10.1016/j.csbj.2021.08.010. 34471501 10.1016/j.csbj.2021.08.010 PMC8385402

[31] Charif D, Thioulouse J, Lobry JR, Perrière G. Online synonymous codon usage analyses with the ade4 and seqinR packages. Bioinformatics [Internet]. 2005;21(4):545–47. Available from: 10.1093/bioinformatics/bti037. 2025 Oct 13. 15374859 10.1093/bioinformatics/bti037

[32] Xiao N, Cao DS, Zhu MF, Xu Q-S. Protr/ProtrWeb: R package and web server for generating various numerical representation schemes of protein sequences. Bioinformatics [Internet]. 2015;31(11):1857–59. Available from: 10.1093/bioinformatics/btv042. 2025 Oct 13. 25619996 10.1093/bioinformatics/btv042

[33] Szklarczyk D, Kirsch R, Koutrouli M, Nastou K, Mehryary F, Hachilif R, et al. The STRING database in 2023: protein–protein association networks and functional enrichment analyses for any sequenced genome of interest. Nucleic Acids Res [Internet]. 2023;51(D1):D638–46. Available from: https://pubmed.ncbi.nlm.nih.gov/36370105/. 2025 Dec 2. 10.1093/nar/gkac1000 PMC9825434 36370105

[34] Hörhold F, Eisel D, Oswald M, Kolte A, Röll D, Osen W, et al. Reprogramming of macrophages employing gene regulatory and metabolic network models. PLoS Comput Biol [Internet]. 2020;16(2):e1007657. Available from: 10.1371/journal.pcbi.1007657. 2025 Nov 3. 10.1371/journal.pcbi.1007657 PMC7059956 32097424

[35] Smedley D, Haider S, Ballester B, Holland R, London D, Thorisson G, et al. BioMart – biological queries made easy. BMC Genomics [Internet]. 2009;10(1):1–12. Available from: 10.1186/1471-2164-10-22. 2025 Oct 13. 19144180 10.1186/1471-2164-10-22 PMC2649164

[36] Pruitt KD. NCBI Reference sequence (RefSeq): a curated non-redundant sequence database of genomes, transcripts and proteins. Nucleic Acids Res [Internet]. 2004;33(Database issue):D501–4. Available from: 10.1093/nar/gki025. 2025 Oct 13. 10.1093/nar/gki025 PMC539979 15608248

[37] Altschul SF, et al. Gapped BLAST and PSI-BLAST: a new generation of protein database search programs. Nucleic Acids Res [Internet]. 1997;25(17):3389–402. Available from: 10.1093/nar/25.17.3389. 2025 Oct 13. 9254694 10.1093/nar/25.17.3389 PMC146917

[38] Ravindran V, Wagoner J, Athanasiadis P, Den Hartigh AB, Sidorova JM, Ianevski A, et al. Discovery of host-directed modulators of virus infection by probing the SARS-CoV-2–host protein–protein interaction network. Briefings Bioinf [Internet]. 2022;23(6):1–14. Available from: 10.1093/bib/bbac456. 2025 Oct 13. 10.1093/bib/bbac456 PMC9677461 36305426

[39] Gordon DE, Jang GM, Bouhaddou M, Xu J, Obernier K, White KM, et al. A SARS-CoV-2 protein interaction map reveals targets for drug repurposing. Nature. 2020;583(7816):459–68. 10.1038/s41586-020-2286-9. 32353859 10.1038/s41586-020-2286-9 PMC7431030

[40] Grover A, Leskovec J. Node2vec: scalable feature learning for networks. Proc ACM SIGKDD Int Conf Knowl Discov Data Min. 2016;13-17:855–64. 10.1145/2939672.2939754 PMC5108654 27853626

[41] Almagro Armenteros JJ, Sønderby, Sønderby, Nielsen H, Winther O, Sønderby CK, et al. DeepLoc: prediction of protein subcellular localization using deep learning. Bioinformatics [Internet]. 2017;33(21):3387–95. Available from: 10.1093/bioinformatics/btx431. 2025 Oct 13. 29036616 10.1093/bioinformatics/btx431

[42] Raudvere U, Kolberg L, Kuzmin I, Arak T, Adler P, Peterson H, et al. G: profiler: a web server for functional enrichment analysis and conversions of gene lists (2019 update). Nucleic Acids Res [Internet]. 2019;47(W1):W191–8. Available from: https://pubmed.ncbi.nlm.nih.gov/31066453/. 2025 Nov 25. 10.1093/nar/gkz369 PMC6602461 31066453

[43] Keogh FD, Marx J, Hiemisch A, Koenig R. Slimformer: an NLP-based web server for semantic categorization of gene sets. Comput Struct Biotechnol J [Internet]. 2025;27:5252–62. Available from: https://linkinghub.elsevier.com/retrieve/pii/S2001037025005021. 2025 Nov 25. 41340889 10.1016/j.csbj.2025.11.035 PMC12671349

[44] Zhang Y, Jia Z, Yuan G, Chen K, Cen J, Wang J, et al. HnRNPC triggers the degradation of MITA to suppress the interferon-mediated antiviral response. Vet Res [Internet]. 2025. https://pmc.ncbi.nlm.nih.gov/articles/PMC11854013/. 2025 Nov 1. 10.1186/s13567-025-01463-6 PMC11854013 39994817

[45] Kesavardhana S, Samir P, Zheng M, Subbarao Malireddi RK, Karki R, Sharma BR, et al. DDX3X coordinates host defense against influenza virus by activating the NLRP3 inflammasome and type I interferon response. J Biol Chem [Internet]. 2021;296:100579. https://pubmed.ncbi.nlm.nih.gov/33766561/. 2025 Nov 1]. 33766561 10.1016/j.jbc.2021.100579 PMC8081917

[46] Yang SNY, Atkinson SC, Audsley MD, Heaton SM, Jans DA, Borg NA. RK-33 Is a Broad-Spectrum Antiviral Agent That Targets DEAD-Box RNA Helicase DDX3X. Cells. 2020 [cited 2025 Nov 1];9(1):170. Available from: https://www.mdpi.com/2073-4409/9/1/170/htm. 10.3390/cells9010170 PMC7016805 31936642

[47] Landeras-Bueno S, Jorba N, Pérez-Cidoncha M, Ortín J. The splicing factor proline-glutamine rich (SFPQ/PSF) is involved in influenza virus transcription. PLoS Pathog [Internet]. 2011;7(11):e1002397. Available from: 10.1371/journal.ppat.1002397. 2025 Nov 1. 10.1371/journal.ppat.1002397 PMC3219729 22114566

[48] Labeau A, Fery-Simonian L, Lefevre-Utile A, Pourcelot M, Bonnet-Madin L, Soumelis V, et al. Characterization and functional interrogation of the SARS-CoV-2 RNA interactome. Cell Rep [Internet]. 2022. https://pubmed.ncbi.nlm.nih.gov/35477000/. 2025 Nov 1. 10.1016/j.celrep.2022.110744 PMC9040432 35477000

[49] Wang G, Jiang L, Yan Y, Kong F, Li Q, Zhang J, et al. Cellular SLC35B4 promotes internalization during influenza A virus entry. Mbio [Internet]. 2025;16(5). 10.1128/mbio.00194-25. 2026 Mar 11. 10.1128/mbio.00194-25 PMC12077083 40130891

[50] Wang G, Jiang L, Wang J, Zhang J, Kong F, Li Q, et al. The G protein-coupled receptor FFAR2 promotes internalization during influenza a virus entry. J Virol [Internet]. 2020;94(2). 10.1128/JVI.01707-19. 2026 Mar 11. 10.1128/JVI.01707-19 PMC6955252 31694949

[51] Ang F, Wong APY, Ng MML, Chu JJH. Small interference RNA profiling reveals the essential role of human membrane trafficking genes in mediating the infectious entry of dengue virus. Virol J. 2010 [cited 2026 Mar 11];7:24–. Available from: 10.1186/1743-422X-7-24 10.1186/1743-422X-7-24 PMC2825209 20122152

[52] Krishnan MN, Sukumaran B, Pal U, Agaisse H, Murray JL, Hodge TW, et al. Rab 5 is required for the cellular entry of dengue and West Nile viruses. J Virol [Internet]. 2007;81(9):4881–85. Available from: 10.1128/JVI.02210-06. 2026 Mar 11. 17301152 10.1128/JVI.02210-06 PMC1900185

[53] Chen Y, Klute S, Sparrer KMJ, Serra-Moreno R. RAB5 is a host dependency factor for the generation of SARS-CoV-2 replication organelles. Mbio [Internet]. 2025. https://journals.asm.org/doi/pdf/10.1128/mbio.03314-24?download=true. 2025 Nov 24]. 10.1128/mbio.03314-24 PMC12077180 40167317

[54] G R, C L. Requirement for vacuolar proton-ATPase activity during entry of influenza virus into cells. J Virol [Internet]. 1995;69(4):2306–12. Available from: https://pubmed.ncbi.nlm.nih.gov/7884876/. 2026 Mar 11. 7884876 10.1128/jvi.69.4.2306-2312.1995 PMC188901

[55] Sabino C, Basic M, Bender D, Elgner F, Himmelsbach K, Hildt E. Bafilomycin A1 and U18666A efficiently impair ZIKV infection. Viruses [Internet]. 2019. https://pmc.ncbi.nlm.nih.gov/articles/PMC6630673/. 2026 Mar 11. 10.3390/v11060524 PMC6630673 31174294

[56] Zhang C, Min YQ, Xue H, Zhang H, Liu K, Tian Y, et al. Host protein ARF1 is a proviral factor for SARS-CoV-2 and a candidate broad-spectrum therapeutic target. Nat Commun, 161 [Internet]. 2025 [cited 2026 Mar 14] 16(1):6326–. Available from: https://www.nature.com/articles/s41467-025-61431-8. 10.1038/s41467-025-61431-8 PMC12241596 40634337

[57] Sun E, He J, Zhuang X. Dissecting the role of COPI complexes in influenza virus infection. J Virol [Internet]. 2013;87(5):2673–85. Available from: 10.1128/JVI.02277-12. 2026 Mar 14. 23255804 10.1128/JVI.02277-12 PMC3571408

[58] Watanabe K, Takizawa N, Katoh M, Hoshida K, Kobayashi N, Nagata K. Inhibition of nuclear export of ribonucleoprotein complexes of influenza virus by leptomycin B. Virus Res [Internet]. 2001;77(1):31–42. Available from: https://www.sciencedirect.com/science/article/abs/pii/S0168170201002635?via%3Dihub. 2026 Mar 14. 11451485 10.1016/s0168-1702(01)00263-5

[59] Kashyap T, Murray J, Walker CJ, Chang H, Tamir S, Hou B, et al. Selinexor, a novel selective inhibitor of nuclear export, reduces SARS-CoV-2 infection and protects the respiratory system in vivo. Antiviral res [Internet]. 2021 [cited 2026 Mar 14], 192, 105115. Available from: https://pmc.ncbi.nlm.nih.gov/articles/PMC8213878/. 10.1016/j.antiviral.2021.105115 PMC8213878 34157321

[60] Itoh Y, Miyamoto Y, Tokunaga M, Suzuki T, Takada A, Ninomiya A, et al. Importin-7-dependent nuclear translocation of the flavivirus core protein is required for infectious virus production. PLoS Pathog [Internet]. 2024;20(8):e1012409. Available from: 10.1371/journal.ppat.1012409. 2025 Oct 13. 10.1371/journal.ppat.1012409 PMC11326614 39146232

[61] Slaine PD, Kleer M, Smith NK, Khaperskyy DA, McCormick C. Stress granule-inducing eukaryotic translation initiation factor 4A inhibitors block influenza a virus replication. Viruses [Internet]. 2017;9(12):388. Available from: https://pmc.ncbi.nlm.nih.gov/articles/PMC5744162/. 2026 Mar 14. 29258238 10.3390/v9120388 PMC5744162

[62] Müller C, Obermann W, Karl N, Wendel HG, Taroncher-Oldenburg G, Pleschka S, et al. The rocaglate CR-31-B (−) inhibits SARS-CoV-2 replication at non-cytotoxic, low nanomolar concentrations in vitro and ex vivo. Antiviral Res [Internet]. 2021;186. Available from: 10.1016/j.antiviral.2021.105012. 2026 Mar 14. 33422611 10.1016/j.antiviral.2021.105012 PMC7791309

[63] Polacek C, Friebe P, Harris E. Poly(A)-binding protein binds to the non-polyadenylated 3′ untranslated region of dengue virus and modulates translation efficiency. J Gener Virol [Internet]. 2009;90(3):687–92. Available from: 10.1099/vir.0.007021-0. 2026 Mar 14. 10.1099/vir.0.007021-0 19218215

[64] Schmidt N, Lareau CA, Keshishian H, Ganskih S, Schneider C, Hennig T, et al. The SARS-CoV-2 RNA–protein interactome in infected human cells. Nat Microbiol, 63 [Internet]. 2020 [cited 2026 Mar 14]; 6(3):339–53. Available from: https://www.nature.com/articles/s41564-020-00846-z. 33349665 10.1038/s41564-020-00846-z PMC7906908

[65] Riva V, Garbelli A, Brai A, Casiraghi F, Fazi R, Trivisani CI, et al. Unique domain for a Unique target: selective inhibitors of host cell DDX3X to fight Emerging viruses. J Med Chem [Internet]. 2020;63(17):9876–87. Available from: 10.1021/acs.jmedchem.0c01039. 2026 Mar 14. 32787106 10.1021/acs.jmedchem.0c01039

[66] Vesuna F, Akhrymuk I, Smith A, Winnard PT, Lin S-C, Scharpf R, et al. RK-33, a small molecule inhibitor of host RNA helicase DDX3, suppresses multiple variants of SARS-CoV-2. bioRxiv prepr serv biol [Internet]. 2022. https://pubmed.ncbi.nlm.nih.gov/35262079/. 2026 Mar 14];. 10.3389/fmicb.2022.959577 PMC9453862 36090095

[67] Zheng Q, Hou J, Zhou Y, Li Z, Cao X. The RNA helicase DDX46 inhibits innate immunity by entrapping m6A-demethylated antiviral transcripts in the nucleus. Nat Immunol [Internet]. 2017;18(10):1094–103. Available from: https://pubmed.ncbi.nlm.nih.gov/28846086/. 2025 Oct 13. 28846086 10.1038/ni.3830

[68] Dechtawewat T, Songprakhon P, Limjindaporn T, Puttikhunt C, Kasinrerk W, Saitornuang S, et al. Role of human heterogeneous nuclear ribonucleoprotein C1/C2 in dengue virus replication. Virol J, 121 [Internet]. 2015 [cited 2026 Mar 14] 12(1):14–. Available from: 10.1186/s12985-014-0219-7. 10.1186/s12985-014-0219-7 PMC4351676 25890165

[69] Zhang X, Chu H, Chik KKH, Wen L, Shuai H, Yang D, et al. hnRNP C modulates MERS-CoV and SARS-CoV-2 replication by governing the expression of a subset of circRnas and cognitive mRNAs. Emerging Microbes Infections [Internet]. 2022;11(1):519. Available from: https://pmc.ncbi.nlm.nih.gov/articles/PMC8843244/. 2026 Mar 14. 35060842 10.1080/22221751.2022.2032372 PMC8843244

[70] Anderson DE, Cui J, Ye Q, Huang B, Tan Y, Jiang C, et al. Orthogonal genome-wide screens of bat cells identify MTHFD1 as a target of broad antiviral therapy. Proc Natl Acad Sci USA [Internet]. 2021;118(39):e2104759118. Available from: 10.1073/pnas.2104759118. 2026 Mar 14. 10.1073/pnas.2104759118 PMC8488669 34544865

[71] Sager G, Gabaglio S, Sztul E, Belov GA. Role of host cell secretory machinery in Zika virus life cycle. Viruses [Internet]. 2018;10(10):559. Available from: https://pmc.ncbi.nlm.nih.gov/articles/PMC6213159/. 2026 Mar 14. 30326556 10.3390/v10100559 PMC6213159

[72] Li J, Gui Q, Liang F-X, Sall J, Zhang Q, Duan Y, et al. The REEP5/TRAM1 complex binds SARS-CoV-2 NSP3 and promotes virus replication. J Virol [Internet]. 2023. https://journals.asm.org/doi/pdf/10.1128/jvi.00507-23?download=true. 2025 Nov 24. 10.1128/jvi.00507-23 PMC10617467 37768083

[73] Rao Y, Qin C, Espinosa B, Wang TY, Feng S, Savas AC, et al. Targeting CTP synthetase 1 to restore interferon induction and impede nucleotide synthesis in SARS-CoV-2 infection. Mbio [Internet]. 2025. https://pubmed.ncbi.nlm.nih.gov/40298378/. 2025 Oct 13. 10.1128/mbio.00649-25 PMC12153265 40298378

[74] Xu K, Klenk C, Liu B, Keiner B, Cheng J, Zheng B-J, et al. Modification of Nonstructural protein 1 of influenza a virus by SUMO1. J Virol [Internet]. 2011;85(2):1086–98. Available from: 10.1128/JVI.00877-10. 2026 Mar 14. 21047957 10.1128/JVI.00877-10 PMC3020006

[75] Fan Y, Li X, Zhang L, Zong Z, Wang F, Huang J, et al. Sumoylation in viral replication and antiviral defense. Adv Sci [Internet]. 2022;9(7):2104126. Available from: 10.1002/advs.202104126. 2026 Mar 14. 10.1002/advs.202104126 PMC8895153 35060688

[76] Moisy D, Avilov S V, Jacob Y, Laoide BM, Ge X, Baudin F, et al. HMGB1 protein binds to influenza virus nucleoprotein and promotes viral replication. J Virol [Internet]. 2012;86(17):9122–33. Available from: 10.1128/JVI.00789-12. 2026 Mar 14. 22696656 10.1128/JVI.00789-12 PMC3416134

[77] Savidis G, McDougall WM, Meraner P, Perreira JM, Portmann JM, Trincucci G, et al. Identification of Zika Virus and dengue virus dependency factors using functional Genomics. Cell Rep [Internet]. 2016;16(1):232–46. Available from: https://pubmed.ncbi.nlm.nih.gov/27342126/. 2025 Sep 9. 27342126 10.1016/j.celrep.2016.06.028

[78] Carney DS, Davies BA, Horazdovsky BF. Vps9 domain-containing proteins: activators of Rab5 GTPases from yeast to neurons. Trends Cell Biol [Internet]. 2006;16(1):27–35. Available from: https://www.cell.com/action/showFullText?pii=S0962892405002989. 2026 Mar 14. 16330212 10.1016/j.tcb.2005.11.001

[79] Tapescu I, Cherry S. DDX RNA helicases: key players in cellular homeostasis and innate antiviral immunity. J Virol [Internet]. 2024;98(10). 10.1128/jvi.00040-24. 2025 Nov 3];. 10.1128/jvi.00040-24 PMC11494928 39212449

[80] Fan Y, Sanyal S, Bruzzone R. Breaking bad: how viruses subvert the cell cycle. Front Cell Infect Microbiol. 2018;8:407205. 10.3389/fcimb.2018.00396. 10.3389/fcimb.2018.00396 PMC6252338 30510918

[81] Chaurushiya MS, Weitzman MD. Viral manipulation of DNA repair and cell cycle checkpoints. DNA Repair (Amst) [Internet]. 2009;8(9):1166. Available from: https://pmc.ncbi.nlm.nih.gov/articles/PMC2725192/. 2025 Nov 3. 19473887 10.1016/j.dnarep.2009.04.016 PMC2725192

[82] Puerta-Guardo H, Biering SB, de Sousa FTG, Shu J, Glasner DR, Li J, et al. Flavivirus NS1 triggers tissue-specific disassembly of intercellular Junctions leading to barrier dysfunction and Vascular leak in a GSK-3β-dependent manner. Pathogens. 2022 [cited 2025 Nov 23], 11, [Internet]. 615. Available from: https://www.mdpi.com/2076-0817/11/6/615/htm. 10.3390/pathogens11060615 PMC9228372 35745469

[83] Suwarto S, Sasmono RT, Sinto R, Ibrahim E, Suryamin M. Association of Endothelial Glycocalyx and tight and adherens junctions with severity of plasma leakage in dengue infection. The J Infect Dis [Internet]. 2017;215:992–99. Available from: 10.1093/infdis/jix041. 2025 Oct 11. 28453844 10.1093/infdis/jix041 PMC5407050

[84] Boudreault S, Roy P, Lemay G, Bisaillon M. Viral modulation of cellular RNA alternative splicing: a new key player in virus–host interactions? WIREs RNA [Internet]. 2019;10(5):e1543. Available from: https://pmc.ncbi.nlm.nih.gov/articles/PMC6767064/. 2025 Nov 23. 10.1002/wrna.1543 PMC6767064 31034770

[85] Lloyd RE. Regulation of stress granules and P-bodies during RNA virus infection. WIREs RNA [Internet]. 2013;4(3):317. Available from: https://pmc.ncbi.nlm.nih.gov/articles/PMC3652661/. 2025 Nov 23. 23554219 10.1002/wrna.1162 PMC3652661

[86] Kim J, Song CH. Stress granules in infectious disease: cellular principles and dynamic roles in immunity and organelles. Int J Mol Sci. 2024 [cited 2025 Nov 23];25:12950. Available from: https://www.mdpi.com/1422-0067/25/23/12950/htm. 10.3390/ijms252312950 PMC11641027 39684660

[87] Luo H. Interplay between the virus and the ubiquitin–proteasome system: molecular mechanism of viral pathogenesis. Curr Opin Virol [Internet]. 2016;17:1–10. Available from: https://www.sciencedirect.com/science/article/pii/S1879625715001406?utm_source=chatgpt.com. 2025 Nov 23. 26426962 10.1016/j.coviro.2015.09.005 PMC7102833

[88] Wang YE, Pernet O, Lee B. Regulation of the nucleocytoplasmic trafficking of viral and cellular proteins by ubiquitin and small ubiquitin-related modifiers. Biol Cell [Internet]. 2012;104(3):121. Available from: https://pmc.ncbi.nlm.nih.gov/articles/PMC3625690/. 2025 Nov 23. 22188262 10.1111/boc.201100105 PMC3625690

[89] Lu Z, Song G, Zhu H, Lei C, Sun X, Wang K, et al. DTIAM: a unified framework for predicting drug-target interactions, binding affinities and drug mechanisms. Nat Commun [Internet]. 2025;16(1):1–17. Available from: https://www.nature.com/articles/s41467-025-57828-0. 2025 Oct 13. 40089473 10.1038/s41467-025-57828-0 PMC11910601

[90] Schräder T, Dudek SE, Schreiber A, Ehrhardt C, Planz O, Ludwig S. The clinically approved MEK inhibitor trametinib efficiently blocks influenza a virus propagation and cytokine expression. Antiviral Res [Internet]. 2018;157:80–92. Available from: https://pubmed.ncbi.nlm.nih.gov/29990517/. 2025 Oct 13. 29990517 10.1016/j.antiviral.2018.07.006

[91] Valencia HJ, de Aguiar MCAM, Costa MA, Mendonça DC, Reis EV, Arias NEC, et al. Evaluation of kinase inhibitors as potential therapeutics for flavivirus infections. Arch Virol. 2021 [cited 2025 Oct 13];166:1433. Available from: https://pmc.ncbi.nlm.nih.gov/articles/PMC7938686. 10.1007/s00705-021-05021-1 PMC7938686 33683474

[92] de Wispelaere M, LaCroix AJ, Yang PL, de Wispelaere M. The small molecules AZD0530 and dasatinib inhibit dengue virus RNA replication via fyn kinase. J Virol [Internet]. 2013;87(13):7367. Available from: https://pmc.ncbi.nlm.nih.gov/articles/PMC3700292/. 2025 Oct 13. 10.1128/JVI.00632-13 PMC3700292 23616652

[93] Chu JJH, Yang PL. C-src protein kinase inhibitors block assembly and maturation of dengue virus. Proc Natl Acad Sci USA [Internet]. 2007;104(9):3520–25. Available from: 10.1073/pnas.0611681104. 2025 Oct 13. 17360676 10.1073/pnas.0611681104 PMC1805510

[94] Yang L, Pei R-J, Li H, Ma X-N, Zhou Y, Zhu F-H, et al. Identification of SARS-CoV-2 entry inhibitors among already approved drugs. Acta Pharmacol Sin [Internet]. 2021;42(8):1347–53. Available from: https://www.nature.com/articles/s41401-020-00556-6. 2025 Oct 13. 33116249 10.1038/s41401-020-00556-6 PMC7594953

[95] Strobelt R, Adler J, Paran N, Yahalom-Ronen Y, Melamed S, Politi B, et al. Imatinib inhibits SARS-CoV-2 infection by an off-target-mechanism. Sci Rep [Internet]. 2022;12(1):1–11. Available from: https://www.nature.com/articles/s41598-022-09664-1. 2025 Oct 13. 35388061 10.1038/s41598-022-09664-1 PMC8984672

[96] Carreño-Flórez GP, Cuartas-López AM, Boudreau RL, Vicente-Manzanares M, Gallego-Gómez JC. Role of c-ABL in DENV-2 infection and actin remodeling in Vero cells. Int J Mol Sci. 2025 [cited 2025 Oct 13];26:4206. Available from: https://www.mdpi.com/1422-0067/26/9/4206/htm. 10.3390/ijms26094206 PMC12071696 40362443

[97] Touret F, Driouich JS, Cochin M, Petit PR, Gilles M, Barthélémy K, et al. Preclinical evaluation of imatinib does not support its use as an antiviral drug against SARS-CoV-2. Antiviral Res [Internet]. 2021;193:105137. Available from: https://pmc.ncbi.nlm.nih.gov/articles/PMC8274277/. 2025 Oct 13. 34265358 10.1016/j.antiviral.2021.105137 PMC8274277

[98] Duran A, Valero N, Mosquera J, Fuenmayor E, Alvarez-Mon M. Gefitinib and pyrrolidine dithiocarbamate decrease viral replication and cytokine production in dengue virus infected human monocyte cultures. Life Sci [Internet]. 2017;191:180–85. Available from: https://www.sciencedirect.com/science/article/abs/pii/S0024320517305416?via%3Dihub. 2025 Oct 13. 29055802 10.1016/j.lfs.2017.10.027

[99] Han Y, Kim S, Park T, Hwang H, Park S, Kim J, et al. Reduction of severe acute respiratory syndrome coronavirus 2 (SARS-CoV-2) variant infection by blocking the epidermal growth factor receptor (EGFR) pathway. Microbiol. Spectr [Internet]. 2024;12(11). 10.1128/spectrum.01583-24. 2025 Oct 13];. 10.1128/spectrum.01583-24 PMC11537080 39291996

[100] Xiao X, Wang C, Chang D, Wang Y, Dong X, Jiao T, et al. Identification of potent and safe antiviral therapeutic candidates against SARS-CoV-2. Front Immunol [Internet]. 2020;11:586572. Available from: https://pmc.ncbi.nlm.nih.gov/articles/PMC7723961/. 2025 Oct 13. 33324406 10.3389/fimmu.2020.586572 PMC7723961

[101] Lin X, Ke X, Jian X, Xia L, Yang Y, Zhang T, et al. Azacytidine targeting SARS-CoV-2 viral RNA as a potential treatment for COVID-19. Sci Bull. 2022 [cited 2025 Oct 13];67:1022. Available from: https://pmc.ncbi.nlm.nih.gov/articles/PMC8837489/10.1016/j.scib.2022.02.002 PMC8837489 35186363

[102] Eyr NS, Kirb EN, Anfiteatr DR, Bracho G, Russ AG, Whit PA, et al. Identification of estrogen receptor modulators as inhibitors of flavivirus infection. Antimicrob agents chemother [Internet]. 2020. https://pubmed.ncbi.nlm.nih.gov/32482672/. 2025 Oct 13. 10.1128/AAC.00289-20 PMC7526829 32482672

[103] Estrogen protects women against the flu, study finds | infection control today [Internet]. [ https://www.infectioncontroltoday.com/view/estrogen-protects-women-against-flu-study-finds. 2025 Dec 8].

[104] Iaconis D, Bordi L, Matusali G, Talarico C, Manelfi C, Cesta MC, et al. Characterization of raloxifene as a potential pharmacological agent against SARS-CoV-2 and its variants. Cell death dis [Internet]. 2022. https://pubmed.ncbi.nlm.nih.gov/35614039/. 2025 Oct 13]. 10.1038/s41419-022-04961-z PMC9130985 35614039

[105] Bocci G, Bradfute SB, Ye C, Garcia MJ, Parvathareddy J, Reichard W, et al. Virtual and in vitro antiviral screening revive therapeutic drugs for COVID-19. ACS Pharmacol Transl Sci [Internet]. 2020;3(6):1278–92. Available from: 10.1021/acsptsci.0c00131. 2025 Oct 13. 33330842 10.1021/acsptsci.0c00131 PMC7571299

[106] LoMascolo NJ, Cruz-Pulido YE, Mounce BC. Bisacodyl limits Chikungunya virus replication in vitro and is broadly antiviral. Antimicrob agents chemother [Internet]. 2022. https://pubmed.ncbi.nlm.nih.gov/35652314/. 2025 Oct 13. 10.1128/aac.00292-22 PMC9211418 35652314

[107] Peden AA, Oorschot V, Hesser BA, Austin CD, Scheller RH, Klumperman J. Localization of the AP-3 adaptor complex defines a novel endosomal exit site for lysosomal membrane proteins. J Cell Biol [Internet]. 2004;164(7):1065–76. Available from: https://pubmed.ncbi.nlm.nih.gov/15051738/. 2025 Nov 24. 15051738 10.1083/jcb.200311064 PMC2172074

[108] Van Der Schaar HM, Rust MJ, Chen C, van der Ende-Metselaar H, Wilschut J, Zhuang X, et al. Dissecting the cell entry pathway of dengue virus by single-particle tracking in living cells. PLoS Pathog [Internet]. 2008;4(12):e1000244. Available from: 10.1371/journal.ppat.1000244. 2025 Nov 24. 10.1371/journal.ppat.1000244 PMC2592694 19096510

[109] Puerta-Guardo H, Glasner DR, Espinosa DA, Biering SB, Patana M, Ratnasiri K, et al. Flavivirus NS1 triggers tissue-specific vascular endothelial dysfunction reflecting disease tropism. Cell Rep [Internet]. 2019;26(6):1598–613.e8. Available from: https://pubmed.ncbi.nlm.nih.gov/30726741/. 2025 Nov 3. 30726741 10.1016/j.celrep.2019.01.036 PMC6934102

[110] Brass AL, Huang IC, Benita Y, John SP, Krishnan MN, Feeley EM, et al. The IFITM proteins mediate cellular resistance to influenza a H1N1 virus, West Nile virus, and dengue virus. Cell [Internet]. 2009;139(7):1243–54. Available from: 10.1016/j.cell.2009.12.017. 20064371 10.1016/j.cell.2009.12.017 PMC2824905

[111] Huang S, Chen J, Chen Q, Wang H, Yao Y, Chen J, et al. A second CRM1-dependent nuclear export signal in the influenza a virus NS2 protein contributes to the nuclear export of viral ribonucleoproteins. J Virol [Internet]. 2013;87(2):767. Available from: https://pmc.ncbi.nlm.nih.gov/articles/PMC3554077/. 2025 Oct 13. 23115280 10.1128/JVI.06519-11 PMC3554077

[112] Tsai PL, Chiou NT, Kuss S, García-Sastre A, Lynch KW, Fontoura BMA. Cellular RNA binding proteins NS1-BP and hnRNP K regulate influenza a virus RNA splicing. PLoS pathog [Internet]. 2013. https://pubmed.ncbi.nlm.nih.gov/23825951/. 2025 Oct 13];. 10.1371/journal.ppat.1003460 PMC3694860 23825951

[113] Firdaus MER, Dukhno E, Kapoor R, Gerlach P. Two birds with one stone: RNA virus strategies to manipulate G3BP1 and other stress granule components. WIREs RNA [Internet]. 2025;16(2):e70005. Available from: https://pmc.ncbi.nlm.nih.gov/articles/PMC11962251/. 2025 Oct 13. 10.1002/wrna.70005 PMC11962251 40170442

[114] Han Q, Chang C, Li L, Klenk C, Cheng J, Chen Y, et al. Sumoylation of influenza a virus nucleoprotein is essential for intracellular trafficking and virus growth. J Virol [Internet]. 2014;88(16):9379–90. Available from: https://pubmed.ncbi.nlm.nih.gov/24920808/. 2025 Nov 24. 24920808 10.1128/JVI.00509-14 PMC4136286

[115] Su C-I, Tseng C-H, Yu C-Y, Lai MMC, Jung JU. SUMO Modification stabilizes dengue virus nonstructural protein 5 to support virus replication. J Virol [Internet]. 2016;90(9):4308–19. Available from: 10.1128/JVI.00223-16. 2025 Nov 24. 26889037 10.1128/JVI.00223-16 PMC4836324

[116] Ren J, Wang S, Zong Z, Pan T, Liu S, Mao W, et al. TRIM28-mediated nucleocapsid protein SUMOylation enhances SARS-CoV-2 virulence. Nat Commun, 151 [Internet]. 2024 [cited 2025 Nov 24], 15(1):244–. Available from: https://www.nature.com/articles/s41467-023-44502-6. 10.1038/s41467-023-44502-6 PMC10764958 38172120

[117] Zhou D, Wu Z, Park JG, Fiches GN, Li TW, Ma Q, et al. FACT subunit SUPT16H associates with BRD4 and contributes to silencing of interferon signaling. Nucleic Acids Res [Internet]. 2022;50(15):8700–18. Available from: https://pubmed.ncbi.nlm.nih.gov/35904816/. 2025 Nov 24. 35904816 10.1093/nar/gkac645 PMC9410884

[118] Dong Y, Ming B, Dong L. The role of HMGB1 in rheumatic diseases. Front immunol [Internet]. 2022. https://pubmed.ncbi.nlm.nih.gov/35250993/. 2025 Nov 24. 10.3389/fimmu.2022.815257 PMC8892237 35250993

[119] Yang J, Tian B, Wang P, Chen R, Xiao K, Long X, et al. SARS-CoV-2 NSP3/4 control formation of replication organelle and recruitment of RNA polymerase NSP12. J cell biol [Internet]. 2025. https://pubmed.ncbi.nlm.nih.gov/39737877/. 2025 Nov 24];. 10.1083/jcb.202306101 PMC11687299 39737877

[120] Fang J, Pietzsch C, Ramanathan P, Santos RI, Ilinykh PA, Garcia-Blanco MA, et al. Staufen1 interacts with multiple components of the Ebola virus ribonucleoprotein and enhances viral RNA synthesis. Mbio [Internet]. 2018. https://pubmed.ncbi.nlm.nih.gov/30301857/. 2025 Sep 10. 10.1128/mBio.01771-18 PMC6178623 30301857

[121] Rivera A, Messaoudi I. Molecular mechanisms of Ebola pathogenesis. J Leukoc Biol [Internet]. 2016;100(5):889. Available from: https://pmc.ncbi.nlm.nih.gov/articles/PMC6608070/. 2025 Oct 12. 27587404 10.1189/jlb.4RI0316-099RR PMC6608070

[122] Spurgers KB, Alefantis T, Peyser BD, Ruthel GT, Bergeron AA, Costantino JA, et al. Identification of essential filovirion-associated host factors by serial Proteomic analysis and RNAi screen. Mol Cellular Proteomics [Internet]. 2010;9(12):2690. Available from: https://pmc.ncbi.nlm.nih.gov/articles/PMC3101857/. 2025 Oct 12. 10.1074/mcp.M110.003418 PMC3101857 20702783

[123] Smith DR, McCarthy S, Chrovian A, Olinger G, Stossel A, Geisbert TW, et al. Inhibition of heat-shock protein 90 reduces Ebola virus replication. Antiviral Res [Internet]. 2010;87(2):187–94. Available from: https://pubmed.ncbi.nlm.nih.gov/20452380/. 2025 Oct 12. 20452380 10.1016/j.antiviral.2010.04.015 PMC2907434

[124] Fang J, Pietzsch C, Tsaprailis G, Crynen G, Cho KF, Ting AY, et al. Functional interactomes of the Ebola virus polymerase identified by proximity proteomics in the context of viral replication. Cell Rep [Internet]. 2022;38(12):110544. Available from: https://pmc.ncbi.nlm.nih.gov/articles/PMC10496643/. 2025 Oct 12. 35320713 10.1016/j.celrep.2022.110544 PMC10496643

[125] Bray M, Driscoll J, Huggins JW. Treatment of lethal Ebola virus infection in mice with a single dose of an S-adenosyl-L-homocysteine hydrolase inhibitor. Antiviral Res [Internet]. 2000;45(2):135–47. Available from: https://pubmed.ncbi.nlm.nih.gov/10809022/. 2025 Oct 12. 10809022 10.1016/s0166-3542(00)00066-8

[126] Spence JS, Krause TB, Mittler E, Jangra RK, Chandran K. Direct visualization of Ebola virus fusion triggering in the endocytic pathway. Mbio [Internet]. 2016. https://pubmed.ncbi.nlm.nih.gov/26861015/. 2025 Oct 12. 10.1128/mBio.01857-15 PMC4752599 26861015

[127] Ludwig S, Pleschka S, Planz O. MEK inhibitors as novel host-targeted antivirals with a dual-benefit mode of action against hyperinflammatory respiratory viral diseases. Curr Opin Virol [Internet]. 2023;59:101304. Available from: https://www.sciencedirect.com/science/article/pii/S1879625723000044?utm_source=chatgpt.com. 2025 Oct 13. 36841033 10.1016/j.coviro.2023.101304 PMC10091867

[128] Hajjo R, Sabbah DA, Abusara OH, Kharmah R, Bardaweel S. Targeting human proteins for antiviral drug discovery and repurposing efforts: a focus on protein kinases. Viruses. 2023 [cited 2025 Nov 3];15:568. Available from: https://www.mdpi.com/1999-4915/15/2/568/htm 10.3390/v15020568 PMC9966946 36851782

[129] O’Donovan SM, Imami A, Eby H, Henkel ND, Creeden JF, Asah S, et al. Identification of candidate repurposable drugs to combat COVID-19 using a signature-based approach. Sci Rep. 2021 [cited 2025 Nov 3];11:1–12. Available from: https://www.nature.com/articles/s41598-021-84044-9 10.1038/s41598-021-84044-9 PMC7904823 33627767

