# Supplementary figures and images for: A pan-viral map of host dependency factors from multi-omics integration and machine learning across influenza A, SARS-CoV-2, Zika, and dengue viruses
Source: J Transl Med. 2026 May 2;24:685. doi: 10.1186/s12967-026-08197-9 (PMC13188342; doi:10.1186/s12967-026-08197-9)

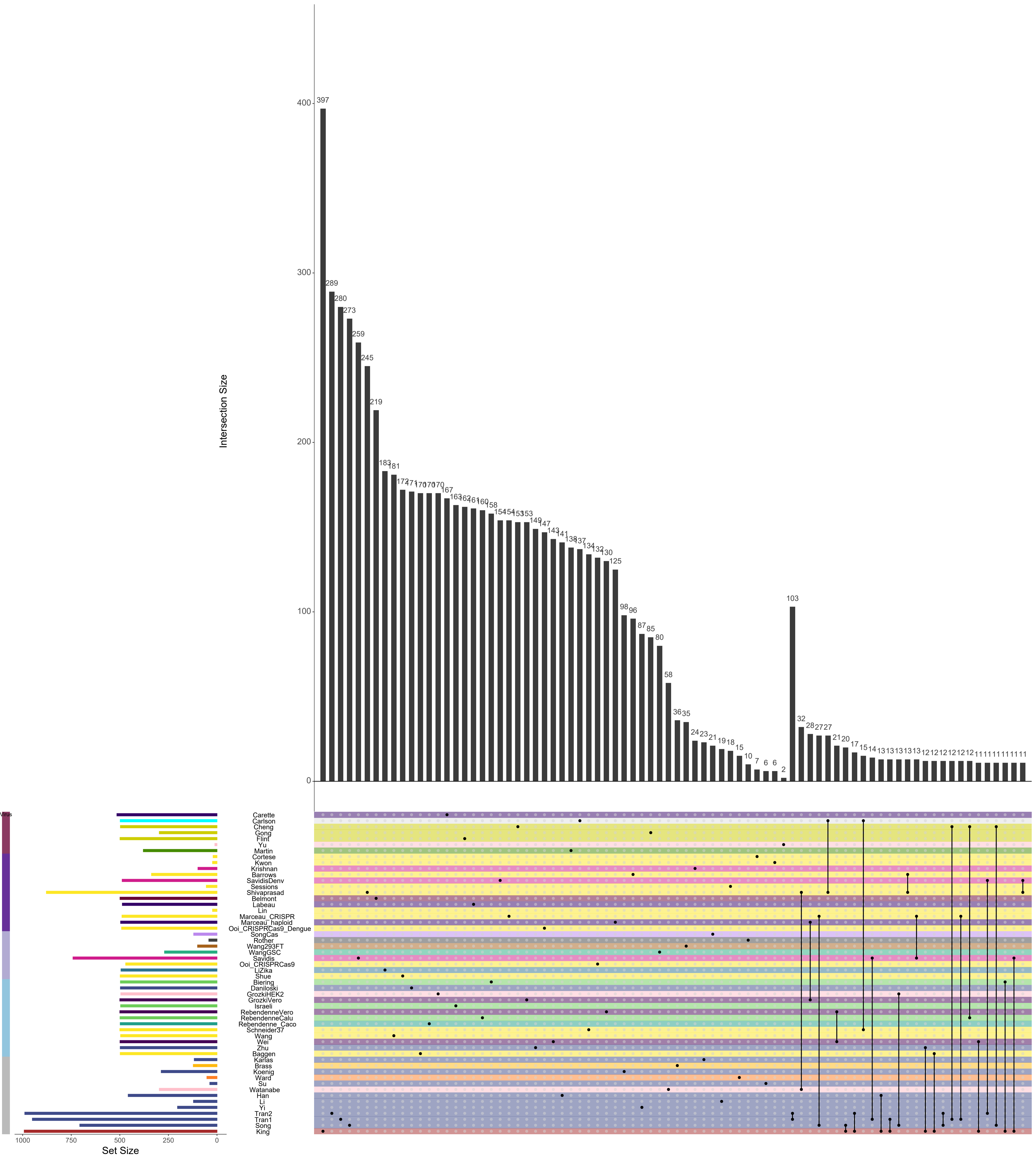

Supplement: Supplementary file 1 — Supplementary Material 1 [file 12967_2026_8197_MOESM1_ESM.pdf]
