# Supplementary material for: A pan-viral map of host dependency factors from multi-omics integration and machine learning across influenza A, SARS-CoV-2, Zika, and dengue viruses
Source: J Transl Med. 2026 May 2;24:685. doi: 10.1186/s12967-026-08197-9 (PMC13188342; doi:10.1186/s12967-026-08197-9)
Supplement: Supplementary file 3 — Supplementary Material 3 [file 12967_2026_8197_MOESM3_ESM.docx]

**A pan-viral map of host dependency factors from multi-omics integration and machine learning across Influenza A, SARS-CoV-2, Zika, and Dengue viruses**

Mohadeseh Naseri^1^, Alicia Hiemisch^1^, André Dietz^1^, Marcus Oswald^1^, Rainer König^1*^

^1^Institute for Infectious Diseases and Infection Control (IIMK), University Hospital, Jena, Germany

**Supplementary Material**

Content

[**Supplementary Figures** 2](#_Toc215586590)

[**Supplementary Tables** 9](#_Toc215586591)

[**Supplementary Text** 17](#_Toc215586592)

# **Supplementary Figures**

**Identification of studies via databases and registers**

Records removed *before screening*:

Duplicate records removed

(n = 884)

Records marked as ineligible by automation tools (n = 0)

Records removed for other reasons

(n = 0)

Records (n =2025) identified from: PubMed

(n = 602)

Web of Science

(n = 626)

Scopus

(n = 797)

**Identification**

Records excluded (Relevance based on abstract/title)

(n =1015)

Records screened

(n =1141)

Reports not retrieved

(n = 0)

Reports sought for retrieval

(n =126)

**Screening**

Reports excluded:

Data not available

(n = 11)

Reports assessed for eligibility

(n = 126)

Studies included in meta-analysis

(n =115)

Reports of included studies

(n = 0)

**Included**

**Figure S1:** PRISMA 2020 flow diagram for our systematic literature and database search to find data from Crispr/Cas9 or siRNA knockdown HDF screens, viral-protein – host-protein and viral-RNA – host-protein interaction screens, and single cell sequencing data of viral infected cells. The review included searches in PubMed, Web of Science, and the Scopus database. No automation tools were used; duplicates were removed by exact-match de-duplication on DOI/PMID/title. Full-text exclusions were labeled with explicit reasons, including: (i) wrong pathogen (not SARS-CoV-2, IAV, ZIKV, DENV, or EBOV), (ii) non-mammalian host, (iii) not a primary experimental study (review/editorial), (iv) not genome- or proteome-wide / no gene-level HDF output, (v) transcriptomic/proteomic data without gene list, (vi) cell-free systems only, and (vii) inaccessible or insufficient metadata. Exact record counts for each box are displayed in the diagram.


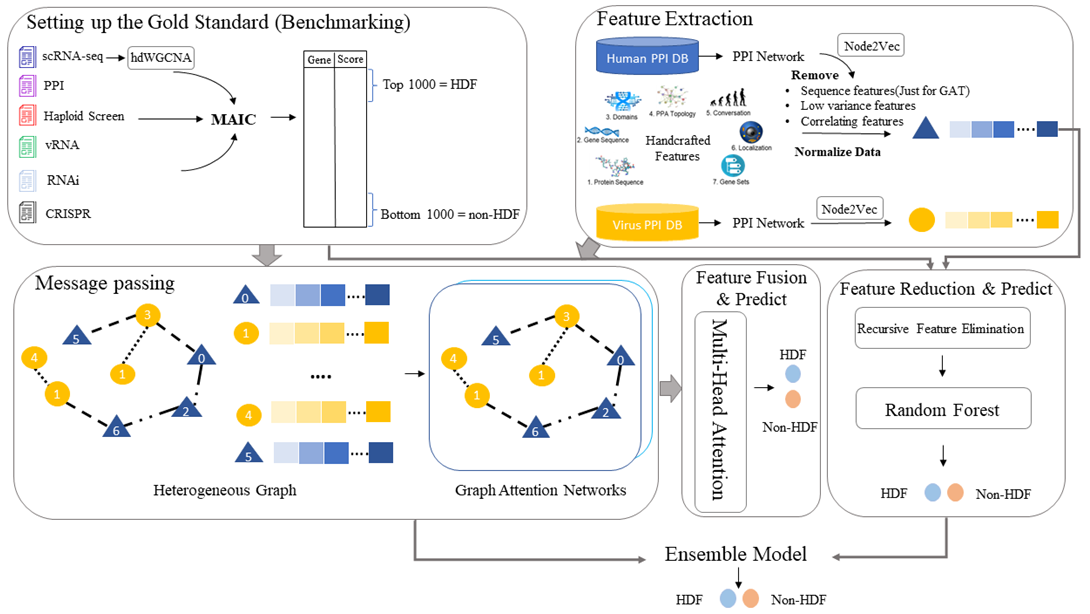


**Figure S2:** Detailed workflow including benchmarking setup, feature extraction, graph construction, and model training. This figure provides an expanded, method-centric view of the pipeline shown in Fig. 1.


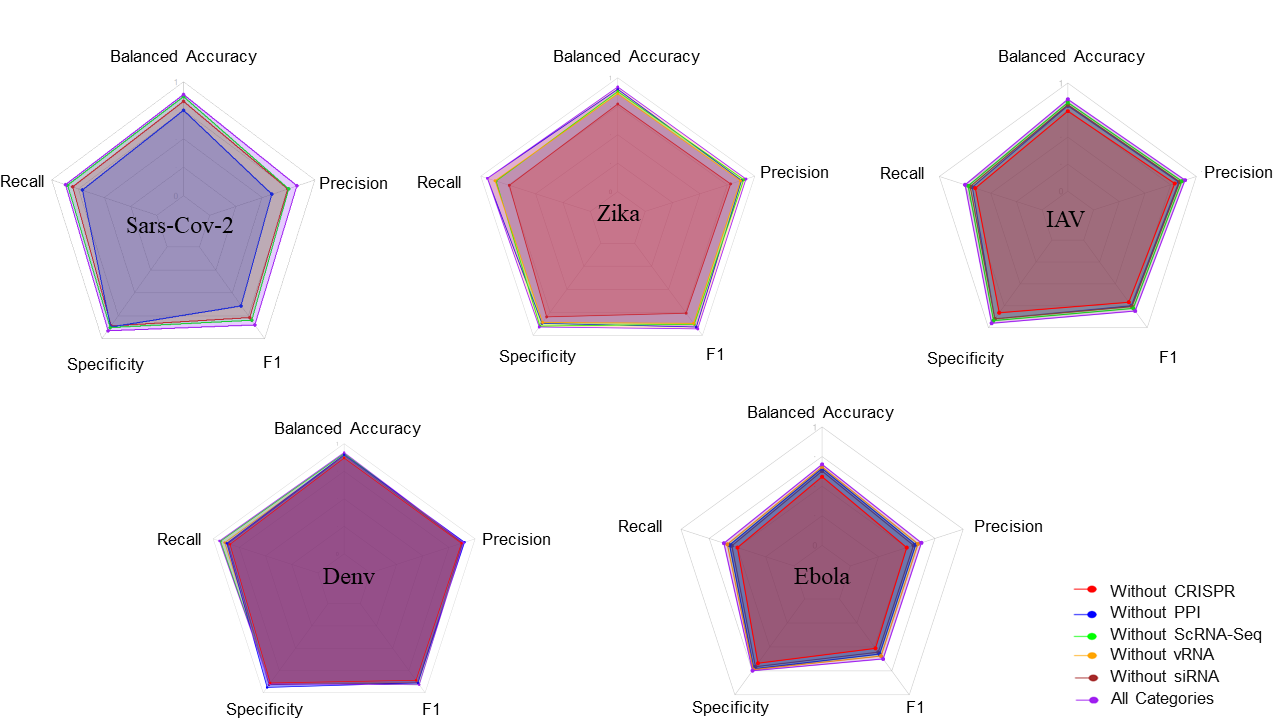


**Figure S3.** Performance results after leave-one-category-out ablation of the benchmarking data used to learn and test our machine learning models by MAIC across the studied five viruses. For each virus, we recomputed the MAIC consensus labels using all evidence sources except one category at a time (i.e., all data but CRISPR knockout, all data but PPI, all data but scRNA-seq, all data but vRNA, or all data but siRNA knockdown). We then retrained the models with the same cross-validation protocol used in the main analysis. Radar plots show mean outer-test balanced accuracy, precision, recall/sensitivity, specificity, and the F1-score. Larger areas indicate better performance. The “all categories” condition (full model, purple) performed best. Removing PPI caused the largest degradation for SARS-CoV-2, whereas removing CRISPR knockout data had the greatest impact on the performances for IAV, ZIKV, DENV, and EBOV.

**
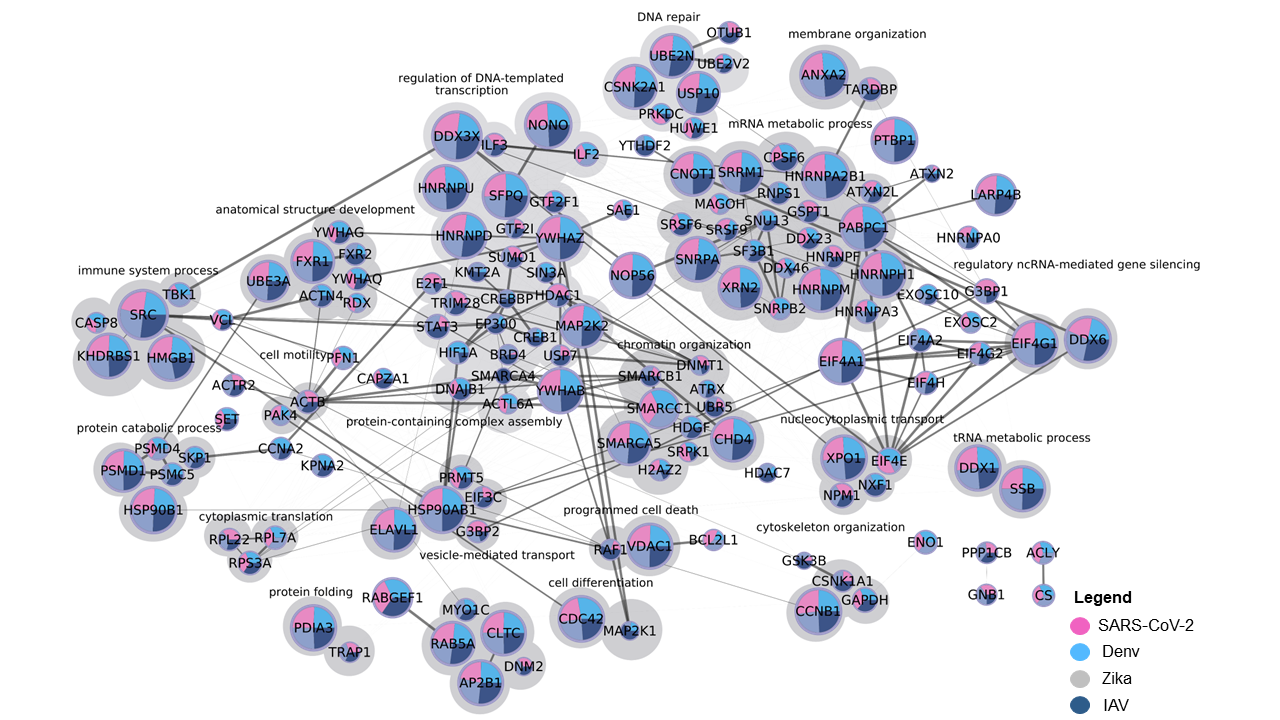
**

**Figure S4:** Nodes (n = 148) are host proteins from the prioritized predicted HDF list; edges were derived from STRING (v12 physical/experimental links, combined score ≥ 0.90). For clarity, only the top-8 highest-weight edges per node are shown. The edge width scales with the interaction weight from STRING. The node size is proportional to the global cross-virus prioritization score (final score). The inner four-slice pie encodes virus-specific normalized ranks (IAV, SARS-CoV-2, DENV, ZIKV). Similar biological processes are displayed, surrounded by a gray ring. Dominant communities collapsed into modules related to RNA processing/spliceosome, mRNA translation/initiation (eIF4F hub), proteasome/chaperone machinery, and nuclear transport, indicating a shared cellular backbone exploited by multiple viruses. Among the factors influenced by three viruses, drug repurposing based on DTIAM identified pyrimethamine (CCNA2), vorinostat (HDAC1), dasatinib (MAP2K1), and nilotinib (RAF1) as the leading candidates.

Details concerning the method: using 210 prioritized host genes, we assembled a high-confidence interaction map based on STRING interaction definitions (v12 human links (physical/experimental evidence, combined score ≥ 0.90). 62 genes were excluded, 44 of them linked only to nodes outside the prioritization list, 17 of them lacked high-confidence physical/complex links, and one had an unresolved alias.

**
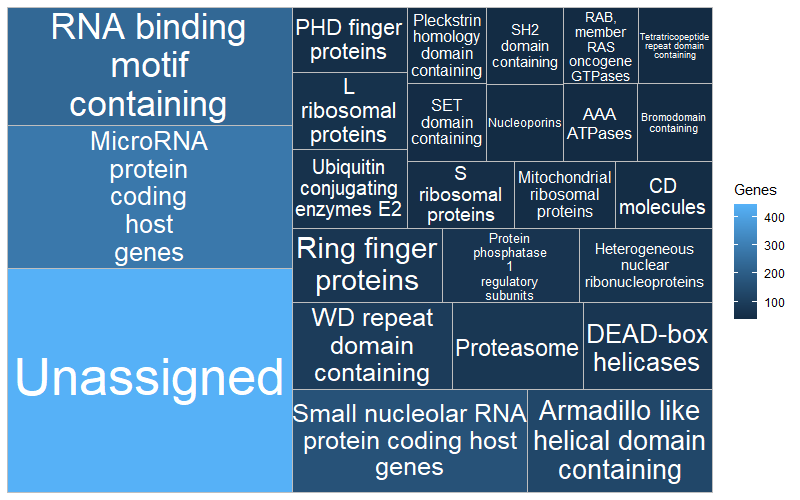
**

**Figure S5.** Gene-family composition of prioritized host genes (HGNC/PANTHER). Treemap of the top 25 gene families among the prioritized host genes aggregated across SARS-CoV-2, IAV, ZIKV, and DENV. Each rectangle is one family and its area (and fill) is proportional to the number of unique genes annotated to that family across any of the four viruses. Family names follow HGNC gene group nomenclature; when an HGNC family was unavailable, a PANTHER family/class was used and is prefixed “PANTHER:”. Because some genes belong to multiple families, such genes contribute to each of their families (membership counts). The category “Unassigned” includes genes without an HGNC or PANTHER family.


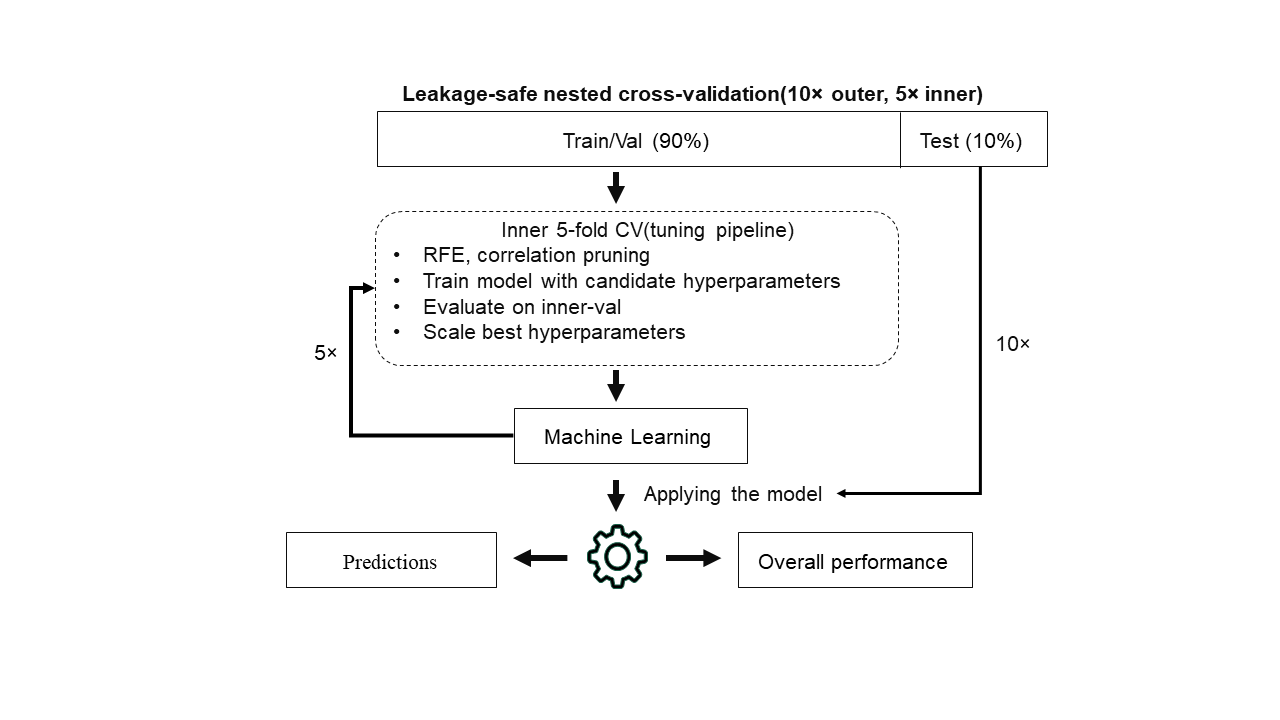


**Figure S6.** Leakage-safe nested cross-validation (CV) and model selection. For each virus, we run a 10× outer / 5× inner CV. In every outer fold the data were split into 90% training and 10% test data. Within the training split, a 5-fold inner CV was performed for all pre-processing and tuning steps: feature selection (RFE and filtering highly correlating features by removing features with |r|>0.70 to another feature, when two features were strongly correlated, we discarded the one showing the weaker association with the target variable), and hyperparameter search for RF, XGBoost, and HAN. The best inner-CV setting is re-fit on the full training data and evaluated once by the untouched test set to produce predictions and performance values. This process was 10 times repeated. Outer-fold results were averaged to report the mean performance values and their standard deviations.


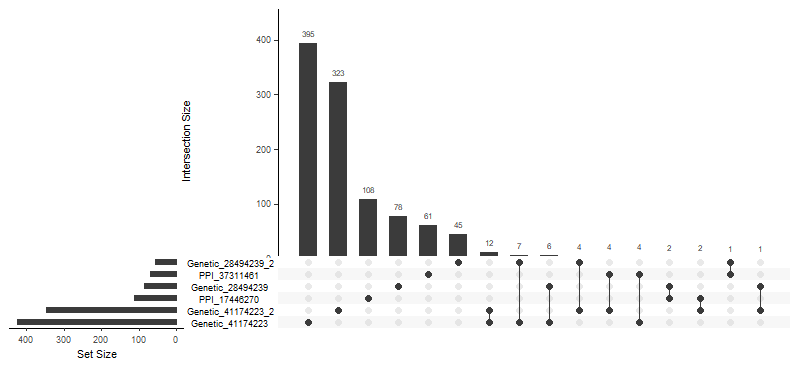


**Figure S7.** UpSet plot illustrating gene-level overlap among the six retained EBV evidence lists used in the exploratory oncogenic-virus benchmark. The benchmark included four genetic hit lists derived from CRISPR-based studies and two virus–host protein interaction datasets. Vertical bars represent the size of each intersection, horizontal bars indicate the size of each individual evidence list, and connected filled dots mark the datasets contributing to each intersection. No gene was shared by three or more retained evidence lists, indicating low agreement across the EBV evidence layers.

# **Supplementary Tables**

**Table S1**. Number of datasets used to construct the gold standards*

| Name of the virus | Genetic | PPI | Transcriptome | RNA-centric |
| --- | --- | --- | --- | --- |
| SARS-CoV-2 | 27 | 11 | 2 | 2 |
| Zika | 8 | 9 | 1 | 3 |
| Ebola | 7 | 4 | 0 | 2 |
| Denv | 14 | 16 | 1 | 4 |
| IAV | 18 | 16 | 1 | 0 |

*The numbers displayed are the numbers of the finally selected gene lists; **Genetic** = CRISPR-Cas9 knockout and/or RNAi knock-down screens; **PPI** = experimentally determined virus–host protein–protein interactions; **Transcriptome** = single-cell RNA-sequencing data; **RNA-centric** = RNA–protein interaction datasets (e.g., CLIP-seq/iCLIP/PAR-CLIP, RIP-seq). The displayed numbers are sums of ranked and unranked lists. When a single study reported multiple lists (e.g., different strains, MOIs, or cell lines), each list was counted and treated separately as input for MAIC.

**Table S2**. ZIKV data sources used to construct the MAIC gold standard

| **Category** | **Authors** | **Type** | **Year** | **Ref** |
| --- | --- | --- | --- | --- |
| vRNA–host protein | Zhang *et al.* | RANKED | 2021 | [1] |
|  | Ooi *et al.* | RANKED | 2019 | [2] |
| vRNA–host RNA | Liao *et al.* | RANKED | 2023 | [3] |
| CRISPR | Shue *et al.* | RANKED | 2021 | [4] |
|  | Li *et al.* | RANKED | 2019 | [5] |
|  | Savidis *et al.* | Unranked | 2016 | [6] |
|  | Wang *et al*. (GSC, 293FT) | RANKED | 2020 | [7] |
|  | Rother *et al.* | RANKED | 2021 | [8] |
|  | Ooi *et al.* | RANKED | 2019 | [2] |
| RNAi | Song *et al.* | Unranked | 2021 | [9] |
| Protein interaction | Shah *et al.* | Unranked | 2019 | [10] |
|  | Scaturro *et al.* | Unranked | 2018 | [11] |
|  | Persson *et al.* | RANKED | 2021 | [12] |
|  | Song *et al.* | Unranked | 2021 | [9] |
|  | Coyaud *et al.* | Unranked | 2018 | [13] |
|  | Kovanich *et al.* | Unranked | 2019 | [14] |
|  | Golubeva *et al.* | RANKED | 2020 | [15] |
|  | Zeng *et al.* | Unranked | 2020 | [16] |
| SingleCell | Zanini *et al.* | RANKED | 2018 | [17] |

**Table S3**. DENV data sources used to construct the MAIC gold standard

| **Category** | **Authors** | **Type** | **Year** | **Ref** |
| --- | --- | --- | --- | --- |
| vRNA–host protein | Viktorovskaya *et al.* | Unranked | 2016 | [18] |
|  | Ooi *et al.* | RANKED | 2019 | [2] |
|  | Phillips *et al.* | Unranked | 2016 | [19] |
| vRNA–host RNA | Liao *et al.* | RANKED | 2023 | [3] |
| CRISPR | Marceau *et al.* | RANKED | 2016 | [20] |
|  | Lin *et al.* | Unranked | 2017 | [21] |
|  | Ooi *et al.* | RANKED | 2019 | [2] |
|  | Belmont *et al.* | RANKED | 2024 | [22] |
|  | Shivaprasad *et al*. | RANKED | 2024 | [23] |
|  | Cheng *et al.* | RANKED | 2025 | [24] |
|  | Labeau *et al.* | RANKED | 2020 | [25] |
| RNAi | Sessions *et al.* | Unranked | 2009 | [26] |
|  | Savidis *et al.* | RANKED | 2016 | [6] |
|  | Barrows *et al.* | Unranked | 2019 | [27] |
|  | Krishnan *et al.* | Unranked | 2008 | [28] |
|  | Kwon *et al.* | Unranked | 2014 | [29] |
|  | Cortese *et al.* | RANKED | 2019 | [30] |
| Protein interaction | Khadka *et al.* | Unranked | 2011 | [31] |
|  | Hafirassou *et al.* | Unranked | 2017 | [32] |
|  | Maio *et al.* | Unranked | 2016 | [33] |
|  | Mairiang *et al.* | Unranked | 2013 | [34] |
|  | Silva *et al.* | Unranked | 2013 | [35] |
|  | Cervantes-Salazar *et al.* | Unranked | 2015 | [36] |
|  | Dechtawewat *et al.* | Unranked | 2016 | [37] |
|  | Silva *et al.* | Unranked | 2019 | [38] |
|  | Carpp *et al.* | Unranked | 2014 | [39] |
|  | Breton *et al.* | Unranked | 2011 | [40] |
|  | Poyomtip *et al.* | Unranked | 2016 | [41] |
|  | Ward *et al.* | Unranked | 2016 | [42] |
|  | Rabelo *et al.* | Unranked | 2017 | [43] |
|  | Brugier *et al.* | Unranked | 2022 | [44] |
|  | Jansen *et al.* | Unranked | 2021 | [45] |
|  | Shah *et al.* | Unranked | 2018 | [10] |
| SingleCell | Zanini *et al.* | RANKED | 2018 | [17] |

**Table S4**. IAV data sources used to construct the MAIC gold standard

| **Category** | **Authors** | **Type** | **Year** | **Ref** |
| --- | --- | --- | --- | --- |
| CRISPR | Han *et al*. (Rd2, Rd5) | RANKED | 2018 | [46] |
|  | Li *et al.* | RANKED | 2020 | [47] |
|  | Tran1 *et al.* (2 screens) | RANKED | 2020 | [48] |
|  | Yi *et al.* | RANKED | 2022 | [49] |
|  | Song *et al.* (3 screens) | Unranked | 2021 | [50] |
|  | King *et al.* | RANKED | 2023 | [51] |
| RNAi_confirmed | Su *et al.* | Unranked | 2013 | [52] |
|  | Tran *et al.* | Unranked | 2013 | [53] |
|  | Shapira *et al.* | Unranked | 2009 | [54] |
|  | Watanabe *et al.* | Unranked | 2014 | [55] |
|  | Haas *et al.* | Unranked | 2023 | [56] |
| RNAi | Brass *et al.* | RANKED | 2009 | [57] |
|  | Karlas *et al.* | RANKED | 2010 | [58] |
|  | Konig *et al.* | RANKED | 2010 | [59] |
|  | Ward *et al.* | RANKED | 2012 | [60] |
|  | Tripathi *et al.* | RANKED | 2015 | [61] |
| Protein interaction | Shapira *et al.* | Unranked | 2009 | [54] |
|  | Chassey *et al.* | Unranked | 2013 | [62] |
|  | Shaw *et al.* | Unranked | 2008 | [63] |
|  | Tafforeau *et al.* | Unranked | 2011 | [64] |
|  | Bradel-Tretheway *et al.* | Unranked | 2011 | [65] |
|  | Heaton *et al.* | Unranked | 2016 | [66] |
|  | Haas *et al.* | Unranked | 2023 | [56] |
|  | Wang *et al.* | Unranked | 2017 | [67] |
|  | Kuo *et al.* | Unranked | 2016 | [68] |
|  | Jorba *et al.* | Unranked | 2008 | [69] |
|  | Lin *et al.* | Unranked | 2012 | [70] |
| COIP | Watanabe *et al.* | Unranked | 2014 | [55] |
|  | Tripathi *et al.* | Unranked | 2015 | [61] |
| APMS | Tripathi *et al.* | Unranked | 2015 | [61] |
| Proteomics | Dove *et al.* | RANKED | 2012 | [71] |
| SingleCell | Sun *et al.* | RANKED | 2020 | [72] |

**Table S5**. Sars-Cov-2 data sources used to construct the MAIC gold standard

| **Category** | **Authors** | **Type** | **Year** | **Ref** |
| --- | --- | --- | --- | --- |
| vRNA–host protein | Schmidt *et al.* | RANKED | 2021 | [73] |
|  | Zhang *et al.* | RANKED | 2022 | [74] |
| CRISPR | Baggen *et al.* | RANKED | 2021 | [75] |
|  | Zhu *et al.* | RANKED | 2021 | [76] |
|  | Wei *et al.* | RANKED | 2021 | [77] |
|  | Wang *et al.* | RANKED | 2021 | [78] |
|  | Schneider *et al.* | RANKED | 2021 | [79] |
|  | Rebendenne *et al.* (3 screens) | RANKED | 2022 | [80] |
|  | Israeli *et al.* | RANKED | 2022 | [81] |
|  | Grozki *et al.* (2 screens) | RANKED | 2022 | [82] |
|  | Daniloski *et al.* | RANKED | 2021 | [83] |
|  | Biering *et al.* | RANKED | 2022 | [84] |
|  | Hou *et al.* | RANKED | 2024 | [85] |
|  | Ugalde *et al.* | RANKED | 2022 | [86] |
|  | Yousefi *et al.* | RANKED | 2023 | [87] |
|  | Hoffmann *et al.* (33C, 37C ) | RANKED | 2021 | [88] |
|  | Pagis *et al.* | Unranked | 2023 | [89] |
|  | Frasson *et al.* | Unranked | 2024 | [90] |
|  | Sakai *et al.* | RANKED | 2024 | [91] |
|  | Rehfeld *et al.* | RANKED | 2023 | [92] |
| Secondary screen | Rebendenne *et al.* (4 screens) | RANKED | 2022 | [80] |
| Protein interaction | Li *et al.* | UNRANKED | 2021 | [93] |
|  | Kim *et al.* | UNRANKED | 2023 | [94] |
|  | Bamberger *et al.* | UNRANKED | 2023 | [95] |
|  | Pahmeier *et al.* | UNRANKED | 2023 | [96] |
|  | Zhou *et al.* | UNRANKED | 2023 | [97] |
|  | Yang *et al.* | UNRANKED | 2024 | [98] |
|  | Chen *et al.* | UNRANKED | 2021 | [99] |
|  | Nabeel-Shah *et al.* | UNRANKED | 2022 | [100] |
|  | Gordon *et al.* | UNRANKED | 2020 | [101] |
|  | Stukalov *et al.* | UNRANKED | 2021 | [102] |
| SingleCell | Ravindra *et al.* | UNRANKED | 2021 | [103] |
|  | Wyler *et al.* | UNRANKED | 2021 | [104] |

**Table S6**. EBOV data sources used to construct the MAIC gold standard

| **Category** | **Authors** | **Type** | **Year** | **Ref** |
| --- | --- | --- | --- | --- |
| vRNA–host protein | Fang *et al.* | UNRANKED | 2018 | [105] |
|  | Zhang *et al.* | RANKED | 2022 | [74] |
| CRISPR | Gong *et al.* | RANKED | 2024 | [106] |
|  | Flint *et al.* | RANKED | 2019 | [107] |
|  | Cheng *et al.* | RANKED | 2025 | [24] |
|  | Carlson *et al.* | RANKED | 2025 | [108] |
| Haploid screen | Carette *et al.* | RANKED | 2011 | [109] |
| RNAi | Martin *et al.* | RANKED | 2018 | [110] |
|  | Yu *et al.* | UNRANKED | 2018 | [111] |
| Protein interaction | Batra *et al.* | UNRANKED | 2018 | [112] |
|  | Chen *et al.* | UNRANKED | 2019 | [113] |
|  | Morwitzer *et al.* | UNRANKED | 2019 | [114] |
|  | García-Dorival *et al.* | UNRANKED | 2014 | [115] |

**Table S7.** Performance scores of the different models

| Virus | Model | Balanced Acc. | F1 | Precision | AUROC |
| --- | --- | --- | --- | --- | --- |
| SARS‑CoV‑2 | Full HAN | 0.82± 0.005 | 0.77± 0.004 | 0.86± 0.001 | 0.915 ± 0.001 |
|  | RF | 0.85± 0.008 | 0.81± 0.005 | 0.85± 0.009 | 0.92 ± 0.012 |
|  | XGBoost | 0.78± 0.015 | 0.78± 0.020 | 0.79± 0.006 | 0.86 ± 0.006 |
|  | RF+ HAN Ensemble | 0.89± 0.002 | 0.85± 0.005 | 0.83± 0.009 | 0.95 ± 0.003 |
| IAV | Full HAN | 0.84± 0.002 | 0.78± 0.003 | 0.70± 0.004 | 0.90± 0.001 |
|  | RF | 0.80± 0.008 | 0.73± 0.001 | 0.78± 0.002 | 0.88± 0.001 |
|  | XGBoost | 0.78± 0.013 | 0.73± 0.008 | 0.68± 0.002 | 0.79± 0.003 |
|  | RF+ HAN Ensemble | 0.85± 0.007 | 0.81± 0.002 | 0.89± 0.005 | 0.93± 0.006 |
| ZIKV | Full HAN | 0.86± 0.005 | 0.89± 0.008 | 0.85± 0.004 | 0.89± 0.012 |
|  | RF | 0.91± 0.001 | 0.93± 0.00 | 0.92± 0.007 | 0.97± 0.005 |
|  | XGBoost | 0.90± 0.005 | 0.91± 0.006 | 0.87± 0.002 | 0.93± 0.002 |
|  | RF+ HAN Ensemble | 0.88± 0.003 | 0.89± 0.007 | 0.83± 0.003 | 0.93± 0.005 |
| DENV | Full HAN | 0.86± 0.001 | 0.89± 0.003 | 0.81± 0.002 | 0.84± 0.005 |
|  | RF | 0.92± 0.002 | 0.91± 0.005 | 0.89± 0.006 | 0.96± 0.013 |
|  | XGBoost | 0.89± 0.003 | 0.89± 0.013 | 0.88± 0.005 | 0.94± 0.030 |
|  | RF+ HAN Ensemble | 0.88± 0.002 | 0.89± 0.005 | 0.92± 0.001 | 0.90± 0.001 |
| EBOV | Full HAN | 0.72± 0.003 | 0.34± 0.009 | 0.61± 0.006 | 0.70± 0.002 |
|  | RF | 0.64± 0.009 | 0.67± 0.013 | 0.72± 0.008 | 0.72± 0.003 |
|  | XGBoost | 0.65± 0.021 | 0.70± 0.005 | 0.74± 0.003 | 0.68± 0.016 |
|  | RF+ HAN Ensemble | 0.69± 0.005 | 0.71± 0.003 | 0.75± 0.000 | 0.76± 0.009 |

**Table S8.** Performances from ablation experiments

| **Virus** | **Model variant** | **Description** | **AUROC (±SD)** | **AUPRC (±SD)** |
| --- | --- | --- | --- | --- |
| SARS‑CoV‑2 | Full HAN | virus token + full feature set + All categories | **0.915 ± 0.001** | **0.892 ± 0.015** |
|  | −virus token | Remove virus token | 0.837 ± 0.012 | 0.798 ± 0.008 |
|  | −Topology/Embeddings features | Exclude PPI topology, node2vec and localization | 0.87 ± 0.005 | 0.851 ± 0.009 |
|  | −GO/Pathway enrichment features | Remove GO and pathway enrichment features | 0.851 ± 0.005 | 0.834 ± 0.003 |
| IAV | Full HAN | virus token + full feature set + All categories | **0.902 ± 0.001** | **0.875 ± 0.002** |
|  | −virus token | Remove virus token | 0.831 ± 0.001 | 0.822 ± 0.003 |
|  | −Topology/Embeddings features | Exclude PPI topology, node2vec and localization | 0.856 ± 0.005 | 0.857 ± 0.001 |
|  | −GO/Pathway enrichment features | Remove GO and pathway enrichment features | 0.842 ± 0.008 | 0.846 ± 0.012 |
| ZIKV | Full HAN | virus token + full feature set + All categories | **0.897 ± 0.012** | **0.878 ± 0.007** |
|  | −virus token | Remove virus token | 0.864 ± 0.009 | 0.822 ± 0.005 |
|  | −Topology/Embeddings features | Exclude PPI topology, node2vec and localization | 0.854 ± 0.003 | 0.841 ± 0.008 |
|  | −GO/Pathway enrichment features | Remove GO and pathway enrichment features | 0.795 ± 0.009 | 0.802 ± 0.001 |
| DENV | Full HAN | virus token + full feature set + All categories | **0.841 ± 0.005** | **0.833 ± 0.009** |
|  | −virus token | Remove virus token | 0.825 ± 0.009 | 0.792 ± 0.016 |
|  | −Topology/Embeddings features | Exclude PPI topology, node2vec and localization | 0.782 ± 0.014 | 0.763 ± 0.008 |
|  | −GO/Pathway enrichment features | Remove GO and pathway enrichment features | 0.805 ± 0.008 | 0.772 ± 0.010 |
| EBOV | Full HAN | virus token + full feature set + All categories | **0.704 ± 0.002** | **0.624 ± 0.006** |
|  | −virus token | Remove virus token | 0.662 ± 0.013 | 0.60 ± 0.017 |
|  | −Topology/Embeddings features | Exclude PPI topology, node2vec and localization | 0.644 ± 0.012 | 0.621 ± 0.025 |
|  | −GO/Pathway enrichment features | Remove GO and pathway enrichment features | 0.651 ± 0.008 | 0.624 ± 0.009 |

**Table S9**. Pairwise overlaps and p-values for top-1000 virus-specific gene sets at the Experimental, MAIC, and ML levels

| **level** | **virus1** | **virus2** | overlap | **P-value** |
| --- | --- | --- | --- | --- |
| Experimental | SARSCoV2 | IAV | 124 | 1.20E-20 |
|  |  | ZIKV | 103 | 5.39E-12 |
|  |  | DENV | 161 | 1.47E-40 |
|  | IAV | ZIKV | 93 | 1.21E-08 |
|  |  | DENV | 187 | 1.15E-57 |
|  | ZIKV | DENV | 139 | 4.77E-28 |
| MAIC | SARSCoV2 | IAV | 179 | 6.46E-83 |
|  |  | ZIKV | 159 | 6.46E-83 |
|  |  | DENV | 201 | 4.62E-106 |
|  | IAV | ZIKV | 301 | 8.63E-241 |
|  |  | DENV | 313 | 0.00E+00 |
|  | ZIKV | DENV | 385 | 8.63E-80 |
| ML | SARSCoV2 | IAV | 321 | 4.31E-271 |
|  |  | ZIKV | 383 | 4.56E-286 |
|  |  | DENV | 436 | 0 |
|  | IAV | ZIKV | 365 | 0 |
|  |  | DENV | 428 | 2.30E-295 |
|  | ZIKV | DENV | 478 | 0 |

**Tables S10-S12** are provided in a separate spreadsheet.

**Table S13**. Pan-viral HDF signature is enriched among MAIC-prioritized EBOV candidates^*^

| **Comparison** | **Observed overlap** | **Expected overlap** | **Fold-enrichment** | **Approx. OR** | **P-value** |
| --- | --- | --- | --- | --- | --- |
| 427 vs EBOV Top-100 | 23 | 2.4 | 9.7× | 12.9 | 1.1e-16 |
| 427 vs EBOV Top-200 | 44 | 4.7 | 9.3× | 12.8 | 5.0e-30 |
| 427 vs EBOV Top-500 | 84 | 11.9 | 7.1× | 10.1 | 1.9e-47 |
| 427 vs EBOV Top-1000 | 128 | 23.7 | 5.4× | 8.2 | 1.6e-59 |
| 118 vs EBOV Top-1000 | 41 | 6.6 | 6.3× | 9.4 | 2.4e-22 |

^*^Enrichment of the ≥3-virus pan-viral set (n = 427) and the 4/4 core set (n = 118) among the top EBOV MAIC-ranked genes. For different thresholds (top 100, 200, 500, 1000), the table reports the observed overlap with the EBOV list, the expected overlap under a hypergeometric model (background = all genes scored by MAIC for EBOV), the fold-enrichment (observed/expected), an approximate odds ratio (OR) from the 2×2 contingency table, and p-values derived from a right-tailed hypergeometric enrichment test. Enrichment is strong and consistent across all thresholds, supporting cross-family generalization of the pan-viral host signature to filoviruses not used in model training.

**Table S14.** Performances for optimal parameters of HAN*

| **Virus** | **HIDDEN** | **HEADS** | **DROPOUT** | **WD** | **K_VH (V→H)** | **K_VV (V→V)** | **K_HH φ2/φ3** | **Post-MHA** | **AUROC (mean±SD)** | **AUPRC (mean±SD)** |
| --- | --- | --- | --- | --- | --- | --- | --- | --- | --- | --- |
| SARS-CoV-2 | 256 | 4 | 0.3 | 1e-5 | 25 | 15 | 10 / 5 | ON | 0.923 ± 0.009 | 0.897 ± 0.011 |
| IAV | 256 | 4 | 0.3 | 1e-4 | 25 | 10 | 10 / 5 | ON | 0.911 ± 0.012 | 0.884 ± 0.016 |
| ZIKV | 128 | 4 | 0.3 | 1e-5 | 15 | 10 | 10 / 5 | ON | 0.899 ± 0.006 | 0.888 ± 0.013 |
| DENV | 128 | 2 | 0.2 | 1e-5 | 15 | 10 | 10 / 5 | ON | 0.856 ± 0.010 | 0.841 ± 0.012 |
| EBOV | 128 | 2 | 0.3 | 1e-4 | 10 | 5 | 10 / 5 | OFF | 0.716 ± 0.023 | 0.625 ± 0.019 |

^*^This table summarizes the results from optimal parameters combinations. The search space was as follows:

Model capacity: HIDDEN ∈ {128, 256, 512}, HEADS ∈ {2, 4, 8}, DROPOUT ∈ {0.2, 0.3, 0.5}; Optimizer: Adam with LR ∈ {5e-4, 1e-3, 2e-3}, weight decay (WD) ∈ {1e-5, 1e-4, 5e-4}; Meta-path budgets: K_VH ∈ {10, 15, 25, 40} (V→H), K_VV ∈ {5, 10, 20} (V→V), K_HH (φ2/φ3) ∈ {(5/3), (10/5), (20/10)} for the two H→H hops used in meta-path composition Fusion / virus token: Post-MHA ∈ {ON, OFF}.

# **Supplementary Text**

**Supplementary Text S1. Ablation studies**

#

To assess which data sources were most important for generalization, we performed two sets of ablation experiments: (i) removal of individual evidence categories used to build the MAIC gold-standard labels, and (ii) removal of major feature blocks in the HAN model.

First, we asked how each experimental evidence type contributed to model performance by rebuilding the MAIC scores while leaving out one category at a time and retraining the models (**Fig.** **S2**). Across all viruses and metrics (AUROC, balanced accuracy, F1, precision, recall), the All-categories setting yielded the best performance, indicating that the different evidence sources provide complementary rather than redundant information. The pattern was virus-specific: for SARS-CoV-2, removing protein interaction evidence (virus–host PPI) caused the strongest degradation, whereas for IAV, ZIKV, DENV, and EBOV, leaving out CRISPR knockout screens led to the largest drop, with RNAi, scRNA-seq, and RNA-centric data contributing smaller but still consistent performance gains. These trends were similar for both the HAN model and the RF+HAN ensemble and are in line with the view that combining heterogeneous experimental modalities is necessary to obtain robust benchmarks.

Second, we examined which major feature groups inside the HAN model were most critical (**Table S9**). For this analysis, we focused on three conceptually distinct blocks: (i) the virus token, which encodes which virus is being modelled; (ii) network topology and embeddings (PPI-based centrality, node2vec embeddings, and localization features); and (iii) GO / pathway enrichment features derived from gene-set analysis. Across viruses, the full HAN with all three components active was consistently among the best-performing models and therefore served as the reference. Removing the virus token produced the largest performance drops for SARS-CoV-2 and IAV, highlighting the value of making the viral context explicit in the graph. For ZIKV, ablating GO / pathway enrichment led to the strongest decrease in AUROC/AUPRC, whereas for DENV and EBOV, removing network topology and embedding features had the largest impact. We restricted feature ablations to these three blocks because preliminary experiments showed that they had the largest effect on performance, and they correspond directly to the main biological information channels in our framework (viral identity, network context, and functional context). Together, these ablation studies demonstrate that each information channel adds non-redundant signal, that the most informative sources differ between viruses, and that integrating all available evidence and feature blocks is necessary to reach the best overall performance.

**Supplementary Text S2. Network organization and gene-family composition of prioritized pan-viral HDF**

We prioritized predicted HDF using a multi-criteria scheme designed to balance robustness and biological interpretability. First, core-essential genes were removed using the OGEE database (606 genes were denoted to be essential, as they were listed to be essential in ≥ 80 % of different cell lines) [116]. Next, we restricted attention to genes that are expressed in the relevant cellular context. To this end, we re-analysed bulk RNA-seq data from A549 cells infected with IAV and SARS-CoV-2 (GSE147507), DENV (GSE84285) and ZIKV (GSE265922), using only virus-infected replicates and keeping time point/MOI choices consistent within each virus dataset. Raw counts were converted to TPM per replicate, for each dataset we computed the median TPM per gene across replicates and mapped this to a 0–100 percentile across all protein-coding genes. A gene passed the expression filter if it reached at least the 20th percentile in ≥3 out of 4 expression datasets and at least the 50th percentile in at least one dataset. Each candidate gene then received a score from the predictions of the four previously trained machine learning model across the four viruses. For each gene, we counted in how many viruses it was predicted to be an HDF and assigned a “prior consensus” score from 1 (only one virus) to 4 (all four viruses: SARS-CoV-2, IAV, DENV, ZIKV). Next, a human molecular network was constructed based on the STRING database v12 (highest confidence 0.9, physical/experimental evidence), and degree, closeness and betweenness centrality were combined into a single rank product value. Only genes with a rank product greater than or equal to the median were retained. PubMed searches were carried out per gene–virus pair using the gene symbol and the keyword “host factor”. The resulting citation counts were scaled and used as an additional feature. In addition to these virus-specific scores, we derived a pan-viral prioritization across all four viruses. For each gene, we z-scored the continuous features (network-centrality index, summed expression percentiles across viruses and total PubMed citations) and combined them into a composite score, while including the prior-consensus score. Genes were then globally ordered by this composite score, which prioritizes candidates that are (i) well-expressed in the infected A549 context, (ii) centrally positioned in the host interaction network, (iii) supported by multiple virus-specific models and (iv) at least partially documented in the literature. Following the completion of these steps, a total of 210 prioritized genes were identified, the protein-protein interaction network of which is depicted in **Fig. S3**. To summarize the compositional landscape of the prioritized genes, we mapped each gene to curated HGNC gene families (with PANTHER as a fallback) and visualized their relative abundance (**Fig. S4**).

Analysis of the protein–protein interaction network revealed dense connectivity around core host processes (**Fig. S3**). For example, translation initiation factors (EIF4A1, PABPC1) and ribosomal proteins co-occur with hnRNP family members among the top-ranked genes, reflecting viral dependence on host protein synthesis. DEAD-box RNA helicases (e.g., DDX3X, DDX46) likewise emerged as prominent HDF, consistent with their roles in viral RNA synthesis. Nuclear export factors (XPO1, IPO7) and endosomal GTPases (RAB5, RAB7A) are enriched among the prioritized genes, consistent with viral entry and egress pathways. For example, EIF4A1 is required for SARS-CoV-2 protein synthesis, and clathrin heavy chain CLTC, a mediator of endocytosis, is essential for SARS-CoV-2 entry. Similarly, IAV depends on vacuolar-ATPase and mRNA export factors (ATP6AP1, NXF1), top hits in genome-wide IAV screens. These findings reflect a pan-viral pattern: translation, endocytic, and nuclear export factors are repeatedly co-opted by diverse viruses. Proteostasis factors are also prominent: UBE2N, WWP1, and proteasome subunits score highly, consistent with viral subversion of the ubiquitin–proteasome system. Gene-family mapping confirmed that hnRNPs, DEAD-box helicases, ribosomal and proteasome subunits, and RING-finger ubiquitin ligases dominate the set. Together, these observations delineate a network of conserved pan-viral host dependency factors centered on translation, RNA metabolism, intracellular trafficking, and proteostasis (**Fig. S4**).

**Supplementary Text S3. Exploratory extension of the framework to Epstein–Barr virus (EBV)**

An important question is whether the present machine-learning framework can be extended to human oncogenic viruses. In principle, this appears plausible, but such an extension would require a virus-specific and stage-aware definition of host dependency, because oncogenic viruses often rely on persistence-associated processes such as latency, reactivation, episomal maintenance, cccDNA regulation, viral integration, or tumor-supportive host programs rather than on a single acute replication state. As an exploratory test, we selected Epstein–Barr virus (EBV) because, among the oncogenic viruses evaluated, it provided the richest set of usable high-throughput data and therefore represented the most informative first candidate for extending the framework. The final EBV benchmark assembled here comprised six evidence lists: four genetic lists derived from two CRISPR-based studies interrogating host control of lytic reactivation and dependencies of EBV-transformed B cells, together with two virus–host protein interaction datasets [117], [118], [119], [120]. Even in this comparatively favorable setting, the data showed biologically and technically rather high heterogeneity, as the retained datasets sampled different viral states, cellular contexts, and assay readouts, and therefore captured rather divers than complementary dependency signals. Notably, the overlap across the datasets was very low. Our analysis identified no common gene of three or more of the six EBV evidence lists. An overlap in two datasets was also very limited (4.1%) (Figure S7). In addition, no transcriptome or RNA-centric layer met our inclusion criteria for the final EBV benchmark, leaving the evidence structure sparse and modality-imbalanced. Consistent with this structure, predictive performance in this preliminary EBV analysis remained modest (AUC–ROC ~0.63), even though significantly better than random guessing. We interpret this result not as evidence against the general applicability of the framework, but rather as an indication that dataset availability alone is insufficient when biologically distinct dependency states are collapsed into a single benchmark. In EBV, the integrated datasets do not reflect one unified host-dependency phenotype, but instead combine distinct processes including lytic reactivation control, latent or transformed-cell fitness, and physical virus–host association. Compared with the acute RNA viruses analyzed in the main study, this leads to a less harmonized benchmarks for machine learning. Overall, these observations suggest that extension of HDF-based multi-omics integration to oncogenic viruses is promising, but will likely require more refinement on the construction of the benchmarking process.

**Supplementary Text S4. Search terms**

For the literature search, we applied the following search pattern:

(("SARS-CoV-2"[Mesh] OR "SARS-CoV-2"[tiab] OR COVID-19[tiab]) OR ("Zika Virus"[Mesh] OR Zika[tiab] OR ZIKV[tiab]) OR ("Dengue Virus"[Mesh] OR Dengue[tiab] OR DENV[tiab]) OR ("Ebolavirus"[Mesh] OR Ebola[tiab] OR EBOV[tiab]) OR ("Influenza A virus"[Mesh] OR "Influenza A"[tiab] OR IAV[tiab] OR H1N1[tiab] OR H3N2[tiab] OR H5N1[tiab]))

AND ( CRISPR[tiab] OR "CRISPR-Cas9"[tiab] OR RNAi[tiab] OR siRNA[tiab] OR shRNA[tiab] OR "gene trap"[tiab] OR haploid[tiab] OR "genome-wide"[tiab] OR "pooled screen"[tiab] OR dropout[tiab]

OR "affinity purification"[tiab] OR "AP-MS"[tiab] OR "IP-MS"[tiab] OR "TAP-MS"[tiab]

OR immunoprecipitation[tiab] OR "co-IP"[tiab] OR BioID[tiab] OR TurboID[tiab] OR APEX[tiab]

OR "yeast two-hybrid"[tiab] OR Y2H[tiab] OR HuProt[tiab] OR "protein microarray"[tiab]

OR "ChIRP-MS"[tiab] OR "RAP-MS"[tiab] OR RaPID[tiab] OR "TUX-MS"[tiab] OR OOPS[tiab]

OR CLIP[tiab] OR iCLIP[tiab] OR eCLIP[tiab] OR irCLIP[tiab] OR SPLASH[tiab] OR COMRADES[tiab]

OR "RNA interactome"[tiab] OR "RNA-seq"[tiab] OR transcriptom*[tiab] OR "single-cell"[tiab] OR scRNA-seq[tiab] OR "MeRIP-seq"[tiab] OR "m6A-seq"[tiab] OR proteomic*[tiab] OR phosphoproteomic*[tiab] OR ubiquitinom*[tiab] OR "SOMAscan"[tiab] OR TMT[tiab] OR SILAC[tiab] OR "mass spectrometry"[tiab])

AND ("host dependency factor"[tiab] OR "host dependency"[tiab] OR "host-dependency"[tiab] OR proviral[tiab] OR "pro-viral"[tiab] OR "host factor"[tiab] OR "host factors"[tiab] OR HDF[tiab])

AND ("2000/01/01"[dp] : "3000/12/31"[dp])

(("SARS-CoV-2" OR "COVID-19" OR "COVID 19" OR "2019-nCoV") OR ("Zika virus" OR Zika OR ZIKV OR ZIKV*) OR ("Dengue virus" OR Dengue OR DENV OR DENV*) OR (Ebolavirus OR Ebola OR EBOV OR "Zaire ebolavirus") OR ("Influenza A" OR "Influenza A virus" OR IAV OR H1N1 OR H3N2 OR H5N1))

AND (CRISPR OR "CRISPR-Cas9" OR RNAi OR siRNA OR shRNA OR "gene trap" OR haploid OR "genome-wide" OR "pooled screen" OR dropout OR "AP-MS" OR APMS OR "IP-MS" OR "TAP-MS" OR immunoprecipitat* OR "co-IP" OR coimmunoprecipitat* OR BioID OR "bioid" OR TurboID OR APEX OR APEX2 OR "yeast two-hybrid" OR Y2H OR HuProt OR "protein microarray" OR "ChIRP-MS" OR "RAP-MS" OR RaPID OR "TUX-MS" OR OOPS OR CLIP OR iCLIP OR eCLIP OR irCLIP OR SPLASH OR COMRADES OR "RNA interactome" OR "RNA-seq" OR transcriptom* OR "single-cell" OR "single cell" OR scRNA-seq OR "MeRIP-seq" OR "m6A-seq" OR proteomic* OR phosphoproteomic* OR ubiquitinom* OR "SOMAscan" OR TMT OR SILAC OR "mass spectrometry")

AND ("host dependency factor" OR "host dependency" OR "host-dependency" OR proviral OR "pro-viral" OR "host factor" OR "host factors" OR HDF)

**References**

[1] S. Zhang *et al.*, “Comparison of viral RNA–host protein interactomes across pathogenic RNA viruses informs rapid antiviral drug discovery for SARS-CoV-2,” *Cell Res.*, vol. 32, no. 1, pp. 9–23, 2022, doi: 10.1038/s41422-021-00581-y.

[2] Y. S. Ooi *et al.*, “An RNA-centric dissection of host complexes controlling flavivirus infection,” *Nat. Microbiol.*, vol. 4, no. 12, pp. 2369–2382, 2019, doi: 10.1038/s41564-019-0518-2.

[3] K. C. Liao *et al.*, “Dengue and Zika RNA-RNA interactomes reveal pro- and anti-viral RNA in human cells,” *Genome Biol.*, vol. 24, no. 1, pp. 1–17, 2023, doi: 10.1186/s13059-023-03110-9.

[4] B. Shue *et al.*, “Genome-Wide CRISPR Screen Identifies RACK1 as a Critical Host Factor for Flavivirus Replication,” *J. Virol.*, vol. 95, no. 24, Nov. 2021, doi: 10.1128/JVI.00596-21,.

[5] Y. Li *et al.*, “Genome-wide CRISPR screen for Zika virus resistance in human neural cells,” *Proc. Natl. Acad. Sci. U. S. A.*, vol. 116, no. 19, pp. 9527–9532, May 2019, doi: 10.1073/PNAS.1900867116,.

[6] G. Savidis *et al.*, “Identification of Zika Virus and Dengue Virus Dependency Factors using Functional Genomics,” *Cell Rep.*, vol. 16, no. 1, pp. 232–246, Jun. 2016, doi: 10.1016/J.CELREP.2016.06.028,.

[7] S. Wang *et al.*, “Integrin αvβ5 Internalizes Zika Virus during Neural Stem Cells Infection and Provides a Promising Target for Antiviral Therapy,” *Cell Rep.*, vol. 30, no. 4, pp. 969-983.e4, Jan. 2020, doi: 10.1016/j.celrep.2019.11.020.

[8] M. Rother and M. Naumann, “Signal peptidase complex subunit 1 is an essential Zika virus host factor in placental trophoblasts,” *Virus Res.*, vol. 296, Apr. 2021, doi: 10.1016/J.VIRUSRES.2021.198338,.

[9] G. Song *et al.*, “An Integrated Systems Biology Approach Identifies the Proteasome as A Critical Host Machinery for ZIKV and DENV Replication,” *Genomics Proteomics Bioinformatics*, vol. 19, no. 1, pp. 108–122, Feb. 2021, doi: 10.1016/J.GPB.2020.06.016,.

[10] P. S. Shah *et al.*, “Comparative Flavivirus-Host Protein Interaction Mapping Reveals Mechanisms of Dengue and Zika Virus Pathogenesis,” *Cell*, vol. 175, no. 7, pp. 1931-1945.e18, Dec. 2018, doi: 10.1016/j.cell.2018.11.028.

[11] P. Scaturro *et al.*, “An orthogonal proteomic survey uncovers novel Zika virus host factors,” *Nature*, vol. 561, no. 7722, pp. 253–257, Sep. 2018, doi: 10.1038/S41586-018-0484-5,.

[12] B. D. Persson *et al.*, “BAF45b is required for efficient zika virus infection of HAP1 cells,” *Viruses*, vol. 13, no. 10, Oct. 2021, doi: 10.3390/V13102007,.

[13] E. Coyaud *et al.*, “Global interactomics uncovers extensive organellar targeting by Zika Virus,” *Molecular and Cellular Proteomics*, vol. 17, no. 11, pp. 2242–2255, Nov. 2018, doi: 10.1074/mcp.TIR118.000800.

[14] D. Kovanich *et al.*, “Analysis of the Zika and Japanese Encephalitis Virus NS5 Interactomes,” *J. Proteome Res.*, vol. 18, no. 8, pp. 3203–3218, Aug. 2019, doi: 10.1021/ACS.JPROTEOME.9B00318,.

[15] V. A. Golubeva *et al.*, “Network of interactions between ZIKA virus non structural proteins and human host proteins,” *Cells*, vol. 9, no. 1, Jan. 2020, doi: 10.3390/CELLS9010153,.

[16] J. Zeng *et al.*, “The Zika Virus Capsid Disrupts Corticogenesis by Suppressing Dicer Activity and miRNA Biogenesis,” *Cell Stem Cell*, vol. 27, no. 4, pp. 618-632.e9, Oct. 2020, doi: 10.1016/j.stem.2020.07.012.

[17] F. Zanini, S. Y. Pu, E. Bekerman, S. Einav, and S. R. Quake, “Single-cell transcriptional dynamics of flavivirus infection,” *Elife*, vol. 7, Feb. 2018, doi: 10.7554/ELIFE.32942.

[18] O. V. Viktorovskaya, T. M. Greco, I. M. Cristea, and S. R. Thompson, “Identification of RNA Binding Proteins Associated with Dengue Virus RNA in Infected Cells Reveals Temporally Distinct Host Factor Requirements,” *PLoS Negl. Trop. Dis.*, vol. 10, no. 8, Aug. 2016, doi: 10.1371/JOURNAL.PNTD.0004921.

[19] S. L. Phillips, E. J. Soderblom, S. S. Bradrick, and M. A. Garcia-Blanco, “Identification of Proteins Bound to Dengue Viral RNA In Vivo Reveals New Host Proteins Important for Virus Replication,” *mBio*, vol. 7, no. 1, Jan. 2016, doi: 10.1128/MBIO.01865-15.

[20] C. D. Marceau *et al.*, “Genetic dissection of Flaviviridae host factors through genome-scale CRISPR screens,” *Nature*, vol. 535, no. 7610, pp. 159–163, Jul. 2016, doi: 10.1038/NATURE18631.

[21] D. L. Lin, N. A. Cherepanova, L. Bozzacco, M. R. Macdonald, R. Gilmore, and A. W. Tai, “Dengue Virus Hijacks a Noncanonical Oxidoreductase Function of a Cellular Oligosaccharyltransferase Complex,” *mBio*, vol. 8, no. 4, Jul. 2017, doi: 10.1128/MBIO.00939-17.

[22] L. Belmont *et al.*, “Functional genomics screens reveal a role for TBC1D24 and SV2B in antibody-dependent enhancement of dengue virus infection,” *bioRxiv*, Apr. 2024, doi: 10.1101/2024.04.26.591029.

[23] S. Shivaprasad, W. Qiao, K. F. Weng, P. Umashankar, J. E. Carette, and P. Sarnow, “CRISPR Screen Reveals PACT as a Pro-Viral Factor for Dengue Viral Replication,” *Viruses*, vol. 16, no. 5, May 2024, doi: 10.3390/V16050725.

[24] K. W. Cheng *et al.*, “Genome-wide CRISPR knockout screening with viral replicons for identification of host factors involved in viral replication,” *bioRxiv*, p. 2025.01.09.632058, Jan. 2025, doi: 10.1101/2025.01.09.632058.

[25] A. Labeau *et al.*, “A Genome-Wide CRISPR-Cas9 Screen Identifies the Dolichol-Phosphate Mannose Synthase Complex as a Host Dependency Factor for Dengue Virus Infection,” *J. Virol.*, vol. 94, no. 7, Mar. 2020, doi: 10.1128/JVI.01751-19.

[26] O. M. Sessions *et al.*, “Discovery of insect and human dengue virus host factors,” *Nature*, vol. 458, no. 7241, pp. 1047–1050, Apr. 2009, doi: 10.1038/NATURE07967.

[27] N. J. Barrows *et al.*, “Dual roles for the ER membrane protein complex in flavivirus infection: viral entry and protein biogenesis,” *Sci. Rep.*, vol. 9, no. 1, Dec. 2019, doi: 10.1038/S41598-019-45910-9.

[28] M. N. Krishnan *et al.*, “RNA interference screen for human genes associated with West Nile virus infection,” *Nature*, vol. 455, no. 7210, pp. 242–245, Sep. 2008, doi: 10.1038/NATURE07207.

[29] Y. J. Kwon *et al.*, “Kinome siRNA screen identifies novel cell-type specific dengue host target genes,” *Antiviral Res.*, vol. 110, no. 1, pp. 20–30, 2014, doi: 10.1016/j.antiviral.2014.07.006.

[30] M. Cortese *et al.*, “Reciprocal Effects of Fibroblast Growth Factor Receptor Signaling on Dengue Virus Replication and Virion Production,” *Cell Rep.*, vol. 27, no. 9, pp. 2579-2592.e6, May 2019, doi: 10.1016/J.CELREP.2019.04.105.

[31] S. Khadka *et al.*, “A physical interaction network of dengue virus and human proteins,” *Molecular and Cellular Proteomics*, vol. 10, no. 12, Dec. 2011, doi: 10.1074/mcp.M111.012187.

[32] M. L. Hafirassou *et al.*, “A Global Interactome Map of the Dengue Virus NS1 Identifies Virus Restriction and Dependency Host Factors,” *Cell Rep.*, vol. 21, no. 13, pp. 3900–3913, Dec. 2017, doi: 10.1016/j.celrep.2017.11.094.

[33] F. A. De Maio *et al.*, “The Dengue Virus NS5 Protein Intrudes in the Cellular Spliceosome and Modulates Splicing,” *PLoS Pathog.*, vol. 12, no. 8, Aug. 2016, doi: 10.1371/JOURNAL.PPAT.1005841.

[34] D. Mairiang *et al.*, “Identification of new protein interactions between dengue fever virus and its hosts, human and mosquito,” *PLoS One*, vol. 8, no. 1, Jan. 2013, doi: 10.1371/JOURNAL.PONE.0053535.

[35] E. M. Silva, J. N. Conde, D. Allonso, M. L. Nogueira, and R. Mohana-Borges, “Mapping the interactions of dengue virus NS1 protein with human liver proteins using a yeast two-hybrid system: identification of C1q as an interacting partner,” *PLoS One*, vol. 8, no. 3, Mar. 2013, doi: 10.1371/JOURNAL.PONE.0057514.

[36] M. Cervantes-Salazar *et al.*, “Dengue virus NS1 protein interacts with the ribosomal protein RPL18: This interaction is required for viral translation and replication in Huh-7 cells,” *Virology*, vol. 484, pp. 113–126, Oct. 2015, doi: 10.1016/j.virol.2015.05.017.

[37] T. Dechtawewat *et al.*, “Mass spectrometric analysis of host cell proteins interacting with dengue virus nonstructural protein 1 in dengue virus-infected HepG2 cells,” *Biochim. Biophys. Acta Proteins Proteom.*, vol. 1864, no. 9, pp. 1270–1280, Sep. 2016, doi: 10.1016/j.bbapap.2016.04.008.

[38] E. M. Silva *et al.*, “Dengue virus nonstructural 3 protein interacts directly with human glyceraldehyde-3-phosphate dehydrogenase (GAPDH) and reduces its glycolytic activity,” *Sci. Rep.*, vol. 9, no. 1, Dec. 2019, doi: 10.1038/S41598-019-39157-7.

[39] L. N. Carpp, R. S. Rogers, R. L. Moritz, and J. D. Aitchison, “Quantitative proteomic analysis of host-virus interactions reveals a role for golgi brefeldin a resistance factor 1 (GBF1) in dengue infection,” *Molecular and Cellular Proteomics*, vol. 13, no. 11, pp. 2836–2854, Nov. 2014, doi: 10.1074/mcp.M114.038984.

[40] M. Le Breton *et al.*, “Flavivirus NS3 and NS5 proteins interaction network: a high-throughput yeast two-hybrid screen,” *BMC Microbiol.*, vol. 11, 2011, doi: 10.1186/1471-2180-11-234.

[41] T. Poyomtip *et al.*, “Development of viable TAP-tagged dengue virus for investigation of host-virus interactions in viral replication,” *J. Gen. Virol.*, vol. 97, no. 3, pp. 646–658, Mar. 2016, doi: 10.1099/JGV.0.000371.

[42] A. M. Ward *et al.*, “The Golgi associated ERI3 is a Flavivirus host factor,” *Sci. Rep.*, vol. 6, Sep. 2016, doi: 10.1038/SREP34379.

[43] K. Rabelo *et al.*, “The effect of the dengue non-structural 1 protein expression over the HepG2 cell proteins in a proteomic approach,” *J. Proteomics*, vol. 152, pp. 339–354, Jan. 2017, doi: 10.1016/j.jprot.2016.11.001.

[44] A. Brugier *et al.*, “RACK1 Associates with RNA-Binding Proteins Vigilin and SERBP1 to Facilitate Dengue Virus Replication,” *J. Virol.*, vol. 96, no. 7, Apr. 2022, doi: 10.1128/JVI.01962-21.

[45] S. Jansen *et al.*, “Identification of host factors binding to dengue and Zika virus subgenomic RNA by efficient yeast three-hybrid screens of the human ORFeome,” *RNA Biol.*, vol. 18, no. 5, pp. 732–744, 2021, doi: 10.1080/15476286.2020.1868754.

[46] J. Han *et al.*, “Genome-wide CRISPR/Cas9 Screen Identifies Host Factors Essential for Influenza Virus Replication,” *Cell Rep.*, vol. 23, no. 2, pp. 596–607, 2018, doi: 10.1016/j.celrep.2018.03.045.

[47] B. Li *et al.*, “Genome-wide CRISPR screen identifies host dependency factors for influenza A virus infection,” *Nat. Commun.*, vol. 11, no. 1, 2020, doi: 10.1038/s41467-019-13965-x.

[48] V. Tran *et al.*, “Influenza virus repurposes the antiviral protein IFIT2 to promote translation of viral mRNAs,” *Nat. Microbiol.*, vol. 5, no. 12, pp. 1490–1503, 2020, doi: 10.1038/s41564-020-0778-x.

[49] C. Yi *et al.*, “Genome-wide CRISPR-Cas9 screening identifies the CYTH2 host gene as a potential therapeutic target of influenza viral infection,” *Cell Rep.*, vol. 38, no. 13, p. 110559, 2022, doi: 10.1016/j.celrep.2022.110559.

[50] Y. Song *et al.*, “A genome-wide CRISPR/Cas9 gene knockout screen identifies immunoglobulin superfamily DCC subclass member 4 as a key host factor that promotes influenza virus endocytosis,” *PLoS Pathog.*, vol. 17, no. 12, Dec. 2021, doi: 10.1371/JOURNAL.PPAT.1010141.

[51] C. R. King *et al.*, “Pathogen-driven CRISPR screens identify TREX1 as a regulator of DNA self-sensing during influenza virus infection,” *Cell Host Microbe*, vol. 31, no. 9, pp. 1552-1567.e8, Sep. 2023, doi: 10.1016/j.chom.2023.08.001.

[52] W. C. Su *et al.*, “Pooled RNAi screen identifies ubiquitin ligase Itch as crucial for influenza A virus release from the endosome during virus entry,” *Proc. Natl. Acad. Sci. U. S. A.*, vol. 110, no. 43, pp. 17516–17521, 2013, doi: 10.1073/pnas.1312374110.

[53] A. T. Tran *et al.*, “Knockdown of specific host factors protects against influenza virus-induced cell death,” *Cell Death Dis.*, vol. 4, no. 8, Aug. 2013, doi: 10.1038/CDDIS.2013.296.

[54] S. D. Shapira *et al.*, “A Physical and Regulatory Map of Host-Influenza Interactions Reveals Pathways in H1N1 Infection,” *Cell*, vol. 139, no. 7, pp. 1255–1267, Dec. 2009, doi: 10.1016/j.cell.2009.12.018.

[55] T. Watanabe *et al.*, “Influenza virus-host interactome screen as a platform for antiviral drug development,” *Cell Host Microbe*, vol. 16, no. 6, pp. 795–805, 2014, doi: 10.1016/j.chom.2014.11.002.

[56] K. M. Haas *et al.*, “Proteomic and genetic analyses of influenza A viruses identify pan-viral host targets,” *Nat. Commun.*, vol. 14, no. 1, Dec. 2023, doi: 10.1038/S41467-023-41442-Z.

[57] A. L. Brass *et al.*, “The IFITM Proteins Mediate Cellular Resistance to Influenza A H1N1 Virus, West Nile Virus, and Dengue Virus,” *Cell*, vol. 139, no. 7, pp. 1243–1254, 2009, doi: 10.1016/j.cell.2009.12.017.

[58] A. Karlas *et al.*, “Genome-wide RNAi screen identifies human host factors crucial for influenza virus replication,” *Nature*, vol. 463, no. 7282, pp. 818–822, 2010, doi: 10.1038/nature08760.

[59] R. König *et al.*, “Human host factors required for influenza virus replication,” *Nature*, vol. 463, no. 7282, pp. 813–817, 2010, doi: 10.1038/nature08699.

[60] S. E. Ward *et al.*, “Host modulators of H1N1 cytopathogenicity,” *PLoS One*, vol. 7, no. 8, pp. 1–12, 2012, doi: 10.1371/journal.pone.0039284.

[61] S. Tripathi *et al.*, “Meta- and Orthogonal Integration of Influenza ‘oMICs’ Data Defines a Role for UBR4 in Virus Budding,” *Cell Host Microbe*, vol. 18, no. 6, pp. 723–735, Dec. 2015, doi: 10.1016/j.chom.2015.11.002.

[62] B. de Chassey *et al.*, “The interactomes of influenza virus NS1 and NS2 proteins identify new host factors and provide insights for ADAR1 playing a supportive role in virus replication,” *PLoS Pathog.*, vol. 9, no. 7, Jul. 2013, doi: 10.1371/JOURNAL.PPAT.1003440.

[63] M. L. Shaw, K. L. Stone, C. M. Colangelo, E. E. Gulcicek, and P. Palese, “Cellular proteins in influenza virus particles,” *PLoS Pathog.*, vol. 4, no. 6, Jun. 2008, doi: 10.1371/JOURNAL.PPAT.1000085.

[64] L. Tafforeau *et al.*, “Generation and comprehensive analysis of an influenza virus polymerase cellular interaction network,” *J. Virol.*, vol. 85, no. 24, pp. 13010–13018, Dec. 2011, doi: 10.1128/JVI.02651-10.

[65] B. G. Bradel-Tretheway *et al.*, “Comprehensive proteomic analysis of influenza virus polymerase complex reveals a novel association with mitochondrial proteins and RNA polymerase accessory factors,” *J. Virol.*, vol. 85, no. 17, pp. 8569–8581, Sep. 2011, doi: 10.1128/JVI.00496-11.

[66] N. S. Heaton *et al.*, “Targeting Viral Proteostasis Limits Influenza Virus, HIV, and Dengue Virus Infection,” *Immunity*, vol. 44, no. 1, pp. 46–58, Jan. 2016, doi: 10.1016/j.immuni.2015.12.017.

[67] L. Wang *et al.*, “Comparative influenza protein interactomes identify the role of plakophilin 2 in virus restriction,” *Nat. Commun.*, vol. 8, Feb. 2017, doi: 10.1038/NCOMMS13876.

[68] R. L. Kuo *et al.*, “Interactome Analysis of the NS1 Protein Encoded by Influenza A H1N1 Virus Reveals a Positive Regulatory Role of Host Protein PRP19 in Viral Replication,” *J. Proteome Res.*, vol. 15, no. 5, pp. 1639–1648, May 2016, doi: 10.1021/ACS.JPROTEOME.6B00103.

[69] N. Jorba *et al.*, “Analysis of the interaction of influenza virus polymerase complex with human cell factors,” *Proteomics*, vol. 8, no. 10, pp. 2077–2088, May 2008, doi: 10.1002/PMIC.200700508.

[70] L. Lin *et al.*, “Identification of RNA helicase A as a cellular factor that interacts with influenza A virus NS1 protein and its role in the virus life cycle,” *J. Virol.*, vol. 86, no. 4, pp. 1942–1954, Feb. 2012, doi: 10.1128/JVI.06362-11.

[71] B. K. Dove *et al.*, “A quantitative proteomic analysis of lung epithelial (A549) cells infected with 2009 pandemic influenza A virus using stable isotope labelling with amino acids in cell culture,” *Proteomics*, vol. 12, no. 9, pp. 1431–1436, May 2012, doi: 10.1002/PMIC.201100470.

[72] J. Sun, J. C. Vera, J. Drnevich, Y. T. Lin, R. Ke, and C. B. Brooke, “Single cell heterogeneity in influenza A virus gene expression shapes the innate antiviral response to infection,” *PLoS Pathog.*, vol. 16, no. 7, Jul. 2020, doi: 10.1371/JOURNAL.PPAT.1008671.

[73] N. Schmidt *et al.*, “The SARS-CoV-2 RNA-protein interactome in infected human cells,” *Nat. Microbiol.*, vol. 6, no. 3, pp. 339–353, Mar. 2021, doi: 10.1038/S41564-020-00846-Z.

[74] S. Zhang *et al.*, “Comparison of viral RNA-host protein interactomes across pathogenic RNA viruses informs rapid antiviral drug discovery for SARS-CoV-2,” *Cell Res.*, vol. 32, no. 1, pp. 9–23, Jan. 2022, doi: 10.1038/S41422-021-00581-Y.

[75] J. Baggen *et al.*, “Genome-wide CRISPR screening identifies TMEM106B as a proviral host factor for SARS-CoV-2,” *Nat. Genet.*, vol. 53, no. 4, pp. 435–444, 2021, doi: 10.1038/s41588-021-00805-2.

[76] Y. Zhu *et al.*, “A genome-wide CRISPR screen identifies host factors that regulate SARS-CoV-2 entry,” *Nat. Commun.*, vol. 12, no. 1, Dec. 2021, doi: 10.1038/S41467-021-21213-4.

[77] J. Wei *et al.*, “Genome-wide CRISPR Screens Reveal Host Factors Critical for SARS-CoV-2 Infection,” *Cell*, vol. 184, no. 1, pp. 76-91.e13, 2021, doi: 10.1016/j.cell.2020.10.028.

[78] R. Wang *et al.*, “Genetic Screens Identify Host Factors for SARS-CoV-2 and Common Cold Coronaviruses,” *Cell*, vol. 184, no. 1, pp. 106-119.e14, 2021, doi: 10.1016/j.cell.2020.12.004.

[79] W. M. Schneider *et al.*, “Genome-Scale Identification of SARS-CoV-2 and Pan-coronavirus Host Factor Networks,” *Cell*, vol. 184, no. 1, pp. 120-132.e14, 2021, doi: 10.1016/j.cell.2020.12.006.

[80] A. Rebendenne *et al.*, “Bidirectional genome-wide CRISPR screens reveal host factors regulating SARS-CoV-2, MERS-CoV and seasonal HCoVs,” *Nat. Genet.*, vol. 54, no. 8, pp. 1090–1102, 2022, doi: 10.1038/s41588-022-01110-2.

[81] M. Israeli *et al.*, “Genome-wide CRISPR screens identify GATA6 as a proviral host factor for SARS-CoV-2 via modulation of ACE2,” *Nat. Commun.*, vol. 13, no. 1, 2022, doi: 10.1038/s41467-022-29896-z.

[82] M. Grodzki *et al.*, “Genome-scale CRISPR screens identify host factors that promote human coronavirus infection,” *Genome Med.*, vol. 14, no. 1, pp. 1–18, 2022, doi: 10.1186/s13073-022-01013-1.

[83] Z. Daniloski *et al.*, “Identification of Required Host Factors for SARS-CoV-2 Infection in Human Cells,” *Cell*, vol. 184, no. 1, pp. 92-105.e16, 2021, doi: 10.1016/j.cell.2020.10.030.

[84] S. B. Biering *et al.*, “Genome-wide bidirectional CRISPR screens identify mucins as host factors modulating SARS-CoV-2 infection,” *Nat. Genet.*, vol. 54, no. 8, pp. 1078–1089, 2022, doi: 10.1038/s41588-022-01131-x.

[85] J. Hou *et al.*, “Integrated multi-omics analyses identify key anti-viral host factors and pathways controlling SARS-CoV-2 infection,” *Res. Sq.*, Aug. 2022, doi: 10.21203/RS.3.RS-1910932/V1.

[86] A. P. Ugalde *et al.*, “Autophagy-linked plasma and lysosomal membrane protein PLAC8 is a key host factor for SARS-CoV-2 entry into human cells,” *EMBO J.*, vol. 41, no. 21, Nov. 2022, doi: 10.15252/EMBJ.2022110727.

[87] M. Yousefi *et al.*, “Betacoronaviruses SARS-CoV-2 and HCoV-OC43 infections in IGROV-1 cell line require aryl hydrocarbon receptor,” *Emerg. Microbes Infect.*, vol. 12, no. 2, 2023, doi: 10.1080/22221751.2023.2256416.

[88] H. H. Hoffmann *et al.*, “Functional interrogation of a SARS-CoV-2 host protein interactome identifies unique and shared coronavirus host factors,” *Cell Host Microbe*, vol. 29, no. 2, pp. 267-280.e5, Feb. 2021, doi: 10.1016/j.chom.2020.12.009.

[89] A. Pagis *et al.*, “Genome-wide loss-of-function screen using human pluripotent stem cells to study virus-host interactions for SARS-CoV-2,” *Stem Cell Reports*, vol. 18, no. 9, pp. 1766–1774, Sep. 2023, doi: 10.1016/j.stemcr.2023.07.003.

[90] I. Frasson *et al.*, “Identification of druggable host dependency factors shared by multiple SARS-CoV-2 variants of concern,” *J. Mol. Cell Biol.*, vol. 16, no. 3, Mar. 2024, doi: 10.1093/JMCB/MJAE004.

[91] M. Sakai *et al.*, “Genome-scale CRISPR-Cas9 screen identifies host factors as potential therapeutic targets for SARS-CoV-2 infection,” *iScience*, vol. 27, no. 8, Aug. 2024, doi: 10.1016/j.isci.2024.110475.

[92] F. Rehfeld, J. L. Eitson, M. B. Ohlson, T. C. Chang, J. W. Schoggins, and J. T. Mendell, “CRISPR screening reveals a dependency on ribosome recycling for efficient SARS-CoV-2 programmed ribosomal frameshifting and viral replication,” *Cell Rep.*, vol. 42, no. 2, Feb. 2023, doi: 10.1016/j.celrep.2023.112076.

[93] J. Li *et al.*, “Virus-Host Interactome and Proteomic Survey Reveal Potential Virulence Factors Influencing SARS-CoV-2 Pathogenesis,” *Med*, vol. 2, no. 1, pp. 99-112.e7, Jan. 2021, doi: 10.1016/j.medj.2020.07.002.

[94] D. K. Kim *et al.*, “A proteome-scale map of the SARS-CoV-2-human contactome,” *Nat. Biotechnol.*, vol. 41, no. 1, pp. 140–149, Jan. 2023, doi: 10.1038/S41587-022-01475-Z.

[95] C. Bamberger, S. Pankow, S. Martínez-Bartolomé, J. K. Diedrich, R. S. K. Park, and J. R. Yates, “Analysis of the Tropism of SARS-CoV-2 Based on the Host Interactome of the Spike Protein,” *J. Proteome Res.*, vol. 22, no. 12, pp. 3742–3753, Dec. 2023, doi: 10.1021/ACS.JPROTEOME.3C00387.

[96] F. Pahmeier *et al.*, “Identification of host dependency factors involved in SARS-CoV-2 replication organelle formation through proteomics and ultrastructural analysis,” *J. Virol.*, vol. 97, no. 11, Nov. 2023, doi: 10.1128/JVI.00878-23.

[97] Y. Zhou *et al.*, “A comprehensive SARS-CoV-2-human protein-protein interactome reveals COVID-19 pathobiology and potential host therapeutic targets,” *Nat. Biotechnol.*, vol. 41, no. 1, pp. 128–139, Jan. 2023, doi: 10.1038/S41587-022-01474-0.

[98] Z. Yang *et al.*, “Interaction between host G3BP and viral nucleocapsid protein regulates SARS-CoV-2 replication and pathogenicity,” *Cell Rep.*, vol. 43, no. 3, Mar. 2024, doi: 10.1016/j.celrep.2024.113965.

[99] Z. Chen *et al.*, “Interactomes of SARS-CoV-2 and human coronaviruses reveal host factors potentially affecting pathogenesis,” *EMBO J.*, vol. 40, no. 17, Sep. 2021, doi: 10.15252/EMBJ.2021107776.

[100] S. Nabeel-Shah *et al.*, “SARS-CoV-2 nucleocapsid protein binds host mRNAs and attenuates stress granules to impair host stress response,” *iScience*, vol. 25, no. 1, Jan. 2022, doi: 10.1016/j.isci.2021.103562.

[101] D. E. Gordon *et al.*, “A SARS-CoV-2 protein interaction map reveals targets for drug repurposing,” *Nature*, vol. 583, no. 7816, pp. 459–468, 2020, doi: 10.1038/s41586-020-2286-9.

[102] A. Stukalov *et al.*, “Multilevel proteomics reveals host perturbations by SARS-CoV-2 and SARS-CoV,” *Nature*, vol. 594, no. 7862, pp. 246–252, Jun. 2021, doi: 10.1038/S41586-021-03493-4.

[103] N. G. Ravindra *et al.*, “Single-cell longitudinal analysis of SARS-CoV-2 infection in human airway epithelium identifies target cells, alterations in gene expression, and cell state changes,” *PLoS Biol.*, vol. 19, no. 3, Mar. 2021, doi: 10.1371/JOURNAL.PBIO.3001143.

[104] E. Wyler *et al.*, “Transcriptomic profiling of SARS-CoV-2 infected human cell lines identifies HSP90 as target for COVID-19 therapy,” *iScience*, vol. 24, no. 3, p. 102151, Mar. 2021, doi: 10.1016/J.ISCI.2021.102151.

[105] J. Fang *et al.*, “Staufen1 Interacts with Multiple Components of the Ebola Virus Ribonucleoprotein and Enhances Viral RNA Synthesis,” *mBio*, vol. 9, no. 5, Sep. 2018, doi: 10.1128/MBIO.01771-18.

[106] M. Gong *et al.*, “Genome-wide CRISPR/Cas9 screen identifies SLC39A9 and PIK3C3 as crucial entry factors for Ebola virus infection,” *PLoS Pathog.*, vol. 20, no. 8, Aug. 2024, doi: 10.1371/JOURNAL.PPAT.1012444.

[107] M. Flint *et al.*, “A genome-wide CRISPR screen identifies N-acetylglucosamine-1-phosphate transferase as a potential antiviral target for Ebola virus,” *Nat. Commun.*, vol. 10, no. 1, Dec. 2019, doi: 10.1038/S41467-018-08135-4.

[108] R. J. Carlson *et al.*, “Single-cell image-based genetic screens systematically identify regulators of Ebola virus subcellular infection dynamics,” *bioRxiv*, Apr. 2024, doi: 10.1101/2024.04.06.588168.

[109] J. E. Carette *et al.*, “Ebola virus entry requires the cholesterol transporter Niemann-Pick C1,” *Nature*, vol. 477, no. 7364, pp. 340–343, Sep. 2011, doi: 10.1038/NATURE10348.

[110] S. Martin *et al.*, “A genome-wide siRNA screen identifies a druggable host pathway essential for the Ebola virus life cycle,” *Genome Med.*, vol. 10, no. 1, Aug. 2018, doi: 10.1186/S13073-018-0570-1.

[111] D. S. Yu *et al.*, “Chaperones, Membrane Trafficking and Signal Transduction Proteins Regulate Zaire Ebola Virus trVLPs and Interact With trVLP Elements,” *Front. Microbiol.*, vol. 9, no. NOV, Nov. 2018, doi: 10.3389/FMICB.2018.02724.

[112] J. Batra *et al.*, “Protein Interaction Mapping Identifies RBBP6 as a Negative Regulator of Ebola Virus Replication,” *Cell*, vol. 175, no. 7, pp. 1917-1930.e13, Dec. 2018, doi: 10.1016/j.cell.2018.08.044.

[113] J. Chen *et al.*, “Host factor SMYD3 is recruited by Ebola virus nucleoprotein to facilitate viral mRNA transcription,” *Emerg. Microbes Infect.*, vol. 8, no. 1, pp. 1347–1360, Jan. 2019, doi: 10.1080/22221751.2019.1662736.

[114] M. J. Morwitzer *et al.*, “Identification of RUVBL1 and RUVBL2 as Novel Cellular Interactors of the Ebola Virus Nucleoprotein,” *Viruses*, vol. 11, no. 4, Apr. 2019, doi: 10.3390/V11040372.

[115] I. García-Dorival *et al.*, “Elucidation of the Ebola virus VP24 cellular interactome and disruption of virus biology through targeted inhibition of host-cell protein function,” *J. Proteome Res.*, vol. 13, no. 11, pp. 5120–5135, Nov. 2014, doi: 10.1021/PR500556D.

[116] S. Gurumayum *et al.*, “OGEE v3: Online GEne Essentiality database with increased coverage of organisms and human cell lines,” *Nucleic Acids Res.*, vol. 49, no. D1, pp. D998–D1003, Jan. 2021, doi: 10.1093/NAR/GKAA884.

[117] Y. Liao *et al.*, “Lysine-specific histone demethylase complex restricts Epstein-Barr virus lytic reactivation,” *Nat. Microbiol.*, vol. 10, no. 12, pp. 3290–3304, Dec. 2025, doi: 10.1038/s41564-025-02165-7.

[118] Y. Ma *et al.*, “CRISPR/Cas9 Screens Reveal Epstein-Barr Virus-Transformed B Cell Host Dependency Factors,” *Cell Host Microbe*, vol. 21, no. 5, pp. 580-591.e7, May 2017, doi: 10.1016/j.chom.2017.04.005.

[119] S. P. T. Yiu, C. Zerbe, D. Vanderwall, E. L. Huttlin, M. P. Weekes, and B. E. Gewurz, “An Epstein-Barr virus protein interaction map reveals NLRP3 inflammasome evasion via MAVS UFMylation,” *Mol. Cell*, vol. 83, no. 13, pp. 2367-2386.e15, Jul. 2023, doi: 10.1016/j.molcel.2023.05.018.

[120] M. A. Calderwood *et al.*, “Epstein-Barr virus and virus human protein interaction maps,” *Proc. Natl. Acad. Sci. U. S. A.*, vol. 104, no. 18, pp. 7606–7611, May 2007, doi: 10.1073/pnas.0702332104.
